# Supplementary material for: Exploring and mapping chemical space with molecular assembly trees
Source: Sci Adv. 2021 Sep 24;7(39):eabj2465. doi: 10.1126/sciadv.abj2465 (PMC8462901; doi:10.1126/sciadv.abj2465)
Supplement: 20210924-1 [file sciadv.abj2465.v1.pdf]

CHEMISTRY

Exploring and mapping chemical space with molecular assembly trees

Yu Liu, Cole Mathis, Michał Dariusz Bajczyk, Stuart M. Marshall, Liam Wilbraham, Leroy Cronin*

Copyright © 2021 The Authors, some rights reserved; exclusive licensee American Association for the Advancement of Science. No claim to original U.S. Government Works. Distributed under a Creative Commons Attribution License 4.0 (CC BY).

The rule-based search of chemical space can generate an almost infinite number of molecules, but exploration of known molecules as a function of the minimum number of steps needed to build up the target graphs promises to uncover new motifs and transformations. Assembly theory is an approach to compare the intrinsic complexity and properties of molecules by the minimum number of steps needed to build up the target graphs. Here, we apply this approach to prebiotic chemistry, gene sequences, plasticizers, and opiates. This allows us to explore molecules connected to the assembly tree, rather than the entire space of molecules possible. Last, by developing a reassembly method, based on assembly trees, we found that in the case of the opiates, a new set of drug candidates could be generated that would not be accessible via conventional fragment-based drug design, thereby demonstrating how this approach might find application in drug discovery.

INTRODUCTION

Chemical space is populated by a vast range of compounds, which can be characterized by their molecular composition, formula, graph representation, and reactivity (*1*, *2*). The generation of molecules via their graphs can be enumerated to give an unimaginably vast number of at least 10^{60} small organic molecules (*1*, *3*, *4*), but this is unrealistic, as many of these molecules might be unstable or inaccessible synthetically. However, when exploring biochemistry, only a few hundred different types of "unique" small molecules are needed by the simplest living organisms (*1*, *5*), indicating that the chemical space relevant to biology on Earth is a tiny fraction of chemical space that is possible (*6*, *7*). For example, many of the related structures between these known compounds are undiscovered and unknown (e.g., opiates and cannabinoids are found to occur in related clusters of structures but no cross between these structure types are known). The issue therefore arises about how the space of molecules can be effectively searched (*1*, *2*, *8*) and what constrains molecules to be both thermodynamically possible and biologically accessible (*2*, *9*–*11*) because although many molecules are physically possible, the number of molecules accessible by the current machinery of biology is smaller (*9*, *12*–*15*).

One way to explore the universe of molecules is to construct a chemical space as a dataset, e.g., GDB-17 database (*16*, *17*), DrugBank (*18*), and PubChem (*19*), and then navigate the dataset using molecular descriptors (*20*–*23*). Searching these databases is inefficient because it requires exhaustively enumerating [or Bayesian optimization (*24*)] and screening molecules for desirable properties. Similarly, it is possible to iteratively generate chemical subspaces, followed by filtering unwanted structures, until desired molecules are obtained. This can be done using a genetic algorithm (*25*, *26*), extrapolation techniques (*27*, *28*), or even using human intuition (*29*). However, given the relative sizes of possible chemical space, compared to the number of interesting molecular structures, it is not clear how comprehensive any of these approaches will be. Recently, machine learning and statistical techniques have been introduced into the navigation of chemical space (*30*, *31*). For example, hundreds of thousands of existing chemical structures were used to train a deep neural network so that each molecule can be assigned to a set of discrete coordinates in the continuous latent space of the neural network. Navigating within this discrete chemical space corresponds to navigating in the continuous latent space, which is much easier computationally (*32*). Alternatively, a convolutional neural network can be trained directly on graph representations of molecules to infer their molecular features, and these can have a better predictive performance over the existing hand-crafted fingerprints in some applications (*33*). These approaches offer improvement over raw enumeration and filtering because they compress the search space. However, while neural networks might make the navigation of chemical space more efficient, the space is obscured and important contingent information is not accessible (*1*, *2*). One important question is how the current structure of observable chemistry relates to the space of biology. This is interesting because evolution has selected the machinery of biochemistry over the past 4 billion years on Earth (*1*, *10*, *11*, *34*). By constructing the assembly tree from molecular structures, we will be able to not only use a molecular-based route to explore the information used to assemble the molecules found in biology but also infer which molecules are more likely to have shared pathways (i.e., infer the presence of new biological pathways). This is another important window not only on how the process of evolution by random selection leads to both conservation and reuse of biochemical pathways but also in the generation of novelty.

RESULTS AND DISCUSSION

Establishing the theory of assembly spaces of molecules

Assembly theory (*35*, *36*) quantifies the constraints required to produce a molecule by measuring the minimum number of steps to produce the molecular graph thereof. Here, we apply this approach to explore the structure of chemical space and suggest a way to generate new compounds from the assembly space (see Fig. 1). In the adenine example, we chose the four chemical bonds that make molecule, namely, C—C, C=C, C—N, and C=N, as the basic building blocks. We call these building blocks and the molecular structures that will be produced therefrom the assembly building blocks and call the set of all assembly building blocks the assembly pool. In an assembly pool, any type of assembly building block is assumed to

School of Chemistry, University of Glasgow, University Avenue, Glasgow G12 8QQ, UK.
*Corresponding author. Email: lee.cronin@glasgow.ac.uk

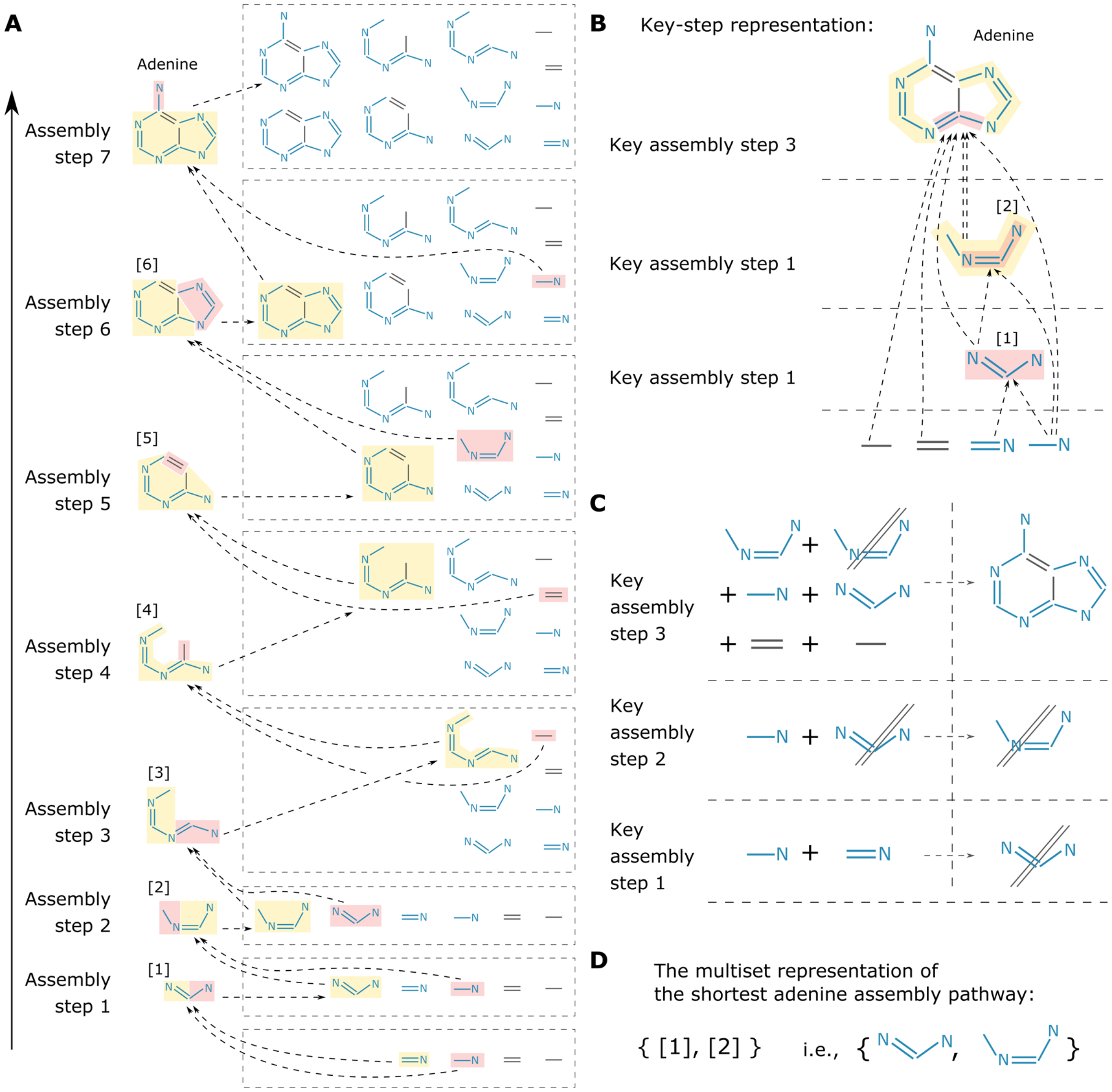

Fig. 1. Representations of an assembly pathway, by taking adenine as an example. (**A**) One of the many assembly pathways of adenine (it turns out to be the shortest one, according to our MC algorithm, explained later). The assembly pool (shown inside the dashed boxes) evolves with each assembly step. The colors denote which two assembly building blocks are used to make the new one (note that the color schemes are independent for each step). (**B**) The key-step representation of the assembly pathway. (**C**) The joint process for each key assembly step, which is used to work out the multiset representation. (**D**) The multiset representation of this assembly pathway. Strictly speaking, it should be written as $\{[1]^1, [2]^1\}$ where the superscript "1" is the multiplicity of this assembly building block, that is, after canceling out, it appears once on the left-hand side of (C), but for simplicity, we only explicitly write down the multiplicity when it is not 1.

have infinite instances. One assembly step is precisely defined as the three sequential operations: (i) take two assembly building blocks from the assembly pool, (ii) join the two together in a way based on the particular system in question (in this context, that is, to superimpose certain atoms from the two building blocks to make a larger molecular structure), and (iii) add the new composite building block to the assembly pool. Once a sequence of the assembly steps can successfully construct the target molecule, this is defined as an assembly pathway for this molecule. Figure 1A shows one of many assembly pathways of adenine, which has seven assembly steps after which adenine is constructed and appears in the assembly pool and gives an assembly index of 7. Last, the assembly space of a molecule refers to all the assembly building blocks included in the assembly pathways. The assembly index of the shortest pathway to construct a molecule is referred to as the molecular assembly number (MA) of the molecule. The assembly pathway shown in Fig. 1A is the shortest one, calculated out by the Monte Carlo (MC) algorithm that we have developed (which is guaranteed to give the shortest assembly pathway when the computing time is sufficiently long; see section S3 for details), and adenine's MA is thus 7.

In assembly pathways, the order of some steps can be switched without changing the length of the pathway, which leads to a combinatorically large number of trivial pathways that all have the same number of steps. For instance, as the pathway shown in Fig. 1A

starts from building block [3] to make building block [6], we can either add a C—C bond to [3], then a C═C, and lastly add building block [2], just as the figure shows, or we can add [2] first, then a C═C, and, last, a C—C, which leads to another pathway. Taking Fig. 1A as an example, building block [1] must be made before [2] and [3] because [1] is used to make [2] and [3]; by the same logic, building block [2] must be made before [3] and [6] because [2] is used to make them. We can use these properties to represent assembly pathways without ambiguity by focusing on the steps in which order matters. We call those special building blocks that define the hierarchical relationships among the chemical structures the key assembly building blocks (they are also the assembly building blocks that are used more than once in the pathway) and their corresponding steps as the key assembly steps.

Therefore, we can represent a pathway in terms of key steps, which eliminates all the trivial information. For example, Fig. 1B is the key-step representation of the pathway shown in Fig. 1A, and the key building blocks are [1] and [2]. The number of key building blocks can be elucidated from the key-step representation. We can explicitly write down the joint process for each key step and then remove the building blocks that appear on both sides (Fig. 1C). The building blocks left over constitute the target molecule nonrepetitively. Specifically, the target molecule adenine can be made from these chemical bonds and structures nonrepetitively and by the least number of assembly steps. The information of the basic building blocks is trivial and can thus be omitted. So, we lastly obtained the multiset representation of this assembly pathway, as shown in Fig. 1D (see section S2). The multiset representation can be readily determined from the key-step representation without ambiguity and vice versa. The latter emphasizes the hierarchical relationship between the building blocks, while the former emphasizes the information of constituents and provides a compact summary of the assembly space associated with this pathway. An assembly pathway of a molecule does not necessarily correspond to a realistic sequence of chemical reactions that produce this molecule. Instead, the shortest assembly pathway bounds the likelihood of the molecule forming probabilistically (which means that if the shortest assembly pathways of two molecules overlap, then they are likely to have shared synthetic pathways). No matter which methods or synthetic approaches are used, there will be no shorter way than this ideal one, which makes it an intrinsic property of a molecule.

Molecular assembly trees

The concept of assembly pathways and spaces can be naturally applied to two molecules, which allows us to look at the shortest assembly pathways that construct both simultaneously. In general, the shared shortest assembly pathway of A and B is not the union of the individual shortest assembly pathways of A and B. As an example, consider adenine and another nucleobase thymine (Fig. 2A). The shortest pathway of adenine alone is indicated by the blue dashed arrows on the left whose *MA* is 7 (the same pathway as in Fig. 1).

The shortest pathway of thymine alone is indicated by the red dashed arrows on the right, which can be written in multiset representation as {[7]}, and its *MA* is calculated to be 6. However, the shortest assembly pathway to make adenine and thymine altogether is indicated by the black dashed arrows in the middle, which does not overlap with either of the shortest pathways. It is $\{[8]^2, [9]\}$ in multiset representation (where the superscript "2" is the multiplicity of [8]), and its pathway index is calculated to be 12, which is smaller than 7 + 6, the sum of the two individual *MA*s. Molecular assembly theory can be extended further to three or more molecules, which allows us to look at the shortest assembly pathways that construct a group of molecules. The multimolecular assembly spaces tend to have a tree-like structure where different branches lead to

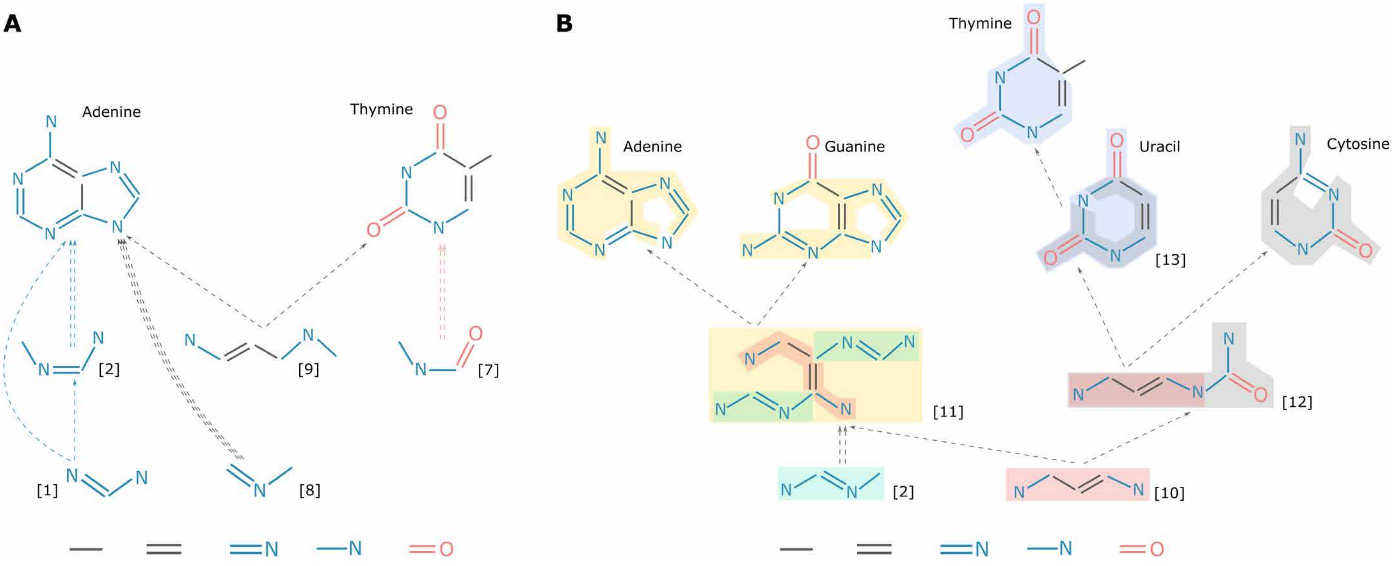

Fig. 2. Two exemplified molecular assembly trees. (**A**) The assembly space of adenine and thymine. The shortest assembly pathway for adenine alone is indicated by the blue dashed arrows, while the shortest assembly pathway for thymine alone is indicated by the red dashed arrows. The shortest assembly pathway to make adenine and thymine altogether is the one indicated by the black dashed arrows. (**B**) A molecular assembly tree for A, G, T, U, and C, which can also be written as {[2, 10, 11, 12, 13]}, whose index is calculated to be 16. Note that, in both (A) and (B), the colors are just used to help the reader recognize the building blocks, and the color schemes are independent; we also omitted the arrows starting from the basic building blocks for a better visualization.

different molecules (see Fig. 2A), but the number of key building blocks is still relatively small. Therefore, we refer to the shortest assembly pathways to make a group of molecules altogether as the molecular assembly tree (assembly tree for short) thereof and refer to its index as assembly tree *MA*.

As an example, we built an assembly tree for the five nucleobases: adenine (A), guanine (G), thymine (T), uracil (U), and cytosine (C). We first extended the MC algorithm that we have developed for a single molecule to a group of molecules (see details in section S4). We then use this extended algorithm to compute the assembly tree and then visualize the tree manually, as shown in Fig. 2B (see section S4.3). The *MA* is 16, but it takes 43 steps to build the molecules bond by bond, and the minimum number of steps to construct them separately using an assembly process is 33. This relatively low *MA* reflects the fact that they share lots of common structures (even the common structures share quite a few common substructures), resulting in the hierarchy shown by the assembly tree, which represents a highly related subset of chemical space.

Biomolecules

For this study, we picked a dozen vital biomolecules to construct their assembly tree, including the five nucleobases (A, G, T, C, and U), pyruvate [a key intermediate in metabolic pathways across various organisms (*37*)], and citrate [an intermediate of the vital metabolic pathway, the Krebs cycle, used by all aerobic organisms to release energy (*38*, *39*)]. In addition, we included D-ribose [the carbohydrate that serves as the backbone of RNA among various other functions (*40*, *41*)], nicotinamide adenine dinucleotide [NAD^+; a vital cofactor that carries electrons from one reaction to another (*42*)], adenosine diphosphate (ADP), adenosine triphosphate (ATP), and a symbolic RNA molecule. As we see in Fig. 3, there are lots of structures that are shared. We can imagine that as we include more biomolecules in the tree (e.g., various proteins and RNA and DNA sequences), more structures will be shared, and the tree will grow deeper and deeper but without growing much wider (i.e., having more hierarchical layers but not many nonrelated key blocks being added to the tree). This potentially "narrow" assembly tree is an indication that all of the vital biomolecules involved in extant life on Earth is not arbitrary but a consequence of millions of years of evolution.

In origin of life studies, one central problem is that most biomolecules are complex and thus seem very unlikely to emerge de novo from prebiotic chemistry, while life requires them altogether to function properly. An important clue that we gain from this study is that lots of chemical substructures are shared among these vital biomolecules. Hence, it can be argued that the set of processes producing these molecules together, as a set, could have been smaller than if they were produced de novo individually because they could have shared common pathways. The closer the biomolecules are in the assembly tree (i.e., they are more related), the easier it is to access

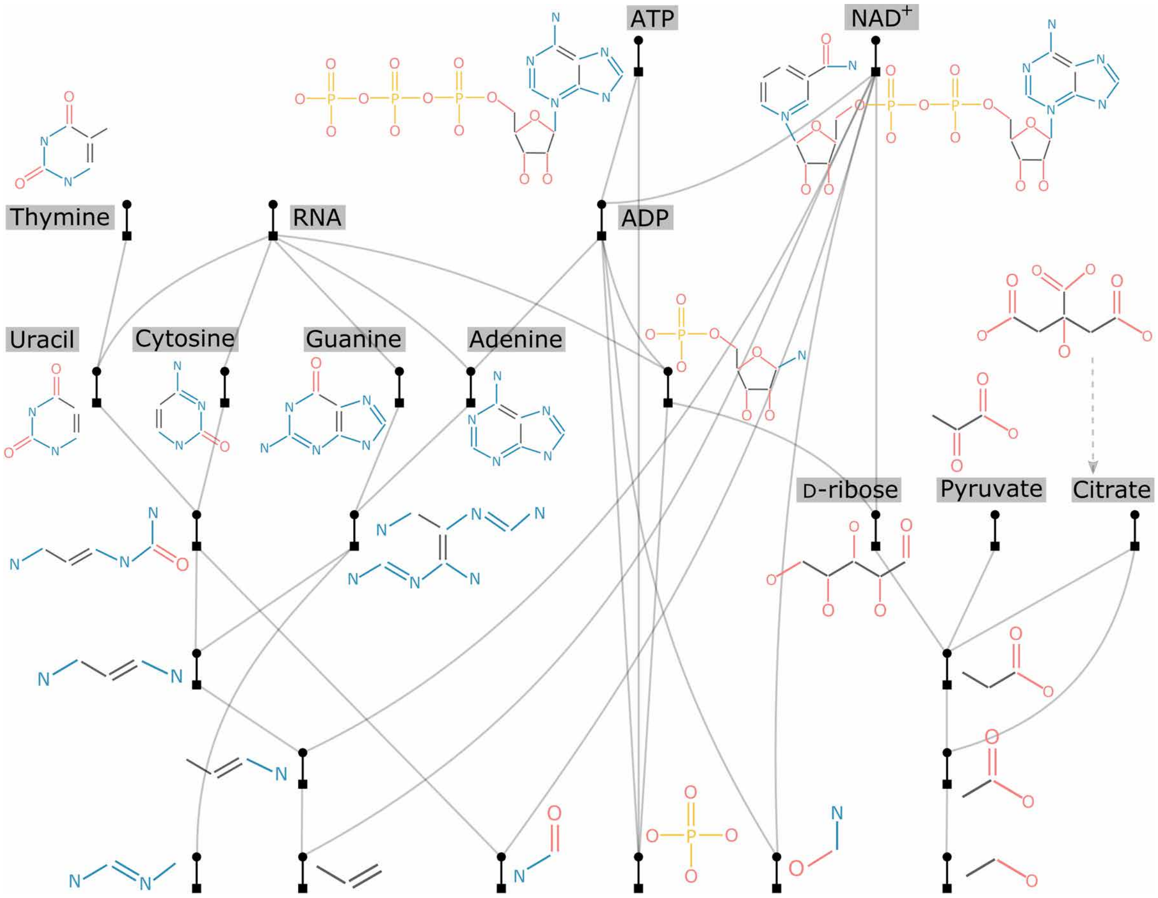

Fig. 3. The assembly tree of a dozen vital biomolecules, including the five nucleobases (A, G, T, C, and U), pyruvate, citrate, D-ribose, NAD^+, ADP, ATP, and a symbolic RNA molecule.

these compounds. This is arguably why biomolecules exploited by extant life appear close in the assembly tree; otherwise, they would be too complex to emerge individually. For example, the fact that the five nucleobases AGTCU are closely related in the assembly tree indicates that it is not arbitrary nor a result of a frozen event that they serve as the fundamental units of the genetic code across all life on Earth and that the molecules may have been incorporated into protobiological systems because of their structural relatedness. We tested this idea by building assembly trees for alternated nucleobases (see section S5 for details). We found that *MA*s of alternated trees are always much higher (ranging from 31 to 38) than the one in reality, which is 16, as mentioned in the previous section. This analysis only indicates that the extant set of nucleobases were selected because of their relatedness, but why nature selects this particular set is another question since there could be many such sets. This intriguing and important question requires a much wider exploration of chemical space, yet we believe our methods provide a useful tool for framing this question.

Gene sequences

So far, we have focused on constructing assembly trees for molecules, but our theory can equally be applied to gene sequences. We take one hypothetical gene sequence X (60 bases; Fig. 4) as an example to illustrate how we can make use of the compressed information carried by X's assembly tree to reconstruct itself with less efforts. The naïve method is to add one base at one time, and then, 59 steps in total are needed to construct X, but notice that some segments are repeated multiple times. If it is possible to produce these repetitive segments beforehand, then it is possible to take them directly and combine them with other segments in a precise way to obtain X, and this requires less than 59 steps [as producing repetitive segments is relatively easy (*43*), e.g., polymerase chain reaction, the reconstruction of the original sequence takes less efforts than the naïve method]. As the assembly tree can filter all of the repetitive and redundant information (i.e., record the information of the sequence in the most compressed way), the information of this "precise way" is completely stored in the assembly tree. Then, we build X's assembly tree, as shown in Fig. 4 (in this example, we only deal with one sequence X, and its assembly tree reduces to its shortest assembly pathway). Note that in this gene sequence case, we use nucleobases as the basic building blocks rather than chemical bonds as in the molecule cases.

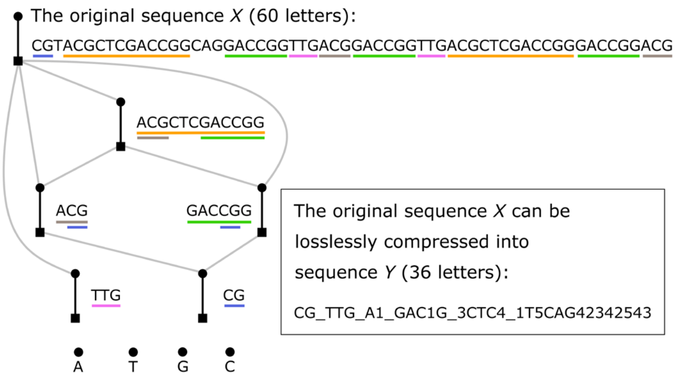

Fig. 4. The assembly tree of one hypothetical gene sequence *X* (as, here, we only deal with one sequence *X*, it reduces to its shortest assembly pathway in the key-step representation). Here, we use nucleobases as the basic building blocks rather than chemical bonds in the molecule cases. Thus, we explicitly draw those nucleobases at the bottom for clarification.

Before reconstructing, we introduce a way to compress the information hold in the assembly tree into a single string, which would be very useful in future studies, as the assembly tree of genomes would be huge and directly storing the tree structure is neither convenient nor efficient (note that while preserving the assembly tree information is our priority, we are not intended to defeat any sophisticated data compression technique per se). The assembly tree in Fig. 4 can be rewritten as CG_TTG_A1_GAC1G_3CTC4_1T5CAG42342543, denoted as Y. Now, we can reconstruct X based on Y. The first step is to construct CG by simply combining the individual bases C and G (one step) and construct TTG by combining the individual bases T, T, and G (two steps). Second, construct A1, where "1" stands for the first segment in Y, which is CG. Thus, we only need one step to obtain ACG since CG has been constructed before. Third, construct GAC1G, where we can reuse "1" again, and we thus need four steps. Fourth, construct 3CTC4, where we can reuse "3" (A1, the third segment in Y) and "4" (GAC1G, the fourth segment in Y), and we thus need four steps. Last, we can construct the original sequence X based on the last part of Y, namely, "1T5CAG42342543" where the integer stands for the corresponding segment that has been obtained before and can thus be reused. Thus, we need 13 steps here. In total, we need 1 + 2 + 1 + 4 + 4 + 13 = 25 steps, which is much less than the naïve 59 steps. Last, to quantify the increase of information from the original sequence X (60 letters) to the lossless and compressed version Y (36 letters), we can use Shannon entropy (*44*, *45*), a widely used quantity to describe the information of a string. Shannon entropy H of a string X (with n letters) is defined as $H(X) = -\sum_x p(x) \cdot \log_2 p(x)$, where $p(x)$ is the probability that the letter x appears in this sequence X, which is set to be equal to the times x appears in X divided by n, and the sum goes through every distinct letter. Therefore, we obtained that Shannon entropy of the original sequence $H(X)$ is 1.851, while $H(Y)$ is 3.251, increased by 1.76 times.

We do think that the application of assembly theory to gene assembly has potential application to not only building new routes to engineer synthetic genomes by taking a series of genes, finding the common parts, and then finding the minimal route to assemble these parts to access all of the desired genes. Not only could this be used to efficiently build new function, but it also has promise to explore how evolution has reused genetic motifs beyond the current modular understanding and perhaps find more complex and conserved routes for the propagation of genetic information across different genes.

Plasticizers

Plasticizers are added to polymers and formulations to make them more plastic, to decrease viscosity and friction, and to increase flexibility (*46*), but they can leach into the environment. This is a problem since these compounds have been shown to be toxic (*47*). One big issue is that, in general, the evaluation of specific effects and prevalence of plasticizer molecules in the environment is hard, as so many different types are in use and many degradation pathways exist (*47*). This means that potentially vast numbers of molecules related to the plasticizer parent are present in the environment, posing similar or even greater health risks than the parent. However, by exploring the assembly tree of plasticizer molecules (see Fig. 5), it might be possible to map the molecules that are potentially contaminated and even identify unknown or unexpected molecules.

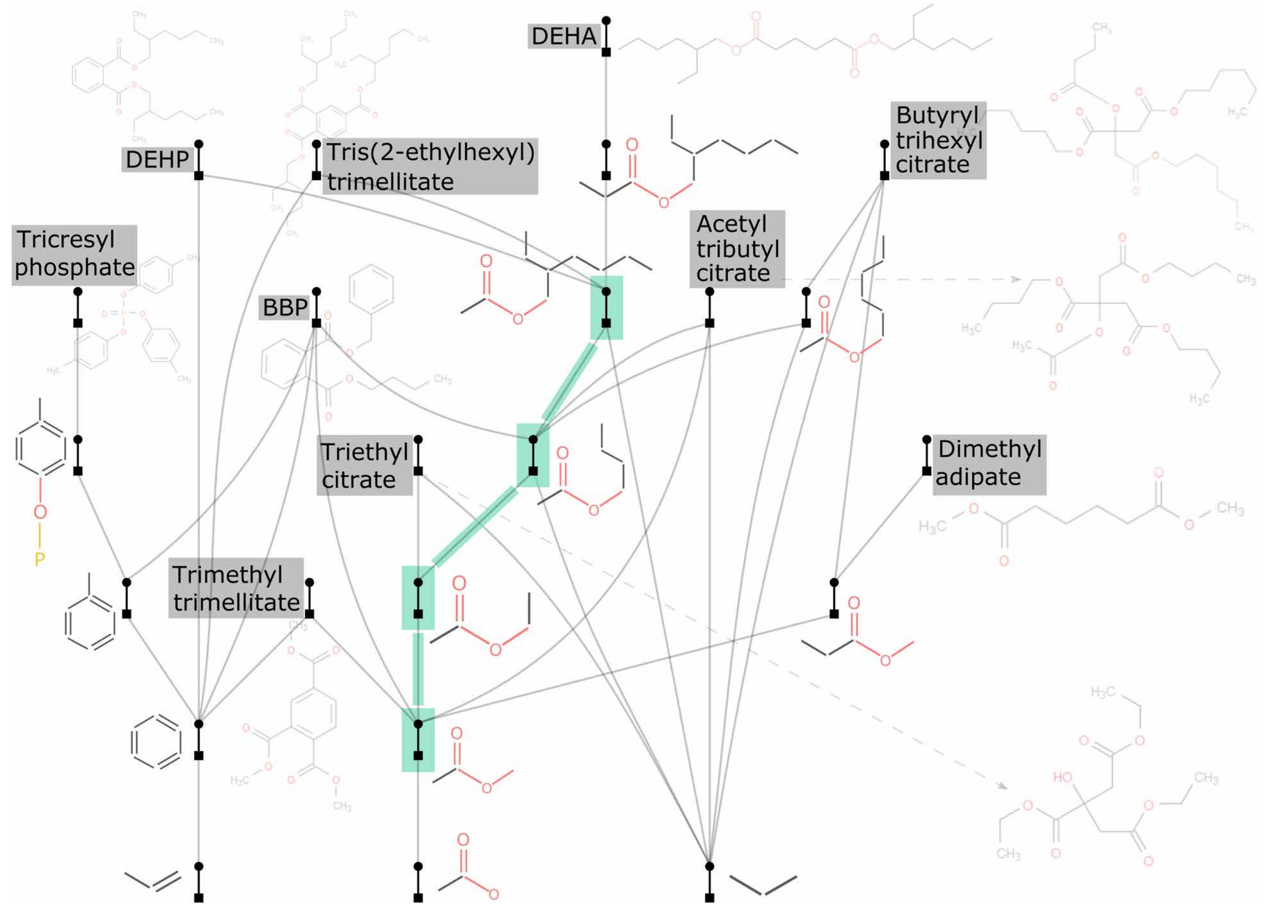

Fig. 5. The assembly tree of 10 commonly used plasticizers including BBP, DEHP, DEHA, and others. For a clearer visualization, all plasticizers are made dimmer than other parts of the tree. The most central structures are highlighted green.

Here, we built the assembly tree of 10 commonly used plasticizers including BBP (benzyl butyl phthalate), DEHP (di-2-ethylhexyl phthalate), DEHA [bis(2-ethylhexyl) adipate], and others. As seen from Fig. 5, these 10 seemingly distinct molecules share lots of common structures (i.e., key assembly building blocks), which are also highly related. These 10 relatively large plasticizers are constructed by only a dozen of these key building blocks. In particular, the four central structures highlighted green directly or indirectly connect to most of them (except for tricresyl phosphate) and are highly related themselves. We can thus imagine that if we can detect and identify these central/characteristic chemical structures and fragments in an environment [e.g., using the mass spectrometry technique for assembly theory (*36*)], then we should be able to track down the parent molecules and substantially narrow down the list of suspected contaminants. Nevertheless, it should be noticed that we only introduced the concept here. The development of a reliable detection method would require a massive tree inclusive of all relevant compounds both in terms of possible pollutants and products expected in the absence of pollution based on the environmental conditions.

Opiates

The search of opiate-based chemical space is an important test case since the family of compounds is highly distinctive with well-defined modular parts. One idea could be to use assembly trees to explore a set of potentially biochemically accessible new structures, as well as deduce what contingent information is present therein, and use them for finding new molecular targets. To do this, we computed the assembly tree of 10 compounds in the family of opiates (Fig. 6): Some of them are found in the opium plant (morphine, codeine, thebaine, and papaverine); some are synthetic opioids (fentanyl, remifentanil, methadone, pethidine, and diamorphine, also known as heroin), and the last one is salvinorin A, which is a κ-opioid receptor agonist (*48*) found in the *Salvia divinorum* plant but might not be properly considered as an opioid. The assembly tree captures some known features of the chemical space associated with these compounds: (i) Morphine, codeine, thebaine, and diamorphine are clustered in one place, with a major structure shared. (ii) Fentanyl and remifentanil are close in the tree, as they share a large substructure. (iii) In contrast, methadone, pethidine, and papaverine are not as closely related, sharing just a relatively small benzyl moiety. (iv) Salvinorin A is distinct from others, as it only connects with other compounds via very small structures.

By computing the assembly tree of these compounds, we obtained not only the shortest but also other longer assembly pathways (another output of our algorithm; see section S4). The key building blocks included in all these pathways, constituting the assembly space of these compounds, are highly related and encode their structural information. Given that the molecules connected by these graphs

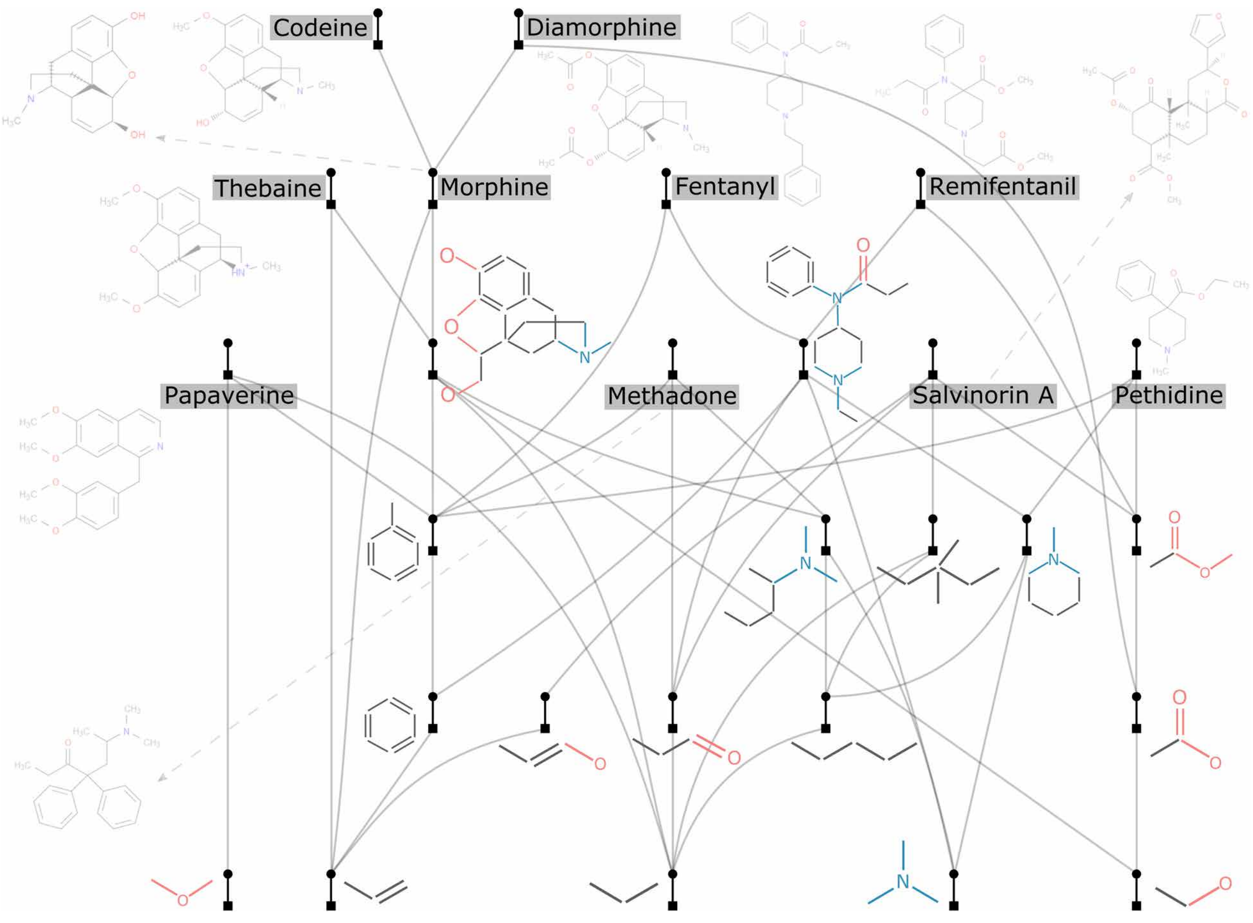

Fig. 6. The assembly tree of nine compounds in the family of opiates and one κ-opioid receptor agonist (salvinorin A). Some of these opiates are natural (morphine, codeine, thebaine, and papaverine), while others are synthetic (fentanyl, remifentanil, methadone, pethidine, and diamorphine, also known as heroin). For a clearer visualization, all opioids are made dimmer than other parts of the tree.

are real and functionally interesting, we think it could be fruitful to see if navigating the trajectories defined by these pathways could lead to the discovery of new potential drug candidates. The idea to explore the space of natural products by fragmenting them is not new and has been tried before (*49*–*51*). For instance, the fragment-based drug discovery (FBDD) is a de novo generation strategy that uses fragments of known bioactive compounds to obtain new drug-like molecules (*52*, *53*). The idea behind FBDD is that functionally comparable molecules share structural similarities; therefore, selecting fragments from molecules would propagate their properties, such as biological activity, to the newly generated compounds (*54*–*56*).

Compared to the size of the comparable chemical space, the assembly space of these compounds is substantially smaller. We used the MC method to compute the pathways, so the size of the assembly space changes along with the number of MC steps. We found that the size of the assembly space is approximately 1500, which was consistent even as we increase the number of MC steps up to the number that is far larger than the number needed to have very short pathways found. In this case, the smallest MA is found to be 105, while the total number of bonds in these 10 compounds is 268 (see details in section S6 for how the size of the assembly space changes with the number of MC steps). This small number (~1500) is not because our approach or our program cannot find more unique structures, but the assembly space itself is intrinsically small since it only includes structures that are shared by at least two compounds, instead of any feasible structure or fragment. For a computationally tractable comparison, we used MOLGEN 5.0 (*57*) to calculate the total number of structures possible using a total of 10 carbon, nitrogen, oxygen, or sulfur species, which amounts to over 10^9 unique possibilities and is much larger than the assembly space here. As morphine has 21 nonhydrogen atoms, the number of possible structures will be much larger than 10^9.

Next, we used the assembly trees for the purpose of de novo molecule generation by reconnecting the elements from the assembly pool. Critically, the products of these "reassembly" are closely related to the parent compounds, both structurally and in terms of functional properties. This means that the reassembly process locally explores the chemical space of the input compounds. To do this, we have implemented a method known as the Reassembler (see section S7 for details). Briefly, it connects the assembly pool elements through the same pattern in which they were disconnected from their parent compound(s). This is effectively the reverse process used to generate the tree in the first place. To prove that generated molecules retain similarity to the parent compound(s), we have generated the assembly pool of known natural opiates [in contrast to the 10 compounds in Fig. 6, here, we used codeine, morphine, noscapine, oripavine,

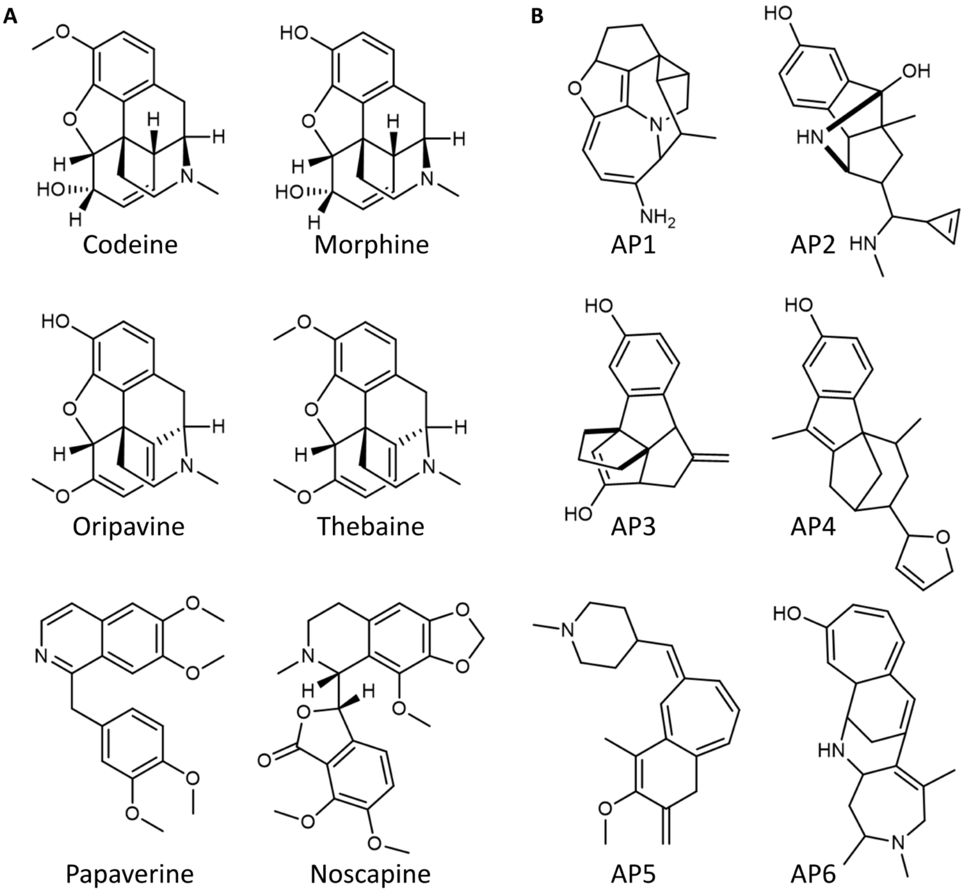

Fig. 7. Comparison between natural opiates and opiate-like molecules generated using Reassembler. (**A**) shows the six opiates used to generate the assembly pools, and (**B**) shows six new opiate-like molecules generated from those assembly pools. See section S7.3 for more detailed information on more new compounds.

papaverine, and thebaine (Fig. 7A) to avoid bias]. We used this assembly pool to generate 1000 opiate-like compounds, and an example set of these molecules is shown in Fig. 7B.

For comparison purposes, we also generated 1000 random compounds only from the bonds present in the opiates (C─C, C═C, C─O, C═O, C─N, and C═N). For consistency, we have limited the molecular weight of all generated molecules and the unsaturation levels in the same range as the parent opiates, i.e., 281 to 368 Da and 9 to 12 degrees of unsaturation. To ensure that the generated molecules were at least chemically plausible, they were passed through two filters: The first filter uses SMARTS (SMILES arbitrary target specification) patterns that are commonly used to detect forbidden structures/structural motifs (*57*), while the other is based on RDKit conformation optimizer (see section S7) (*58*). If any of the forbidden structures were present or no conformation could be found at all, then the molecule was rejected, and another was generated in its place. As shown in Fig. 8A, molecules generated from assembly pools showed significantly higher similarity to opiates than the random compounds (we also compared our result with the molecules generated from arbitrary substructural fragments; see section S8 for details, where our result is still better). Furthermore, they also exhibited similar levels of drug-likeness to the opiates, measured using the “quantitative estimate of drug-likeness (QED)”, as opposed to random molecules which were significantly less drug-like (see Fig. 8B), showing that properties of parent molecules were retained just as well as the structural similarities. While two of the used parent opiates, morphine and oripavine, may look almost identical to the human eye, the seemingly small differences (i.e., morphine cyclohexene ring versus oripavine cyclohexadiene ring with extra methylation on its hydroxyl group) determine significant differences in their properties. Morphine is a common analgesic, while oripavine is not clinically useful because of its toxicity and low therapeutic index. This suggests that, in the chemical space, the distance between these molecules might be greater than the apparent structural similarities alone.

Therefore, a more discrete measure is needed to capture their overall similarity level. In the example of assembly-opitate-1 (AP1) (see Fig. 7B), it has a discrete structure comprising a skeleton that combines the assembly spaces of the opiate-based space surrounding the known opiates, such as morphine or codeine. Thus, it is easy to see the structural similarities between AP1 and morphine as shown by the Tanimoto similarity score ca. 0.24. Furthermore, the QED is 0.72, while the QED of morphine is 0.70, which is notably close. In addition, the log*P* of AP1 is 2.42, while morphine log*P* is 1.20. Nevertheless, the log*P* of morphine’s close relative, oripavine, is 2.12, which is close to our hypothetical AP1. Thus, our hybrid AP1 seems to occupy a position in chemical space intermediate between morphine and oripavine. The compound has similarity to most opiates, including codeine, thebaine, and even noscapine, thereby occupying the intermediate space between all the opiates. This is promising since these molecules could be reasonably considered as

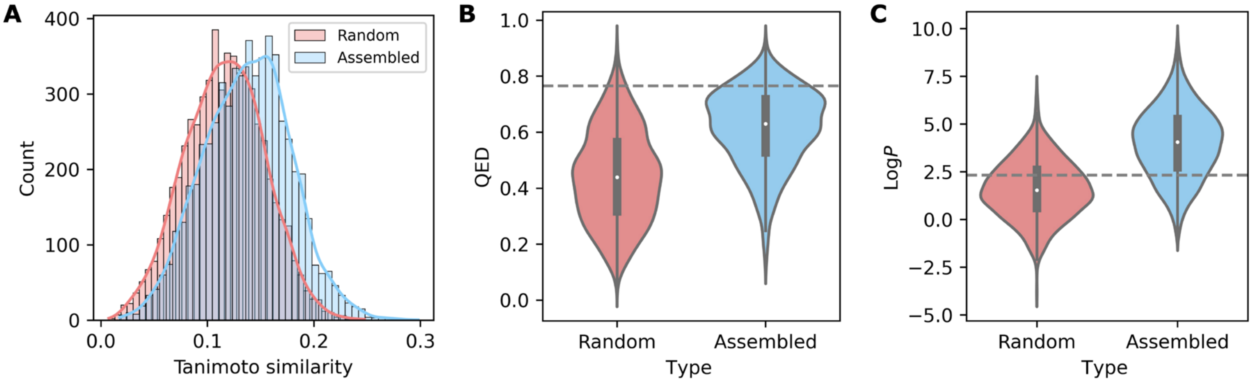

Fig. 8. The comparison of 1000 molecule sets generated from opiate assembly pool (blue) and generated from individual bonds (red). (**A**) According to the Tanimoto similarity measure, products of assembly pools were significantly more similar to the parent molecules (opiates) than randomly generated products. (**B**) QED shows that the assembly products, unlike their random counterparts, showed similar level of drug-likeness to opiates (denoted by gray dotted line). (**C**) On the basis of log*P* estimation, assembly products usually had higher log*P* than opiates (denoted by gray dotted line), while random molecules usually had lower log*P*.

novel synthetic targets to be made (their constrained skeletons are themselves a hard target) and appear to be the first in a line of artificial natural products. A key question is whether it is cost effective to generate practical synthetic approaches to such molecules and to constrain the search of the assembly space around molecules that might be themselves easily made.

Our results demonstrate how the assembly theory can be used to generate compressed representations of chemical space while retaining the relevant chemical and structural information. This means that it is possible to show how assembly spaces can be extended to multiple compounds, and we introduced a notation to effectively represent the key features of such assembly spaces. By developing an MC algorithm to calculate the shortest assembly pathways of a single molecule, we also show how it is possible to generate the assembly tree of any collection of molecules. We demonstrated this methodology in four distinct use cases, prebiotic chemistry, genetics, environmental chemistry, and drug discovery. The assembly tree of biomolecules shows that those vital molecules used in biology represent a compressed subset of the possible compounds, suggesting that they were subjected to evolutionary optimization. By analyzing the assembly space of plasticizers, it was possible to identify structural motifs common to many different pollutants. These motifs can be used as general-purpose signals to identify entire classes of pollutants in complex environmental samples. The analysis of the opioids provides a map to future drug development, by extracting the hierarchical relationships between compounds and identifying key components of possible drug candidates. The analysis of gene sequences demonstrates how assembly spaces can provide a lossless compression of sequences that retains the repeated motifs, demonstrating how complex gene sequences could be reconstructed from minimal genetic inputs. These four different case studies demonstrate the wide applicability of assembly trees as a tool in a diverse set of disciplines. Last, we developed a scheme to take a target set of molecules as templates for silico discovery and explore the enumeration of these targets using random and assembly tree–constrained approaches (Figs. 7 and 8). The evaluation of the properties provides a useful test of how assembly trees can capture information encoded with a chemical network constructed using external constraints from either biochemistry or technology. We hope that by exploring these trees, with information from other sources about what molecules are synthetically feasible, it will be possible to develop new routes to structure expansion that encode the transformations of chemical synthesis.

MATERIALS AND METHODS

The MC algorithms described in this manuscript were implemented in C++ and can be compiled using Visual Studio 2019. This code relied on the InChI standard libraries found at www.inchi-trust.org/. The Reassembler approach to generate new compounds from assembly pools was implemented in Python (with RDKit). Details on the implementation and instructions on how to use the software can be found in the Supplementary Materials.

SUPPLEMENTARY MATERIALS

Supplementary material for this article is available at https://science.org/doi/10.1126/sciadv.abj2465

REFERENCES AND NOTES

1. C. M. Dobson, Chemical space and biology. *Nature* **432**, 824–828 (2004).
2. C. Lipinski, A. Hopkins, Navigating chemical space for biology and medicine. *Nature* **432**, 855–861 (2004).
3. R. S. Bohacek, C. McMartin, W. C. Guida, The art and practice of structure-based drug design: A molecular modeling perspective. *Med. Res. Rev.* **16**, 3–50 (1996).
4. P. Kirkpatrick, C. Ellis, Chemical space. *Nature* **432**, 823–823 (2004).
5. S. Goto, Y. Okuno, M. Hattori, T. Nishioka, M. Kanehisa, LIGAND: Database of chemical compounds and reactions in biological pathways. *Nucleic Acids Res.* **30**, 402–404 (2002).
6. S. A. Sandford, M. Nuevo, P. P. Bera, T. J. Lee, Prebiotic astrochemistry and the formation of molecules of astrobiological interest in interstellar clouds and protostellar disks. *Chem. Rev.* **120**, 4616–4659 (2020).
7. R. J. P. Williams, The natural selection of the chemical elements. *Cell. Mol. Life Sci.* **53**, 816–829 (1997).
8. H. J. Cleaves II, C. Butch, P. B. Burger, J. Goodwin, M. Meringer, One among millions: The chemical space of nucleic acid-like molecules. *J. Chem. Inf. Model.* **59**, 4266–4277 (2019).
9. J. Clardy, C. Walsh, Lessons from natural molecules. *Nature* **432**, 829–837 (2004).
10. M. Frenkel-Pinter, M. Samanta, G. Ashkenasy, L. J. Leman, Prebiotic peptides: Molecular hubs in the origin of life. *Chem. Rev.* **120**, 4707–4765 (2020).
11. Y. I. Wolf, N. V. Grishin, E. V. Koonin, Estimating the number of protein folds and families from complete genome data. *J. Mol. Biol.* **299**, 897–905 (2000).
12. A. M. Boldi, Libraries from natural product-like scaffolds. *Curr. Opin. Chem. Biol.* **8**, 281–286 (2004).
13. R. R. Breaker, Natural and engineered nucleic acids as tools to explore biology. *Nature* **432**, 838–845 (2004).

14. D. Brohm, S. Metzger, A. Bhargava, O. Müller, F. Lieb, H. Waldmann, Natural products are biologically validated starting points in structural space for compound library development: Solid-phase synthesis of dysidiolide-derived phosphatase inhibitors. *Angew. Chem. Int. Ed. Engl.* **41**, 307–311 (2002).
15. B. R. Stockwell, Exploring biology with small organic molecules. *Nature* **432**, 846–854 (2004).
16. J. L. Reymond, The chemical space project. *Acc. Chem. Res.* **48**, 722–730 (2015).
17. L. Ruddigkeit, R. van Deursen, L. C. Blum, J.-L. Reymond, Enumeration of 166 billion organic small molecules in the chemical universe database GDB-17. *J. Chem. Inf. Model.* **52**, 2864–2875 (2012).
18. C. Knox, V. Law, T. Jewison, P. Liu, S. Ly, A. Frolkis, A. Pon, K. Banco, C. Mak, V. Neveu, Y. Djoumbou, R. Eisner, A. C. Guo, D. S. Wishart, DrugBank 3.0: A comprehensive resource for 'omics' research on drugs. *Nucleic Acids Res* **39**, D1035–D1041 (2011).
19. Y. Wang, J. Xiao, T. O. Suzek, J. Zhang, J. Wang, S. H. Bryant, PubChem: A public information system for analyzing bioactivities of small molecules. *Nucleic Acids Res.* **37**, W623–W633 (2009).
20. J.-L. Reymond, L. Ruddigkeit, L. Blum, R. van Deursen, The enumeration of chemical space. *Wiley Interdiscip. Rev Comput. Mol. Sci.* **2**, 717–733 (2012).
21. D. Rogers, M. Hahn, Extended-connectivity fingerprints. *J. Chem. Inf. Model.* **50**, 742–754 (2010).
22. P. Willett, Similarity-based virtual screening using 2D fingerprints. *Drug Discov. Today* **11**, 1046–1053 (2006).
23. J. Rosen, J. Gottfries, S. Muresan, A. Backlund, T. I. Oprea, Novel chemical space exploration via natural products. *J. Med. Chem.* **52**, 1953–1962 (2009).
24. R.-R. Griffiths, J. M. Hernández-Lobato, Constrained Bayesian optimization for automatic chemical design using variational autoencoders. *Chem. Sci.* **11**, 577–586 (2020).
25. I. Y. Kanal, S. G. Owens, J. S. Bechtel, G. R. Hutchison, Efficient computational screening of organic polymer photovoltaics. *J. Phys. Chem. Lett.* **4**, 1613–1623 (2013).
26. N. M. O'Boyle, C. M. Campbell, G. R. Hutchison, Computational design and selection of optimal organic photovoltaic materials. *J. Phys. Chem. C* **115**, 16200–16210 (2011).
27. C. Rupakheti, A. Virshup, W. Yang, D. N. Beratan, Strategy to discover diverse optimal molecules in the small molecule universe. *J. Chem. Inf. Model.* **55**, 529–537 (2015).
28. A. M. Virshup, J. Contreras-Garcia, P. Wipf, W. Yang, D. N. Beratan, Stochastic voyages into uncharted chemical space produce a representative library of all possible drug-like compounds. *J. Am. Chem. Soc.* **135**, 7296–7303 (2013).
29. G. M. Keseru, T. Soos, C. O. Kappe, Anthropogenic reaction parameters—The missing link between chemical intuition and the available chemical space. *Chem. Soc. Rev.* **43**, 5387–5399 (2014).
30. M. Awale, R. Visini, D. Probst, J. Arus-Pous, J. L. Reymond, Chemical space: Big data challenge for molecular diversity. *Chimia (Aarau)* **71**, 661–666 (2017).
31. T. Hoffmann, M. Gastreich, The next level in chemical space navigation: Going far beyond enumerable compound libraries. *Drug Discov. Today* **24**, 1148–1156 (2019).
32. R. Gomez-Bombarelli, J. N. Wei, D. Duvenaud, J. M. Hernández-Lobato, B. Sánchez-Lengeling, D. Sheberla, J. Aguilera-Iparraguirre, T. D. Hirzel, R. P. Adams, A. Aspuru-Guzik, Automatic chemical design using a data-driven continuous representation of molecules. *ACS Cent Sci* **4**, 268–276 (2018).
33. D. Duvenaud, D. Maclauriny, J. Aguilera-Iparraguirre, R. Gomez-Bombarelli, T. Hirzel, A. Aspuru-Guzik, R. P. Adams, paper presented at the Proceedings of the 28th International Conference on Neural Information Processing Systems—Volume 2, Montreal, Canada, 2015.
34. M. Ilardo, M. Meringer, S. Freeland, B. Rasulev, H. J. Cleaves II, Extraordinarily adaptive properties of the genetically encoded amino acids. *Sci. Rep.* **5**, 9414 (2015).
35. S. M. Marshall, A. R. G. Murray, L. Cronin, A probabilistic framework for identifying biosignatures using Pathway Complexity. *Phil. Trans. A Math Phys. Eng. Sci.* **375**, 20160342 (2017).
36. S. M. Marshall, C. Mathis, E. Carrick, G. Keenan, G. J. T. Cooper, H. Graham, M. Craven, P. S. Gromski, D. G. Moore, S. I. Walker, L. Cronin, Identifying molecules as biosignatures with assembly theory and mass spectrometry. *Nat. Commun.* **12**, 3033 (2021).
37. D. L. Nelson, A. L. Lehninger, M. M. Cox, *Lehninger Principles of Biochemistry* (W. H. Freeman, 2008).
38. L. Nick, *Life Ascending: The Ten Great Inventions of Evolution*. (W. W. Norton & Co., 2009).
39. E. Meléndez-Hevia, T. G. Waddell, M. Cascante, The puzzle of the Krebs citric acid cycle: Assembling the pieces of chemically feasible reactions, and opportunism in the design of metabolic pathways during evolution. *J. Mol. Evol.* **43**, 293–303 (1996).
40. Y. Furukawa, Y. Chikaraishi, N. Ohkouchi, N. O. Ogawa, D. P. Glavin, J. P. Dworkin, C. Abe, T. Nakamura, Extraterrestrial ribose and other sugars in primitive meteorites. *Proc. Natl. Acad. Sci. U.S.A.* **116**, 24440–24445 (2019).
41. D. L. Tumbula, Q. Teng, M. G. Bartlett, W. B. Whitman, Ribose biosynthesis and evidence for an alternative first step in the common aromatic amino acid pathway in *Methanococcus maripaludis*. *J. Bacteriol.* **179**, 6010–6013 (1997).
42. S.-J. Lin, L. Guarente, Nicotinamide adenine dinucleotide, a metabolic regulator of transcription, longevity and disease. *Curr. Opin. Cell Biol.* **15**, 241–246 (2003).
43. A. D. Haimovich, P. Muir, F. J. Isaacs, Genomes by design. *Nat. Rev. Genet.* **16**, 501–516 (2015).
44. C. E. Shannon, A mathematical theory of communication. *Bell Syst. Tech. J.* **27**, 379–423 (1948).
45. R. M. Gray, *Entropy and Information Theory* (Springer, 2011).
46. D. F. Cadogan, C. J. Howick, "Plasticizers" in *Ullmann's Encyclopedia of Industrial Chemistry* (Wiley-VCH, Weinheim, 2012), pp. 599-618.
47. R. U. Halden, Plastics and health risks. *Annu. Rev. Public Health* **31**, 179–194 (2010).
48. B. L. Roth, K. Baner, R. Westkaemper, D. Siebert, K. C. Rice, S. Steinberg, P. Ernsberger, R. B. Rothman, Salvinorin A: A potent naturally occurring nonnitrogenous κ opioid selective agonist. *Proc. Natl. Acad. Sci. U.S.A.* **99**, 11934–11939 (2002).
49. G. Karageorgis, D. J. Foley, L. Laraia, H. Waldmann, Principle and design of pseudo-natural products. *Nat. Chem.* **12**, 227–235 (2020).
50. B. Over, S. Wetzel, C. Grütter, Y. Nakai, S. Renner, D. Rauh, H. Waldmann, Natural-product-derived fragments for fragment-based ligand discovery. *Nat. Chem.* **5**, 21–28 (2013).
51. H. Prescher, G. Koch, T. Schuhmann, P. Ertl, A. Bussenault, M. Glick, I. Dix, F. Petersen, D. E. Lizos, Construction of a 3D-shaped, natural product like fragment library by fragmentation and diversification of natural products. *Bioorg. Med. Chem.* **25**, 921–925 (2017).
52. D. A. Erlanson, R. S. McDowell, T. O'Brien, Fragment-based drug discovery. *J. Med. Chem.* **47**, 3463–3482 (2004).
53. F. W. Goldberg, J. G. Kettle, T. Kogej, M. W. D. Perry, N. P. Tomkinson, Designing novel building blocks is an overlooked strategy to improve compound quality. *Drug Discov. Today* **20**, 11–17 (2015).
54. P. J. Hajduk, J. Greer, A decade of fragment-based drug design: Strategic advances and lessons learned. *Nat. Rev. Drug Discov.* **6**, 211–219 (2007).
55. H. Vu, L. Pedro, T. Mak, B. McCormick, J. Rowley, M. Liu, A. D. Capua, B. Williams-Noonan, N. B. Pham, R. Pouwer, B. Nguyen, K. T. Andrews, T. Skinner-Adams, J. Kim, W. G. J. Hol, R. Hui, G. J. Crowther, W. C. Van Voorhis, R. J. Quinn, Fragment-based screening of a natural product library against 62 potential malaria drug targets employing native mass spectrometry. *ACS Infect. Dis.* **4**, 431–444 (2018).
56. M. E. Welsch, S. A. Snyder, B. R. Stockwell, Privileged scaffolds for library design and drug discovery. *Curr. Opin. Chem. Biol.* **14**, 347–361 (2010).
57. R. Gugisch, A. Kerber, A. Kohnert, R. Laue, M. Meringer, C. Rücker, A. Wassermann, in *Advances in Mathematical Chemistry and Applications,* S. C. Basak, G. Restrepo, J. L. Villaveces, Eds. (Bentham Science Publishers, 2015), pp. 113–138.
58. S. Riniker, G. A. Landrum, Better informed distance geometry: Using what we know to improve conformation generation. *J. Chem. Inf. Model.* **55**, 2562–2574 (2015).

Acknowledgments: We acknowledge A. Murray for helpful discussions and D. Moore and H. Mehr for helpful discussions and input on the algorithmic implementations. **Funding:** We acknowledge financial support from the John Templeton Foundation (grant 60025), EPSRC (grant nos. EP/L023652/1, EP/R01308X/1, EP/J015156/1, and EP/P00153X/1), the Breakthrough Prize Foundation and NASA (Agnostic Biosignatures award no. 80NSSC18K1140), MINECO (project CTQ2017-87392-P), and ERC (project 670467 SMART-POM). **Author contributions:** L.C. conceived the theory and the hypothesis, designed the project, and coordinated the efforts of the research team. Y.L. developed the algorithmic implementation of assembly theory, wrote the software to calculate assembly numbers and assembly spaces with help from S.M.M. and C.M., and developed the scheme to use the Reassembler method to generate new molecules. M.D.B. designed and developed the Reassembler method. L.W. developed and performed the property and similarity-based analyses of the reassembled opiate molecules. L.C., Y.L., C.M., and M.D.B. co-wrote the manuscript, with input from all authors. **Competing interests:** The authors declare that they have no competing interests. **Data and materials availability:** All data needed to evaluate the conclusions in the paper are present in the paper and/or the Supplementary Materials. The Supplementary Materials is linked to the online version of the paper, and the software code is available on a code repository: https://github.com/croningp/Paper-AssemblyTreeOfLife.

Submitted 29 April 2021
Accepted 3 August 2021
Published 24 September 2021
10.1126/sciadv.abj2465

Citation: Y. Liu, C. Mathis, M. D. Bajczyk, S. M. Marshall, L. Wilbraham, L. Cronin, Exploring and mapping chemical space with molecular assembly trees. *Sci. Adv.* **7**, eabj2465 (2021).
