# Supplementary material for: Exploring and mapping chemical space with molecular assembly trees
Source: Sci Adv. 2021 Sep 24;7(39):eabj2465. doi: 10.1126/sciadv.abj2465 (PMC8462901; doi:10.1126/sciadv.abj2465)
Supplement: Supplementary file 2 — Sections S1 to S8 Figs. S1 to S17 Table S1 [file sciadv.abj2465_sm.pdf]

Science Advances
AAAS

Supplementary Materials for

Exploring and mapping chemical space with molecular assembly trees

Yu Liu, Cole Mathis, Michał Dariusz Bajczyk, Stuart Marshall, Liam Wilbraham, Leroy Cronin*

*Corresponding author. Email: lee.cronin@glasgow.ac.uk

Published 24 September 2021, *Sci. Adv.* **7**, eabj2465 (2021)
DOI: 10.1126/sciadv.abj2465

The PDF file includes:

Sections S1 to S8
Figs. S1 to S17
Table S1

Other Supplementary Material for this manuscript includes the following:

Data file S1

1 Glossary for Molecular Assembly Theory

- *Molecular assembly theory*: The name of the theory in general.
- *(Assembly) building blocks*: The units that are used to construct other units (e.g., in our molecular case, they are either chemical bonds, or chemical fragments, structures, molecules).
- The *(Assembly) pool*: The set of all assembly building blocks.
- One *(assembly) step*: It is defined as the three sequential operations (also mentioned in the main text): (1) Take two assembly building blocks from the assembly pool; (2) Join the two together in a way based on the particular system in question; (3) Put the new composite building block back into the assembly pool, and it will be considered as an assembly building block hereafter.
- One *(assembly) pathway* of a molecule: One sequence of assembly steps that successfully construct the target molecule.
- The *(assembly) index* of one assembly pathway: The number of assembly steps of the assembly pathway.
- The *shortest (assembly) pathways* of a molecule: The assembly pathways of the molecule that have the minimum number of assembly steps. Note that there could be more than one shortest assembly pathways.
- The *molecular assembly number* (MA) of a molecule: The assembly index of the shortest assembly pathway(s) of the molecule.
- The *assembly space of one molecule*: It is a term used in a more general sense, to refer to all of the assembly building blocks and the associated relationships included in many or all assembly pathways of the molecule.

- *Key (assembly) building blocks*: The assembly building blocks that are used more than once in the assembly pathway. They describe the hierarchical relationships among the chemical fragments / structures / molecules.
- *Key (assembly) steps*: The corresponding assembly steps that make the key assembly building blocks.
- The *key-step representation* of an assembly pathway: It only displays key assembly building blocks in a hierarchical manner (see Fig. 1b in the main text).
- The *multiset representation* of an assembly pathway: It only displays key assembly building blocks, together with their multiplicities (namely, the number of times they occur), in the format of a multiset (see Fig. 1d in the main text).
- The *(Molecular) assembly tree* of a group of molecules: It is defined as the shortest assembly pathways to make this group of molecules altogether. It can either be drawn in the key-step representation or written in the multiset representation.
- *MA of an assembly tree* refers to this assembly tree's assembly index.

2 Calculate the assembly index of an assembly pathway from its multiset representation

Based on the multiset representation of an assembly pathway, its assembly index can be readily worked out, taking the assembly pathway of adenine shown in Fig. 1 in the main text as an example. The logic is: (i) The longest assembly pathway for a molecule is to join one bond for each step. As adenine has 11 bonds, we need 11-1=10 steps. (ii) By reusing one building blocks shown in the multiset representation, x number of steps is saved, where x is equal to the number of bonds of this building block minus 1. For building block [2] (referring to Fig. 1 in the main text), it saves 3-1=2 steps, while for building block [1], it saves 2-1=1 step. Finally, we obtain

the index of this pathway, equal to 10-2-1=7, which is consistent with the result as we directly count the number of steps according to the definition.

3 Monte Carlo algorithm: for a single molecule

3.1 The overview of the Monte Carlo algorithm

Here we will introduce a Monte Carlo algorithm to compute the shortest assembly pathways of a molecule. A Monte Carlo algorithm was developed because the computational power required to solve the problem exactly is expected to be significant for large molecules. Notice that, this problem could be considered as a combination of two difficult classic problems: (i) the shortest *addition chain* problem ((*36*), Downey *SIAM Journal on Computing* 638-646, 1981; Knuth *The Art of Computer Programming Vol. 2* 461-485, 1997) and (ii) the *subgraph isomorphism problem* which has been proven to be NP-complete (Cook *Proceedings of the third annual ACM symposium on Theory of computing* 151-158 ,1971; Leeuwen *Handbook of Theoretical Computer Science*, 1998). Thus, it is at least as hard as NP-complete (see SI section 3.2 for details). Since obtaining precise results is computationally expensive, we adopted the Monte Carlo approach (Kroese *WIREs Computational Statistics* 386-392, 2014) that allows us to efficiently calculate approximate results that are exact in the limit of infinite computational time.

First, we need to calculate a distribution that shows how many times a molecular structure or fragment is duplicated in the original molecule. Figure S1 shows a part of the distribution for adenine, and three exemplified fragments. To obtain the full distribution, we would need to fragment the molecule in every possible way, however as the size of the molecule increases, the number of all possible fragmenting schemes grows rapidly, leading to a combinatorial

explosion. So, in this step, we adopted the Monte Carlo approach where we (i) randomly fragment the molecule many times and count how many times each fragment appears, and then (ii) convert these counts into the desired distribution, based on statistical techniques (see SI section 3.3 for details). The underlying logic is that if a fragment duplicates many times in the original molecule, there would be many ways to fragment the original molecule so that this fragment appears, resulting in a large value in the distribution.

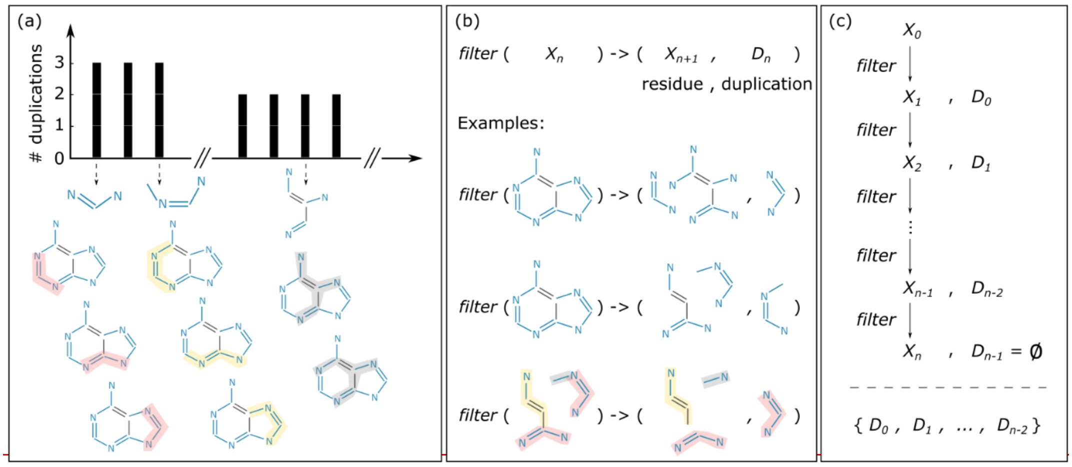

Fig. S1. The general scheme of the Monte Carlo algorithm to compute the shortest assembly pathways of a molecule. (a) Schematic diagram of the fragments distribution that is obtained in the first step of the Monte Carlo algorithm. As we see, the first fragment has y-value 3, so it duplicates 3 times in adenine. The three adenines with colored patches under this fragment show where to find these three duplications. (b) The expected results of the operator *filter()*. Take the last panel as an example. In this case, this set of fragments is covered up by the drawn fragments highlighted in colors. The two red fragments are identical (thus duplicated), so one of them is filtered out as Dn, and all the left-over fragments constitute the residue. (c) The scheme of the overall process to obtain one assembly pathway, shown at the bottom. For each time we run this process, we obtain one pathway. Run this process for a certain number of times (a predefined parameter), and the shortest assembly pathways among them are the final results.

Second, we introduce an operator *filter*(X_0) where X_0 is a molecule, a fragment, or a set of fragments, which is used to find one duplicated fragment (if any) in X_0 and filter that out. This

operator returns two results simultaneously: one is the duplicated fragment D_0 (if there is none, returns empty ∅) while the other is all the left-over parts of X_0 after D_0 is excluded which we call as the residue, X_1. So, we can denote *filter*(X_0) -> (X_1, D_0) (see Fig. S1b). The operator *filter*(X_0) searches for duplications by first randomly drawing fragments from the distribution (obtained in the first step) to cover parts of X_0 until X_0 is completely covered by fragments, and then filtering the duplicated fragment (D_0), leaving us the residue X_1 (if the fragments tried to cover up X_0 have no duplications, the operator will start over until there is one). Note that *filter()* involves a random process (i.e., randomly drawing fragments from the distribution), so for each run it may generate different results.

Now, we apply *filter()* to the original molecule X_0, and get (X_1, D_0). Next, we apply *filter()* to the newly calculated residue X_1 and get (X_2, D_1), and further apply *filter()* to X_2. Keep applying *filter()* to the newly calculated residue again and again until no duplication can be found, i.e., *filter()* returns an empty duplication ∅. Then, all the duplications obtained along the process constitute one assembly pathway (see Fig. S1c), whose assembly index can be easily calculated. So, for each time we run this process, we obtain one pathway. Finally, we repeat this process for a certain number of steps (a predefined parameter), the shortest assembly pathways obtained are the final results. The assembly index of the shortest pathway therefore determines the MA for the molecular target. See SI section 3.4 for the flowchart and details, and section 3.6 for the calculated results for several exemplified molecules.

3.2 Why finding the shortest assembly pathway is at least as hard as NP-complete?

As first introduced by Knuth (Knuth *The Art of Computer Programming Vol. 2* 461-485, 1997), an *addition chain for n* is defined to be a sequence of integers $1 = a_0, a_1, a_2, \ldots, a_r = n$ with the

property that $a_i = a_j + a_k$, for some $k \leq j < i$, for all $i = 1, 2, \ldots, r$. So, to find the shortest addition chain of an integer is a very special and simplified version of our question to find the shortest assembly pathway of a molecule (*36*), by simply considering an integer as a linear molecule made of carbons. As we know, to calculate the shortest addition chain for a given integer is a very difficult problem and no known algorithm can be guaranteed to finish in a reasonable time (although whether this problem is NP-complete or NP-hard has not been proven). On the other hand, a generalized version of the shortest addition chain problem – to find an addition chain that simultaneously forms each of a sequence of integers – has been proven to be NP-complete (Downey *SIAM Journal on Computing* 638-646, 1981). This generalized version is exactly a largely simplified version of the question to compute the *molecular assembly tree* for a group of molecules (this concept is introduced in Section "Molecular Assembly Trees" of the main text).

In fact, to find the shortest assembly pathway for a single molecule is the combination of two problems: the first is to find the shortest addition chain of an integer, and the second is to determine if one graph (namely the graph representations of molecules, chemical structures and fragments) contains a subgraph that is isomorphic to another graph, which is an example of the *subgraph isomorphism problem,* which has been proven to be NP-complete (Cook *Proceedings of the third annual ACM symposium on Theory of computing* 151-158 ,1971). Besides, to compute the assembly tree for a group of molecules is the combination of (i) to find an addition chain that simultaneously forms each of a sequence of integers and (ii) the subgraph isomorphism problem. Therefore, either to find the shortest assembly pathway for a single molecule or to compute the assembly tree for a group of molecules is at least as hard as an NP-complete problem. See Table S1 where we have summarized the computational complexity of these problems.

Table S1. Summary of the computational complexity of the problems to find the shortest assembly pathway and to compute the assembly tree.

Problems	To find the shortest assembly pathway for a single molecule	=	To find the shortest addition chain of an integer	+	Subgraph isomorphism problem
Computational complexity	At least as hard as NP-complete		Hard, but not been proven to be NP-complete or NP-hard		NP-complete
Problems	To compute the assembly tree for a group of molecules	=	To find an addition chain that simultaneously forms each of a sequence of integers	+	Subgraph isomorphism problem
Computational complexity	At least as hard as NP-complete		NP-complete		NP-complete

3.3 How to obtain the fragments distribution?

(a)

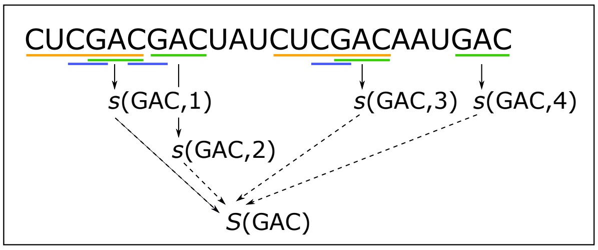

(b)

C	7.99745	0.386026	0.0308722
A	7.00067	0.434606	0.0304253
U	4.99414	0.526427	0.0262905
G	4.01473	0.593491	0.0238271
AC	4.01132	0.80753	0.0323927
GA	3.9976	0.859352	0.0343534
GAC	3.95732	1.15654	0.0457679
UC	3.00258	0.99669	0.0299264
CU	2.98637	0.917381	0.0273964
CG	2.9772	1.00106	0.0298036
CGAC	2.97365	2.02611	0.0602493
CGA	2.96631	1.42916	0.0423931
CUCG	2.04379	2.11977	0.0433238
CUC	2.02868	1.50112	0.030453
CUCGAC	2.02287	4.27189	0.0864146
UCG	2.0009	1.74432	0.0349022
UCGA	2.00089	2.47291	0.0494801
AU	1.97978	1.23388	0.024428
CUCGA	1.96403	3.05657	0.0600318
UCGAC	1.94161	3.55429	0.0690105

Fig. S2. Illustration and outcome of the algorithm to obtain the fragments distribution. (a) The exemplified original string. (b) The output of the algorithm. The first column shows how many substance of each fragment are contained in the original string. Because this algorithm is statistical, all the values are not

integers, but close to. The third column shows the measurement error (standard deviation). The second column equal to the third divides the first column, in percentage. All fragments whose measurement error is larger than 5% are dropped.

This part of the algorithm is to calculate a distribution that shows how many times a molecular structure or fragment is duplicated in the original molecule. For convenience, here we use a string (Fig. S2a) as an example to illustrate instead of a molecule, but the same principle applies to molecules. Now, the question is to find how many instances of each fragment (namely, substring) are contained in the original string. We see that the fragment CUCGAC appears twice, GAC appears four times, CG appears three times, etc. Ultimately, the algorithm should be able to tell us those information.

More precisely, the algorithm should tell us the value *w(S)* that describes how many instances of each fragment *S* are contained in the original string. This algorithm is conceptually simple, and has two main steps:

1. Randomly fragment the original string for many times, and count how many times each fragment appears, thus a histogram obtained.
2. Weight the counts more for longer fragments. Generally speaking, the weighting scheme is: "the weighted counts of a fragment *S*" is proportional to "(the actual counts of *S*) × (2 to the power of the length of *S*)".
3. Normalized the weighted counts, and then we obtain *w(S)*.

The output of this algorithm applied to the exemplified string is shown in Fig. S1b. The details of why this can work are shown in the following.

- Notation

First of all, we denote a specific fragment as s (lowercase). Note that if we want to distinguish two fragments that are identical but in different positions, we will use the lowercase s. For example, in Fig. S2a, the fragment GAC that consists of the 4th, 5th and 6th letter is denoted as s(GAC,1); while we denote another GAC fragment that consists of the 7th, 8th and 9th letter as s(GAC,2). On the other hand, if we do not want to distinguish fragments that are identical but in different positions, we will use S (uppercase). As shown in Fig. S2a, all fragments GAC are denoted as S(GAC). Therefore, the ultimate goal of this algorithm is to find $w(S)$, rather than $w(s)$. With that being clear, we can proceed.

Theoretically, we have

n: the number of links of the original string (i.e., the number of letters minus 1);

m: the number of links of the fragment in question;

$N = 2^n - 1$: the total number of unique fragmenting events;

M_a and M_b: in these N unique events, for any fragment s on the edge, it will appear for $M^a = 2^{n-m-1}$ times, while for any fragment s in the middle, it will appear for $M_b = 2^{n-m-2}$ times;

Π: the total number of fragments appeared in these N unique events, that is

$$\Pi = 2\binom{n}{1} + 3\binom{n}{2} + 4\binom{n}{3} + \cdots + (n+1)\binom{n}{n} = (n+2)\cdot 2^{n-1} - 1$$

Empirically, we have

$\widehat{N}$: the number of fragmenting events in the experiments (referred to as fragmenting trails);

$\widehat{\Pi}$: the total number of fragments appeared in these $\widehat{N}$ empirical trails.

So, in these $\widehat{N}$ empirical trails, the number of times that one fragment s appears is denoted as $\hat{h}_x(s)$. If s is on the edge, it is denoted as $\hat{h}_a(s)$; if s is in the middle, it is denoted as $\hat{h}_b(s)$.

Therefore, based on *Wilson score interval* in statistics, the probability of the fragment *s* appears is (mean ± standard deviation):

$$\hat{p}_x(s) = \frac{\hat{h}_x(s) + \frac{z^2}{2}}{\hat{\Pi} + z^2} \pm \frac{z}{\hat{\Pi} + z^2}\sqrt{\frac{\hat{h}_x(s)(\hat{\Pi} - \hat{h}_x(s))}{\hat{\Pi}} + \frac{z^2}{4}}$$

where x is replaced by a if the fragment s is on the edge, and x is replaced by b if s is in the middle; z is the *probit function* (when the confidence level is 95%, then z = 1.96). One step further, the probability of the fragment S appears on the edge is:

$$\hat{p}_a(S) \doteq \frac{\hat{H}_a(S) + \frac{z^2}{2}}{\hat{\Pi} + z^2} \pm \frac{z}{\hat{\Pi} + z^2}\sqrt{\frac{\hat{H}_a(S)(\hat{\Pi} - \hat{H}_a(S))}{\hat{\Pi}} + \frac{z^2}{4}} \quad \text{(S3.1)}$$

where $\hat{H}_a(S)$ is the total number of times that S appears on the edge. The probability of the fragment S appears in the middle is:

$$\hat{p}_b(S) \doteq \frac{\hat{H}_b(S) + \frac{z^2}{2}}{\hat{\Pi} + z^2} \pm \frac{z}{\hat{\Pi} + z^2}\sqrt{\frac{\hat{H}_b(S)(\hat{\Pi} - \hat{H}_b(S))}{\hat{\Pi}} + \frac{z^2}{4}} \quad \text{(S3.2)}$$

where $\hat{H}_b(S)$ is the total number of times that S appears in the middle.

- Now we can show how to calculate *w(S)* based on the empirical data

Referring to the string in Fig. S2a, the fragment GAC has 4 instances. In the N unique fragmenting events, the fragment s(GAC,1) will appear M_b = 2^{23-2-2} times, denoted as $h_b(s(\text{GAC},1))$. The same for s(GAC,2) and s(GAC,3). On the other hand, in the N unique fragmenting events, the fragment s(GAC,4) will appear M^a = 2^{23-2-1} times, denoted as $h_a(s(\text{GAC},4))$. Eventually, we can use these counts h_x to achieve the answer 4, i.e.,

$$\begin{aligned} w(S = GAC) &= \frac{h_a(s(GAC,4))}{M_a} + \frac{h_b(s(GAC,1))}{M_b} + \frac{h_b(s(GAC,2))}{M_b} + \frac{h_b(s(GAC,3))}{M_b} \\ &= \frac{h_a(s(GAC,4))}{M_a} + \frac{h_b(s(GAC,1)) + h_b(s(GAC,2)) + h_b(s(GAC,3))}{M_b} \end{aligned}$$

In general, we have

$$w(S) = \frac{\Sigma_i h_a(s_i)}{M_a} + \frac{\Sigma_j h_b(s_j)}{M_b}$$
$$= \frac{H_a(S)}{M_a} + \frac{H_b(S)}{M_b}$$

where $H_a(S)$ is the total number of times that S appears on the edge, and $H_b(S)$ is for S in the middle. If we substitute the empirical values, we obtain the measured $w(S)$, i.e., $\hat{w}(S)$. In order to evaluate the empirical error, we use Eq. (S3.1) and (S3.2) to calculate $H_a(S)$ and $H_b(S)$, rather than directly using $\hat{H}_a(S)$ and $\hat{H}_b(S)$. Therefore, we have

$$\hat{w}(S) = \frac{\Pi \cdot \hat{p}_a(S)}{M_a} + \frac{\Pi \cdot \hat{p}_b(S)}{M_b}$$

Equivalently, the mean is

$$E[\hat{w}(S)] = \frac{\Pi}{M_a} \frac{\hat{H}_a(S) + \frac{z^2}{2}}{\hat{\Pi} + z^2} + \frac{\Pi}{M_b} \frac{\hat{H}_b(S) + \frac{z^2}{2}}{\hat{\Pi} + z^2} \tag{S3.3}$$

and the standard deviation is

$$\sigma[\hat{w}(S)] = \sqrt{\left(\frac{\Pi}{M_a} \frac{z}{\hat{\Pi} + z^2} \sqrt{\frac{\hat{H}_a(S)(\hat{\Pi} - \hat{H}_a(S))}{\hat{\Pi}} + \frac{z^2}{4}}\right)^2 + \left(\frac{\Pi}{M_b} \frac{z}{\hat{\Pi} + z^2} \sqrt{\frac{\hat{H}_b(S)(\hat{\Pi} - \hat{H}_b(S))}{\hat{\Pi}} + \frac{z^2}{4}}\right)^2}$$

To summary, by randomly fragmenting the original string many times, we can get the empirical $\hat{H}_a(S)$, $\hat{H}_b(S)$ and $\hat{\Pi}$, and we can then calculate $E[\hat{w}(S)]$ based on Eq. (S3.3), which is the empirical value of $w(S)$.

3.4 Algorithm description and flowchart

Monte Carlo Step 1:

1.1. Read mol-file to construct a "molecule" object (*MOL_BOND* class), which stores all of the atoms and bonds, also giving each atom an index and each bond (connecting two atoms) an index.

1.2. Find all atoms that have more than one bond linked to it, and make a list recording them.

1.3. For each atom in the list, randomly choose one possible scheme to "split the bonds". For

example, this atom has bond 1, 3 and 6 linked to it; so in total it has five ways to split the bond: no split (136), three ways to split into two (1,36), (3,16) and (6,13), split into three (1,3,6). In general, there is a mathematical formula to generate all of the possible splitting.

1.4. Commit the split, meaning: add atoms to the split end of the bond. For example, if you choose to cut into (1,36), then add an atom to the split end of bond 1; for bond 3 and 6, we don't need to add atoms since we can just use the original end-atom.

1.5. After go through all atoms that have more than 1 bond linked to it, we obtain a molecule with bonds cut (still a *MOL_BOND* class object). Now we can use depth-first-search algorithm to work out all the disconnected parts. Each disconnected part is a fragment of this molecule. Then we obtain a list of fragments.

1.6. For each fragment, we know which bonds it consists of, and we can also calculate the number M (referring to SI section 3.3 for details about M). All of these gives *hist_item* (the information of one item of the overall fragment histogram).

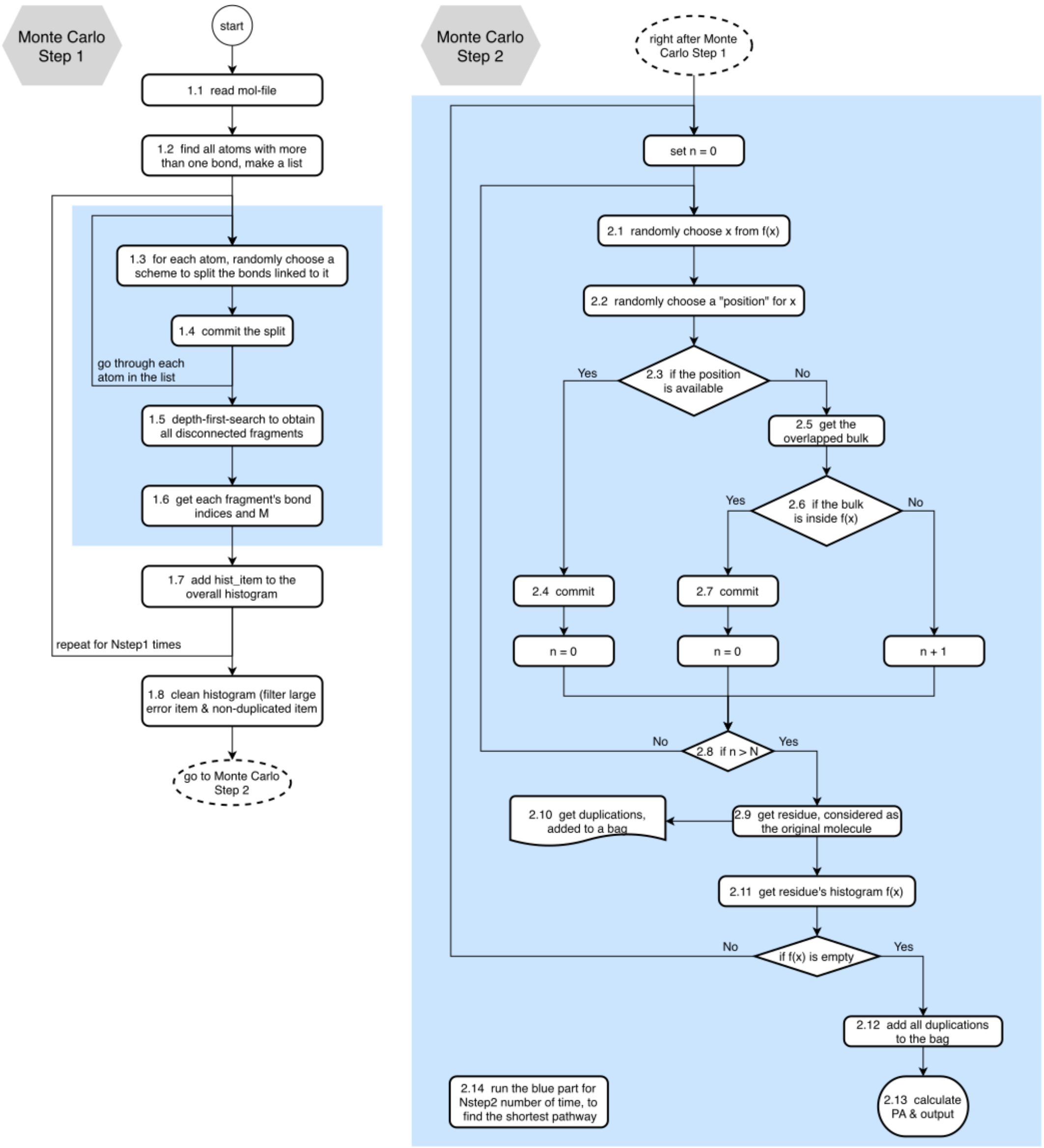

Fig. S3. The flowchart of the Monte Carlo algorithm to compute the shortest assembly pathways for a single molecule. The algorithm is mainly divided into two steps.

As 1.3 - 1.6 (the blue part in the flowchart Fig. S3) are the main processes in Step 1, we summarize them further here: the outcome of 1.3 - 1.6 is to obtain *hist_item* that will be used in 1.7 to construct the distribution of fragments of this molecule (namely, a histogram). *hist_item* is a list of tuples each of which stores the complete information of a fragment (so this list of tuples represents all of the fragments that constitute this molecule). Each tuple consists of three things: InChI string of this fragment, the bond indices that constitute this fragment

(called "fragment identifier"), a number (denoted as M) associated with this fragment that will be used to weight the counts to get the final histogram. Refer to SI section 3.3 to see how M is calculated and used to weight.

1.7. Add *hist_item* to the overall histogram, where the x-axis is the InChI of a particular structure and y-axis is the count of that structure. A map is also constructed which associates a list of fragment identifiers and corresponding M values with each InChI string. That is, the key of this map stores InChI strings; and under any InChI, there is a list of fragment identifiers (that all have the same InChI) and the corresponding M.

After repeating 1.3 - 1.7 for *Nstep1* (a predefined parameter) number of times, we get the overall histogram. The y-axis is the count of how many times a fragment appears in *Nstep1* random cuttings. For example, we are looking at this molecule C-N-C-O-C-N-C; and the fragment C-N-C appears 1 million times in all these cuttings, then y-axis for C-N-C is 1 million. But 1 million is not what we want. Instead, we want to know how many times a fragment has been duplicated in this molecule. In this case, it is 2. So we need to convert the counts (1 million) to number of duplications (two). This conversion can be done by using the counts and the number M (see details in SI section 3.3), which gives the statistical measurement of how many times a fragment is duplicated, mean ± error.

1.8. Clean up the histogram. We first delete the items that have the error > *Hist_Err_Threshold* (a predefined parameter, 5% by default). Then we round the means to integers. Finally, we delete the items whose corresponding y-axis values are 1 (i.e., it only appears once in this molecule).

Monte Carlo Step 1 is now complete. In the end, we obtain a histogram where x-axis is the InChI string of fragment and y-axis is how many times it duplicates in this molecule (with 100%-5% = 95% confidence by default).

Monte Carlo Step 2:

2.1. Denote the histogram obtain from step 1 as *f(x)* where *x* represent a fragment. Create a list *S* recording all the bond indices of the original molecule, e.g., (1,2,3,4,5,6,7,8,9,10,11,12), to represent which bonds have been taken (now it means all bonds are available). Randomly choose a fragment *x* based on the histogram *f(x)*, i.e., the relative y-value represents the probability that a certain *x* is chosen.

2.2. Randomly choose a “position” for *x*. Here “position” means the “fragment identifier”. Recall that in step 1 we have obtained a map linking fragment’s InChI string with a list of fragment identifiers (i.e., the bond indices that constitute the corresponding fragment). For example, if this chosen fragment has 3 duplications: one is (1,2,9), one is (3,5,6) and one is (7,8,12); then here we randomly chose one, say, (3,5,6).

2.3. Check if this position is available, that is, check if each element in (3,5,6) is in the list *S*. Initially, *S* = (1,2,3,4,5,6,7,8,9,10,11,12), so the answer would be yes. But if *S* = (1,5,7,8,9,12), then the answer would be no.

2.4. Commit, so we delete 3, 5 and 6 from the list *S*, means that we consider this fragment has been taken.

2.5. If the position is not available, then it means *x* must be overlapped with one or more already-taken fragments. In the flowchart, get the overlapped bulk means figuring out these already-taken fragments, say, (y_1, y_2, y_3, …), and make a new fragment that consists of *x* and y_1, y_2, y_3…. We denote this big new fragment as bulk *x*’.

2.6. Check if *x*’ is contained in the histogram. If yes, it means *x*’ is a duplicated fragment;

otherwise not.

2.7. Commit here means delete each index in *x*' from the list *S*, meaning they have been taken.

2.8. Here *n* means for how many times we cannot find an available position for new fragments continuously. If it is larger than a predefined number *N*, we stop this searching process, meaning that we assume no position available. By default, *N* is set to twice the number of bonds of the molecule.

2.9. We now have a list of fragments that are used to make the molecule. Now we check which fragments appear more than once in this list, the "duplications", and then remove them, storing them separately (2.10 below). The leftover fragments constitute the part we call the "residue".

2.10. Refer to 2.9 above.

2.11. The residue is a list of non-duplicated fragments. Now we consider them as one whole molecule (namely a *MOL_BOND* object) although it consists of disconnected parts. We need to obtain a fragment histogram for it, similar to *f(x)* described above to the original molecule. Naively we can repeat Monte Carlo Step 1 on the residue to get such a histogram, but we have a shortcut here: We check every InChI string in the original *f(x)*, to see whether it is a fragment of the residue. If it is, check how many duplications it has, and then we obtain one histogram item. Ultimately, we obtain the histogram for the residue, still denoted as *f(x)* which will be used again.

2.12. At this point, we have repeated the above process until no further duplication can be found. In each loop we obtained a bag (or multiset) of duplications, and now we add them together to get the bag that contains all the duplications, which is exactly the multiset representation of one assembly pathway.

2.13. Lastly, based on the multiset representation, we can calculate the index of this pathway.

2.14. Run 2.1 - 2.13 for *Nstep2* (a predefined parameter) number of times, to find the pathways

that have the lowest pathway index.

3.5 Executable program manual (*AssemblyMC.exe*)

Download zip file *AssemblyMC.zip*. Unzip it and you will see six files:

1. *AssemblyMC.exe*, the executable file to calculate the shortest assembly pathways for a single molecule, which runs on Windows 10.
2. *libinchi.dll*, a dynamic-link library file that is necessary for the exe file to run. It must be in the same folder as *AssemblyMC.exe*.
3. *readme.pdf*, the manual of the program.
4. *Adenine.mol*, the mol file (a standard file format to hold the information of a molecule) of the molecule adenine. It is the input of the exe file if you wish to calculate the shortest assembly pathways for adenine. Mol files of molecules can be freely downloaded from online databases such as ChEMBL, PubChem, etc.
5. *example_Adenine_pathway.txt*, one file of the results, which will be explained later.
6. *example_Aenine_histogram.txt*, one file of the results, which will be explained later.

Keep them in the same folder. Then:

1. Download the mol file of the molecule that you wish to calculate, and put it into the same folder of *AssemblyMC.exe* (here we will just take *Adenine.mol* as an example).
2. Open Windows command prompt (*cmd.exe*), and navigate to this folder.
3. Enter `>AssemblyMC.exe Adenine.mol` and it will then analyze the file *Adenine.mol*, namely, molecule adenine.

   There are two optional parameters. The first parameter (*Nstep2*, referring to SI section 3.4) specifies how many possible assembly pathways to try in order to find the shortest one (-1 is the default value, meaning there is no limit); while the second parameter (*Nstep1*, referring to SI section 3.4) means how many fragmenting schemes to try to obtain the

fragments histogram (-1 is the default value, meaning either 1% of all possible fragmenting schemes, or 100000, whichever is greater).

The example above uses no parameter. The following example uses both parameters

```
>AssemblyMC.exe Adenine.mol 50000 100000
```

which we will take as an example to show what to expect when it runs and after it finishes. It first calculates the fragments histogram, and shows the process as

```
Please wait...
 .100000 steps to try in step 1...
 >23132 >45035 >67144
```

When the number (e.g., >67144) shown equal to the second parameter, this process to obtain the fragments distribution (Monte Carlo Step 1, referring to Fig. S3) finishes. And the message below will be displayed in the prompt:

```
 >23132 >45035 >67144 >90217
============================
====== Step 1 is Done. ======
============================
```

After that, it will start Monte Carlo Step 2 (Fig. S3) immediately. It will keep displaying the symbol “>” until 10000 pathways have been tried. Then, in the same folder, a file *Adenine_pathway.txt* will be created that is the ultimate result and contains all the information we need, which we will explain how to interpret soon (it will also display some texts in the prompt, which basically repeats what is written in the file *Adenine_pathway.txt*). Note that the program will continue to run until it has tried *Nstep2* (the first parameter you input) number of possible assembly pathways; but each time it has tried 10000 pathways, it will update and overwrite the file *Adenine_pathway.txt*. If the first parameter you input is the default value -1, you may close the program manually when you think it has tried enough possible pathways.

Now we will explain how to interpret the results contained in *Adenine_pathway.txt*. We will take the file *example_Adenine_pathway.txt* as an example to explain due to the fact that this method is Monte Carlo (thus contains randomness) so there might be slight differences at each time it runs. Indeed, *example_Adenine_pathway.txt* is the file *Adenine_pathway.txt* generated for a particular run, and we just renamed it as *example_Adenine_pathway.txt.*

```
Monte Carlo Calculating Pathway Assembly (in bonds). v1.0

The molecule to be analysed is:
InChI=1/C5H5N5/c6-4-3-5(9-1-7-3)10-2-8-4/h1-2H,(H3,6,7,8,9,10)/f/h9H,6H2
    ||  C   #8  -->  8, 10
    ||  C   #0  -->  0, 1, 2
    ||  N   #3  -->  2, 7
    ||  C   #7  -->  7, 9
    ||  C   #1  -->  0, 3, 4
    ||  N   #9  -->  4, 9
    ||  N   #4  -->  3, 8
    ||  N   #5  -->  5, 10
    ||  C   #2  -->  1, 5, 6
    ||  N   #6  -->  6
    ||      0  *1 :  #0 -- #1
    ||      8  *1 :  #4 -- #8
    ||      2  *1 :  #0 -- #3
    ||      10  *2 :  #5 -- #8
    ||      9  *1 :  #9 -- #7
    ||      1  *2 :  #0 -- #2
    ||      7  *2 :  #3 -- #7
    ||      3  *2 :  #1 -- #4
    ||      4  *1 :  #1 -- #9
    ||      5  *1 :  #2 -- #5
    ||      6  *1 :  #2 -- #6

. Total number of atoms: 15
. When hydrogen atoms (H) are excluded:
Number of atoms: 10
Number of bonds: 11

. Parameter Nstep1 = 100000
. Parameter Nstep2 = 50000

Please wait until it generates results...
==================================================
============ Printing step 2 results ============
==================================================
. 50000 pathways tried.
. Elapsed time: 25.1389 s

Number of pathways: 2
Assembly index is: 7


=================
=== Pathway 1 ===
=================
InChI=1/C2H6N2/c1-4-2-3/h2H,1H3,(H2,3,4)/f/h3H2
InChI=1/C2H5N/c1-3-2/h1H2,2H3

How many times each duplicates:
1 :  (2,7,9)
1 :  (3,8)


=================
=== Pathway 2 ===
=================
InChI=1/C2H6N2/c1-4-2-3/h2H,1H3,(H2,3,4)/f/h3H2
InChI=1/CH4N2/c2-1-3/h1H,(H3,2,3)/f/h2H,3H2

How many times each duplicates:
1 :  (2,7,9)
1 :  (3,4)
```

The block started with “| |” records how the molecule is represented in the program. The lines starting with capital letters represent atoms. For example, the first line “C #8 --> 8, 10” means the carbon (C) atom’s ID is 8 (denoted by symbol #), and it is attached by bond 8 and 10; the seventh line “N #4 --> 3, 8” means the nitrogen (N) atom’s ID is 4, and it is attached by bond 3 and 8. The lines starting with integers represent bonds. For example, the first line “0 *1 : #0 -- #1” means this bond’s ID is 0, which is a single bond (denoted by symbol *), and it connects atom 0 and atom 1; the fourth line “10 *2 : #5 -- #8” means this bond’s ID is 10, which is a double bond, and it connects atom 5 and atom 8. All other information is self-evident.

Now let’s look at the lines “Number of pathways: 2” and “Assembly index is: 7”. It tells you that the assembly number (MA) of this molecule adenine is 7 (namely, the assembly index of the shortest assembly pathways of adenine), and there are two assembly pathways with this MA, each of which is displayed in the following lines, starting with “=== Pathway i ===”. Take the first pathway as an example, each line started with “InChI=” is an InChI (International Chemical Identifier) of a chemical structure. The first line after “How many times each duplicates:”, i.e., “1 : (2, 7, 9)” means that in the multiset representation of this pathway (referring to Fig. 2d in the main text) the multiplicity is 1 for the first structure listed above “InChI=1/C2H6N2/c1-4-2-3/h2H,1H3,(H2,3,4)/f/h3H2” which is made of bond 2, 7 and 9. The second line “1 : (3, 8)” means that in the multiset representation the multiplicity is 1 for the second structure “InChI=1/C2H5N/c1-3-2/h1H2,2H3” which is made of bond 3 and 8. Therefore, the multiset representation of assembly pathway 1 is { InChI=1/C2H6N2/c1-4-2-3/h2H,1H3,(H2,3,4)/f/h3H2, InChI=1/C2H5N/c1-3-2/h1H2,2H3 }. InChI can be easily transformed to any other format by standard software (e.g., OpenBabel). Here we use OpenBabel to transform the two InChI’s into the graph representation of molecules, so this pathway can also be denoted as:

{ 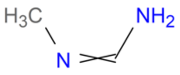

, 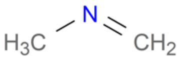

}

Note that we should ignore all of the hydrogens as we have ignored them from the beginning (these hydrogens appear in the graphs because we have to use them as some "placeholders" to generate proper InChI's).

Likewise, the multiset representation of assembly pathway 2 is { InChI=1/C2H6N2/c1-4-2-3/h2H,1H3,(H2,3,4)/f/h3H2, InChI=1/CH4N2/c2-1-3/h1H,(H3,2,3)/f/h2H,3H2 }, or denoted as:

{ 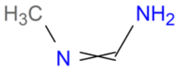

, 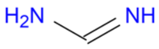

}

Lastly, there is another temporary file *Adenine_histogram.txt* also generated, that is automatically used by the program in Monte Carlo Step 2, so do not delete it. It displays the fragments histogram obtained in Monte Carlo Step 1. It might be useful to the reader, so we will also explain it (we will take *example_Adenine_histogram.txt* as an example). The first part of this file is the same as *example_Adenine_pathway.txt*, while the second half is written as follows. It displays fragments in groups (every five fragments constitute a group) which are sorted by the number of occurrences (namely, y-axis value of the fragments distribution, referring to SI section 3.3 to learn more about this distribution). So, here we see that structure InChI=1/C2H5N/c1-2-3/h2H,1,3H2 appears three times, InChI=1/CH4N2/c2-1-3/h1H,(H3,2,3)/f/h2H,3H2 appears three times, and so on; structure InChI=1/C3H5N/c1-3-4-2/h3H,1-2H2 appears twice, InChI=1/C2H6N2/c1-4-2-3/h2H,1H3,(H2,3,4)/f/h3-4H appears twice, and so on.

```
==================================================
========= Printing fragments histogram ===========
==================================================
Parameter Nstep1 = 100000
Elapsed time: 20.3378 s

InChI=1/C2H5N/c1-2-3/h2H,1,3H2
InChI=1/CH4N2/c2-1-3/h1H,(H3,2,3)/f/h2H,3H2
InChI=1/C3H6N2/c1-5-3-2-4/h2-3H,1,4H2
InChI=1/C2H5N/c1-3-2/h1H2,2H3
InChI=1/C2H6N2/c1-4-2-3/h2H,1H3,(H2,3,4)/f/h3H2
3
3
3
3
3

InChI=1/C3H5N/c1-3-4-2/h3H,1-2H2
InChI=1/C2H6N2/c1-4-2-3/h2H,1H3,(H2,3,4)/f/h3-4H
InChI=1/C3H7N/c1-2-3-4/h2-3H,4H2,1H3
InChI=1/C2H7N/c1-2-3/h2-3H2,1H3
InChI=1/C3H8N2/c1-4-3-5-2/h3H,1-2H3,(H,4,5)/f/h4H
2
2
2
2
2

InChI=1/C2H6N2/c3-1-2-4/h1-2H,3-4H2
InChI=1/C3H6N2/c1-2-5-3-4/h2-3H,1H2,(H2,4,5)/f/h4H2
2
2
```

3.6 Examples: several molecules' shortest assembly pathways calculated

Here we apply *AssemblyMC.exe* to five molecules as examples. We always set the parameters *Nstep1*=100000 and *Nstep2*=10000. The first example is aspirin. The command we run is

```
>AssemblyMC.exe Aspirin.mol 10000 100000
```

From the output of this program (see SI section 3.5 for details), we know that aspirin's MA is 8 and one of the shortest assembly pathways in multiset representation is as follows:

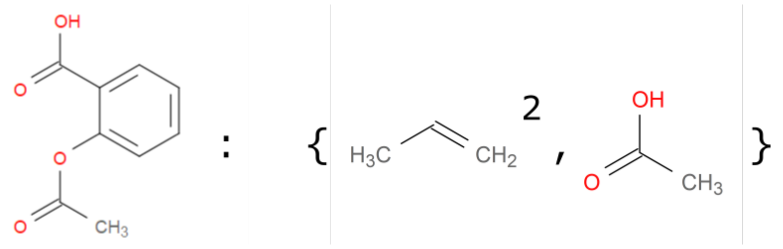

Note that we should ignore all of the hydrogens as we have ignored them from the beginning (these hydrogens appear in the graphs because we have to use them as some "placeholders" to generate proper InChI's).

Likewise, the 2nd example is hexachlorobenzene. Its MA is 5, and one of its shortest assembly pathways is:

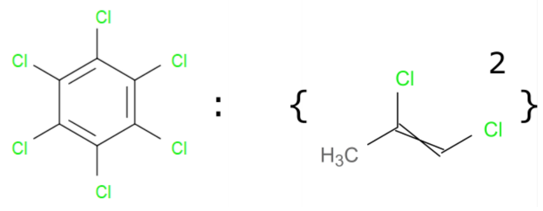

The 3rd example is tryptophan. Its MA is 11, and one of its shortest assembly pathways is:

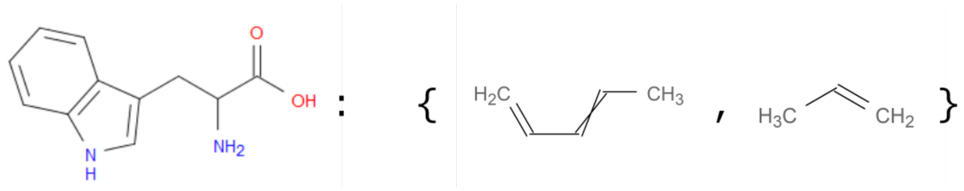

The 4th example is 5-[(1-Carboxyvinyl)oxy]-4-hydroxy-3-(phosphonooxy)-1-cyclohexene-1-carboxylic acid. Its MA is 14, and one of its shortest assembly pathways is:

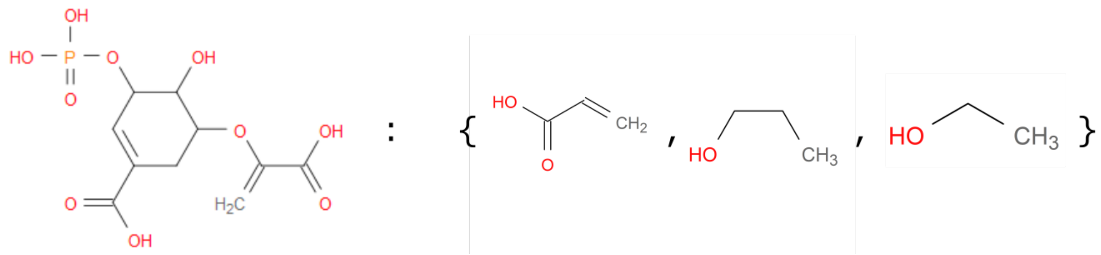

The 5th example is sildenafil, or commonly known as the brand name Viagra. Its MA is 25, and one of its shortest assembly pathways is:

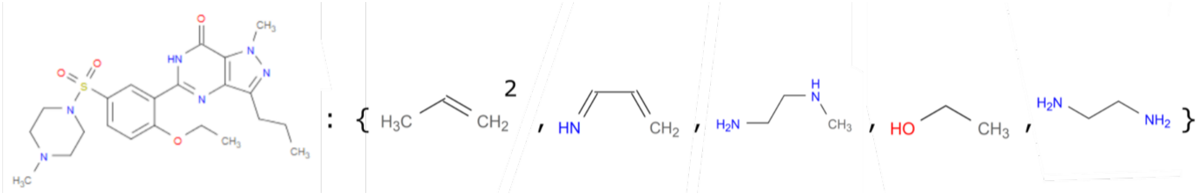

4 Monte Carlo algorithm: for molecular assembly tree

4.1 Extension to a group of molecules

So far, the Monte Carlo algorithm we have elaborated (SI section 3) computes the shortest assembly pathways of a single molecule. In fact, it can also be extended to compute the molecular assembly tree of a group of molecules (equivalently, the shortest assembly pathways to construct all of these molecules simultaneously). We only need to consider this group of molecules as a single “big molecule” with disconnected parts, which are the individual molecules. That is to say, if in the beginning of the program *AssemblyMC.exe*, we create an object that stores the complete information of this big molecule with the same format as the object that is used to store the information of a single molecule, this program can then compute the shortest pathways automatically and nothing needs to be changed after this first step.

More specifically, in *AssemblyMC.exe*, it first reads the mol file of the single molecule in question, and construct a “molecule” object (*MOL_BOND* class), which stores all of the atoms and bonds, also giving each atom an index and each bond (connecting two atoms) an index (see SI section 3.4). For a group of molecules, after creating the *MOL_BOND* object that stores the first molecule’s information, it should immediately read the second molecule’s mol file, and then add atoms and bonds of the second molecule to the previous *MOL_BOND* object. Note that the atom (or bond) index should start from the total number of atoms (or bonds) *MOL_BOND* already has, instead of starting from 0. It should continue to read mol files and add atoms and bonds, until all mol files have been read. Finally, the *MOL_BOND* object that stores the information of this big molecule can be obtained. What follows is the same process as *AssemblyMC.exe*. For convenience, we made a standalone executable file (*TreeMC.exe*, see the manual in the next subsection) to compute the assembly tree of a group of molecules.

Although the input and output have different formats to *AssemblyMC.exe*, the basic logic of the two programs is the same.

4.2 Executable program manual (*TreeMC.exe*)

Download zip file *TreeMC.zip*. Unzip it and you will the following files:

1. *TreeMC.exe* (runs on Windows), the executable file to calculate the molecular assembly tree for a group of molecules.
2. *libinchi.dll*, a dynamic-link library file that is necessary for the exe file to run. It must be in the same folder as *TreeMC.exe*.
3. *readme.pdf*, the manual of the program.
4. Mol files: *Adenine.mol*, *Guanine.mol*, *Thymine.mol*, *Cytosine.mol* and *Uracil.mol*, the mol files (a standard file format to hold the information of a molecule) of nucleobases which will be used as the example. They are the required files if you wish to compute the molecular assembly tree of these five nucleobase types. Mol files of molecules can be freely downloaded from online databases such as ChEMBL, PubChem, etc.
5. *ToDo.txt*, the required user-defined file to feed the exe file, which will be explained in details later.
6. Generated temporary files: *example_Adenine_histWhole.txt*, *example_Adenine _histWhole.txt, example_Adenine_histWhole.txt, example_Adenine_histWhole.txt* and *example_Adenine_histWhole.txt,* the temporary files generated by the program when it is running, which will be explained later.
7. Generated result files: *example_Tree_AllPaths.txt*, *example_Tree_bySize.txt* and *example_Tree_byRepeats.tx*, the files generated at the end that contains all the information we need, which will be explained later.

Now we will show how to run the program, in order to calculate the molecular assembly tree of the five nucleobase types: adenine, guanine, thymine, cytosine and uracil. As described in the main text, it is equivalent to calculate the shortest assembly pathways for these molecules.

1. Put the mol files of these five nucleobases in the folder as *TreeMC.exe*. In general, you could download these mol files from online databases, but here we have already downloaded them for you.
2. Create a plain-text file named *ToDo.txt*, based on the following format strictly (here we have created it for you):

```
Nstep2=30000
Adenine
Nstep1=100000
Thymine
Nstep1=100000
Guanine
Nstep1=100000
Cytosine
Nstep1=100000
Uracil
Nstep1=100000
$
```

   The first line is “Nstep2=XXX” where XXX is the parameter *Nstep2* for the program, meaning how many possible assembly pathways to try in order to find the shortest one (referring to SI section 3.4). The larger *Nstep2* is, the more accurate the final result will be (but it takes longer time to run). The second line is the name of one mol file: Here it is “Adenine” because the mol file is *Adenine.mol* (note that if the mol file is *abc.mol*, the second line should be “abc”). The third line is “Nstep1=XXX” where XXX is the parameter *Nstep1* for this molecule, meaning how many fragmenting schemes to try to obtain the fragments histogram for this molecule (referring to SI section 3.3). The larger *Nstep1* is, the more accurate the result will be (but it takes longer time to run). But in any case, we recommend that *Nstep1* should be at least 100000. The 4th and 5th lines are for another molecule (here it is for thymine); the 6th and 7th lines are for another (here it is guanine); and so on. Note that the order of the molecules does not matter, and *Nstep1* can be different for different molecules. Finally, the last line is the symbol “$”.

Note that any *Nstep1* could be a special value “0”, for example,

```
Adenine
Nstep1=0
```

which means that the program will use the already-existing histogram file *Adenine_histWhole.txt* instead of calculating it from scratch. If *Nstep1* is not 0, then *Adenine_histWhole.txt* is a temporary file (describing the fragments distribution, see details later) that will be generated when the program is running, and will be used again automatically until the program finishes. If *Nstep1* is set to 0, then the program will not generate / overwrite the file *Adenine_histWhole.txt*, but directly use *Adenine_histWhole.txt* to calculate the tree, which may save a lot of time (in this case, if there is no such file named *Adenine_histWhole.txt* in this folder, the program will report an error and terminate).

3. Simply double click *TreeMC*.exe to run, and wait it to finish, which may take a while.

As the program runs, progress is displayed on screen as follows for Monte Carlo Step 1 (referring to Fig. S3):

```
Please wait...

 .Adenine:
  100000 steps to try in step 1...
 >16685 >34434 >50908 >68473 >86052


Please wait...

 .Thymine:
  100000 steps to try in step 1...
 >21209 >43578 >66748 >89810


Please wait...

 .Guanine:
  100000 steps to try in step 1...
```

Every time when it obtains the fragments distribution for a molecule, a file named *XXX_histWhole.txt* will be generated or overwritten if already existed, where *XXX* is the name of the corresponding mol file. Just leave them there and they will be automatically reused in

Monte Carlo Step 2. When each molecule's fragments distribution have been obtained, it will display a message, and Monte Carlo Step 2 will start immediately:

```
============================================
====== Step 1 is done for all molecules. ======
============================================

====== Step 2 started. ======
File 1 (Adenine) InChI counts: 787
File 2 (Thymine) InChI counts: 186
File 3 (Guanine) InChI counts: 1432
File 4 (Cytosine) InChI counts: 108
File 5 (Uracil) InChI counts: 102
0:24(24), 1:21(21), 2:26(21), 3:29(21), 4:20(20), 5:23(20), 6:30(20), 7:24(20)
, 8:22(20), 9:23(20), 10:23(20), 11:26(20), 12:20(20), 13:26(20), 14:26(20), 1
5:24(20), 16:28(20), 17:22(20), 18:19(19), 19:20(19), 20:24(19), 21:31(19), 22
:22(19), 23:29(19), 24:27(19), 25:25(19), 26:23(19), 27:24(19), 28:25(19), 29:
26(19), 30:32(19), 31:26(19), 32:24(19), 33:26(19), 34:26(19), 35:28(19), 36:2
7(19), 37:22(19), 38:24(19), 39:22(19), 40:23(19), 41:24(19), 42:26(19), 43:23
(19), 44:24(19), 45:24(19), 46:26(19), 47:20(19), 48:27(19), 49:23(19), 50:24(
19), 51:26(19), 52:26(19), 53:24(19), 54:24(19), 55:21(19), 56:26(19), 57:22(1
9), 58:25(19), 59:22(19), 60:27(19), 61:32(19), 62:24(19), 63:23(19), 64:26(19
), 65:25(19), 66:24(19), 67:19(19), 68:26(19), 69:27(19), 70:25(19), 71:29(19)
```

The message "71: 29 (19)", for example, means that 71 possible pathways have been tried, the assembly index of the previous pathway is 29, and the minimum index till now is 19. This will continue until it checks *Nstep2* number of possible pathways. After that, a message will be displayed (as follows, which means that there are 5 pathways having index 16; 52 pathways having index 17; and so on) and the program finishes (result files will be generated).

```
(16), 29966:24(16), 29967:24(16), 29968:27(16), 29969:28(16), 29970:25(16), 29
971:34(16), 29972:22(16), 29973:25(16), 29974:22(16), 29975:24(16), 29976:22(1
6), 29977:19(16), 29978:28(16), 29979:26(16), 29980:24(16), 29981:19(16), 2998
2:26(16), 29983:21(16), 29984:29(16), 29985:28(16), 29986:22(16), 29987:21(16)
, 29988:29(16), 29989:25(16), 29990:27(16), 29991:22(16), 29992:24(16), 29993:
21(16), 29994:29(16), 29995:22(16), 29996:28(16), 29997:28(16), 29998:27(16),
29999:20(16),
Pathway distribution:
16:5
17:52
18:164
19:430
20:950
21:1738
22:2712
23:3666
24:4341
25:4274
26:3891
27:2928
28:2077
29:1222
30:659
31:335
32:184
33:89
34:32
35:14
36:4
Press any key to continue . . .
```

Now we will explain how to interpret the result files. The file *Tree_AllPaths.txt* is one of the three files generated at the end, which contains all the information we need. Here we will take

example_Tree_AllPaths.txt as an example to explain due to the fact that this method is Monte Carlo (thus contains randomness) so there might be slight differences at each time it runs. Indeed, *example_Tree_AllPaths.txt* is the file *Tree_AllPaths.txt* generated for a particular run, and we just renamed it as *example_Tree_AllPaths.txt*. This is how the file starts with:

```
===================
Index = 16
$
InChI=1/C4H4N2O2/c7-3-1-2-5-4(8)6-3/h1-2H,(H2,5,6,7,8)/f/h5-6H
1, 8
InChI=1/C4H9N3O/c5-2-1-3-7-4(6)8/h1,3H,2,5H2,(H3,6,7,8)/f/h7H,6H2
1, 7
InChI=1/C5H12N6/c6-1-4(10-2-7)5(9)11-3-8/h2-3H,1,6,9H2,(H2,7,10)(H2,8,11)/f/h7-8H2
1, 10
InChI=1/C2H6N2/c1-4-2-3/h2H,1H3,(H2,3,4)/f/h3H2
1, 3
InChI=1/C3H8N2/c4-2-1-3-5/h1-2H,3-5H2
1, 4

$
InChI=1/C5H8N2O2/c1-2-3-6-5(9)7-4-8/h2-4H,1H3,(H2,6,7,8,9)/f/h6-7H
1, 8
InChI=1/C6H13N5/c1-9-3-5(10-2)6(8)11-4-7/h4,9H,2-3,8H2,1H3,(H2,7,11)/f/h7H2
1, 10
InChI=1/C4H9N3O/c5-2-1-3-7-4(6)8/h1,3H,2,5H2,(H3,6,7,8)/f/h7H,6H2
1, 7
InChI=1/C2H6N2/c1-4-2-3/h2H,1H3,(H2,3,4)/f/h3H2
1, 3
InChI=1/C3H8N2/c4-2-1-3-5/h1-2H,3-5H2
1, 4

$
InChI=1/C5H8N2O2/c1-6-3-2-5(9)7-4-8/h2-4,6H,1H3,(H,7,8,9)/f/h7H
1, 8
InChI=1/C5H12N6/c6-1-4(10-2-7)5(9)11-3-8/h2-3H,1,6,9H2,(H2,7,10)(H2,8,11)/f/h7-8H2
1, 10
InChI=1/C4H9N3O/c5-2-1-3-7-4(6)8/h1,3H,2,5H2,(H3,6,7,8)/f/h7H,6H2
1, 7
InChI=1/C3H8N2/c4-2-1-3-5/h1-2H,3-5H2
1, 4
InChI=1/C2H6N2/c1-4-2-3/h2H,1H3,(H2,3,4)/f/h3H2
1, 3
```

The assembly pathways having assembly index 16 are the shortest, so they are shown first. There are 5 pathways having index 16, so they are shown one by one here (it is a long text file, and it shows only 3 pathways here. After index 16, it shows all pathways having index 17, and then 18, and so on). Each pathway stars with the symbol "$". Each pathway is made of chemical structures, represented by these InChI (International Chemical Identifier) strings. The two numbers below each InChI means the counts and the number of bonds of this structure respectively. We see that the first pathway shown above has five InChI's, corresponding to the following five chemical structures respectively (we used one of the standard software OpenBabel to transform the InChI's into graph representations of molecules):

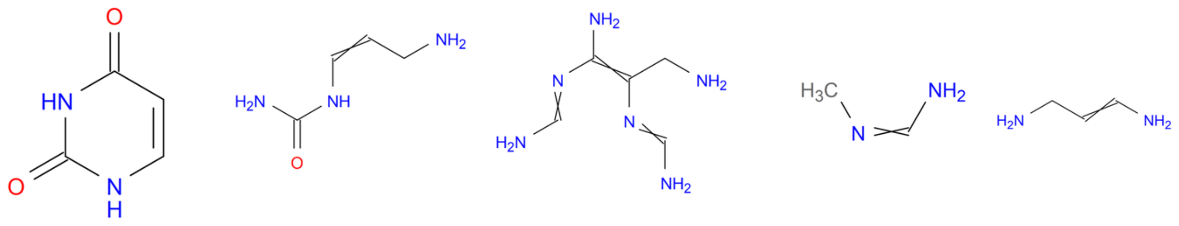

We can also confirm that the first structure has 8 bonds, the second has 7 bonds, and so on. Note that we should ignore all of the hydrogens as we have ignored them from the beginning (these hydrogens appear in the graphs because we have to use them as some "placeholders" to generate proper InChI's). Therefore, the first pathway can be written as the multiset represented:

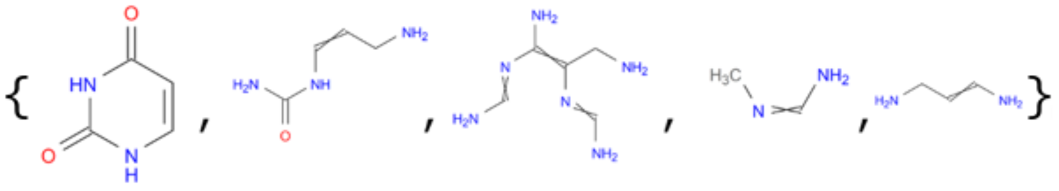

Equivalently, it is the molecular assembly tree of adenine, guanine, thymine, cytosine and uracil (written in multiset representation). We can then manually draw the molecular assembly tree of them (see details in SI section 4.3):

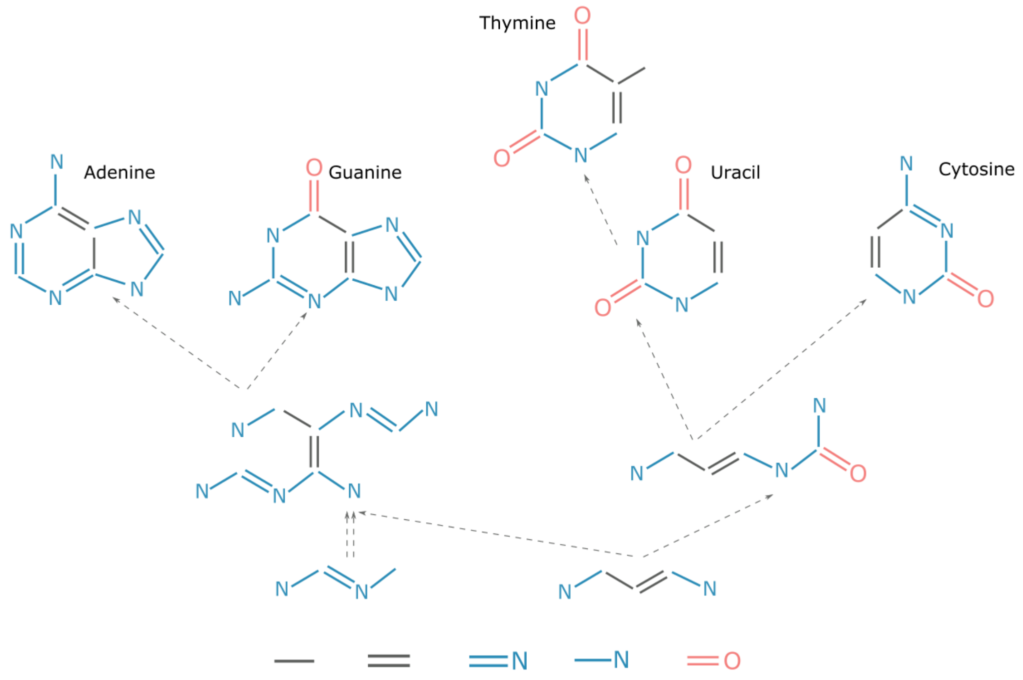

Another file generated at the end is *Tree_bySize.txt* (here we take *example_Tree_bySize.txt* as the example). The information is written as InChI, two numbers, InChI, two numbers, InChI, two numbers, … The first number below an InChI means how many assembly pathways in the file *Tree_AllPaths.txt* contain this chemical structure; and the second number is the number of bonds of this structure. The structures are sorted by the number of bonds (namely the second number). The last file generated at the end is *Tree_byRepeats.txt* (here we take *example_Tree_byRepeats.txt* as the example). It is almost the same as the file *Tree_bySize.txt*, but the structures are sorted by the first number. Some readers may be interested in these information, that's why we generated them.

Finally, we will explain the temporary files (namely, the histogram files, e.g., *Adenine_histWhole.txt*) generated when the program is running. We will take *example_Adenine_histWhole.txt* as an example. The block started with "| |" records how the molecule is represented in the program. The lines started with capital letters represent atoms. For example, the first line "C #8 --> 8, 10" means the carbon (C) atom's ID is 8 (denoted by symbol #), and it is attached by bond 8 and 10; the seventh line "N #4 --> 3, 8" means the nitrogen (N) atom's ID is 4, and it is attached by bond 3 and 8. The lines started with integers represent bonds. For example, the first line "0 *1 : #0 -- #1" means this bond's ID is 0, which is a single bond (denoted by symbol *), and it connects atom 0 and atom 1; the fourth line "10 *2 : #5 -- #8" means this bond's ID is 10, which is a double bond, and it connects atom 5 and atom 8. The information below the symbol "$" is displayed as:

```
$
InChI=1/CH4N2/c2-1-3/h1H,(H3,2,3)/f/h2H,3H2
3:
(3,4,)  (7,9,)  (8,10,)

InChI=1/C2H6N2/c1-4-2-3/h2H,1H3,(H2,3,4)/f/h3H2
3:
(2,7,9,)  (3,4,8,)  (5,8,10,)

InChI=1/C2H5N/c1-2-3/h2H,1,3H2
3:
(1,2,)  (1,5,)  (1,6,)

InChI=1/C3H6N2/c1-5-3-2-4/h2-3H,1,4H2
3:
(1,2,5,10,)  (1,2,5,7,)  (1,2,6,7,)

InChI=1/C2H5N/c1-3-2/h1H2,2H3
3:
(2,7,)  (3,8,)  (5,10,)

InChI=1/C3H7N3/c4-1-2-6-3-5/h1-3H,4H2,(H2,5,6)/f/h5H2
3:
(1,2,5,7,9,)  (1,2,5,8,10,)  (1,2,6,7,9,)
```

Each InChI is a fragment of this molecule, namely, a chemical structure. The number below means how many of this identical structure are contained in this molecule. For example, adenine contains 3 identical structures “InChI=1/CH4N2/c2-1-3/h1H,(H3,2,3)/f/h2H,3H2”. Each of the three brackets below shows which bonds constitute this structure. These temporary files are generated because we may only need to calculate the fragments distribution once. For example, if next time we want to calculate the molecular assembly tree of adenine and other molecules, we can put this already-calculated histogram file there, and set *Nstep1* for adenine to 0, the program will then directly take this file rather than calculate it from scratch.

4.3 Visualize molecular assembly tree (based on multiset representation)

Here we take the assembly tree of A, G, T, U and C as an example (Fig. 2b in the main text). First of all, we run *TreeMC.exe* on the five molecules and ultimately obtain the multiset representation of one of the shortest assembly pathways, as shown below (see SI section 4.2 for the manual).

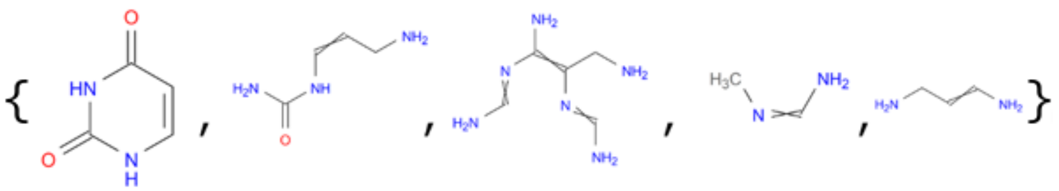

This multiset will then be used to draw the assembly trees of AGTUC. For convenience, we denote the five structures in turn as [13] (which is also U), [12], [11], [2] and [10] (to be consistent with Fig. 2b in the main text). It's more convenient to start with larger structures. We first see that T is made of [13] and a C-C bond, so we link [13] to T (referring to Fig. 2b). In principle, we should also link C-C to T, but for a better visualization, we will always omit the links from the basic building blocks, i.e., individual bonds. Second, we see that [13] is made of [12] and a C=O bond and that cytosine is made of [12] and a C=N bond, so we link [12] to both [13] and cytosine (hence a fork starting from [12]). Third, we see that A is made of [11] and a C=N bond and that G is made of [11], a C-N bond and a C=O bond, so we link [11] to both A and G. Fourth, we see that [12] is made of [10] and three individual bonds, so we link [10] to [12]. Fifth, we see that [11] is made of [10] and two of [2], so we link [10] to [11], and also link [2] to [11] (in Fig. 2b we drew two arrows from [2] to [11] to represent that [11] contains two of [2], but in general, we may just draw one, especially when the tree is big). Then we can see that [2] and [10] are just made of individual bonds (since there is no key assembly building blocks left in the multiset that have not been used), so the linking process is finished.

In fact, till now, the complete information of the tree is there, i.e., the mathematical graph consisting of nodes (structures / molecules) and edges (links). Next we employ an extra principle to arrange all the nodes: we put all nodes at different levels, and a node is always placed one level above its constituted nodes (if a node is made of several nodes, it is placed one level above its constituted node with the highest level). Finally, we obtained Fig. 2b.

There is one more remark. Although the Monte Carlo algorithm generates good-enough result, it is not guaranteed to find the shortest assembly pathway. The more Monte Carlo steps the algorithm tries, the shorter pathways it finds. But by using the suggested parameters in this paper, the results are good enough. For example, the ten opiates in Fig. 6 in the main text, which in total have 268 bonds, are certainly a large group of complex molecules; and the final output from one run of the Monte Carlo algorithm shows that the smallest MA is 105, which is already much smaller than the number of bonds (a good-enough result, see below):

```
Index = 105
$
InChI=1/C3H8/c1-3-2/h3H2,1-2H3
8, 2
InChI=1/C2H6O/c1-2-3/h3H,2H2,1H3
5, 2
InChI=1/C3H9N/c1-2-3-4/h2-4H2,1H3
4, 3
InChI=1/C3H8O/c1-3-4-2/h3H2,1-2H3
4, 3
InChI=1/C3H6/c1-3-2/h3H,1H2,2H3
4, 2
InChI=1/C4H6/c1-3-4-2/h3-4H,1-2H2
3, 3
InChI=1/C12H25N/c1-5-8-10-12(7-3)13(4)11-9-6-2/h5,8,12H,6-7,9-11H2,1-4H3
2, 12
InChI=1/C4H8/c1-3-4-2/h3-4H,1-2H3
2, 3
InChI=1/C9H14/c1-4-6-7-8-9(3)5-2/h4,6-8H,1,5H2,2-3H3
2, 8
InChI=1/C6H8/c1-3-5-6-4-2/h3-6H,1-2H2
2, 5
InChI=1/C2H7N/c1-2-3/h2-3H2,1H3
2, 2
InChI=1/C10H16/c1-4-7-9-10(6-3)8-5-2/h4,6-7,9H,3,5,8H2,1-2H3
1, 9
InChI=1/C7H10/c1-3-5-7-6-4-2/h3-7H,1H2,2H3
1, 6
InChI=1/C10H16/c1-4-7-10(8-5-2)9-6-3/h4-5,7-8H,1,6,9H2,2-3H3
1, 9
InChI=1/C5H12O/c1-3-4-5-6-2/h3-5H2,1-2H3
1, 5
InChI=1/C6H15N/c1-4-6-7(3)5-2/h4-6H2,1-3H3
1, 6
InChI=1/C4H10O/c1-2-3-4-5/h5H,2-4H2,1H3
1, 4
InChI=1/C6H16N2/c1-8-6-4-2-3-5-7/h8H,2-7H2,1H3
1, 7
InChI=1/C10H16/c1-4-6-7-9-10(3)8-5-2/h4,6-7,9H,3,5,8H2,1-2H3
1, 9
InChI=1/C9H14/c1-4-6-7-8-9(3)5-2/h4,6-8H,3,5H2,1-2H3
1, 8
InChI=1/C4H10/c1-3-4-2/h3-4H2,1-2H3
1, 3
InChI=1/C4H11N/c1-3-4-5-2/h5H,3-4H2,1-2H3
1, 4
InChI=1/C4H11N/c1-4-5(2)3/h4H2,1-3H3
1, 4
InChI=1/C3H9N/c1-3-4-2/h4H,3H2,1-2H3
1, 3
```

Nevertheless, for a large group of complex molecules, we may use human intuitions to improve the final results. We see from the InChI's above that structure [14] (figure below, which is the highlighted InChI above) is included in this assembly pathway and is shared by two molecules, morphine and thebaine. But evidently, there is a larger shared structure [15] (figure below) that completely contains [14] but the algorithm failed to find (might be able to find it in other runs). So, if we replace [14] with [15] in the multiset, we can certainly have a shorter pathway. You may find more such larger shared structures that lead to shorter pathways. Indeed, we have used this type of intuitions to draw Fig. 6. The bottom line is that, although the Monte Carlo algorithm may not be able to find the "ultimate" shortest pathways (since the problem itself is extremely hard when the size of the molecules is large, at least as hard as a NP-complete problem, see SI section 3.2), it is able to find short enough pathways, which might be further used as the bases to work out even shorter pathways.

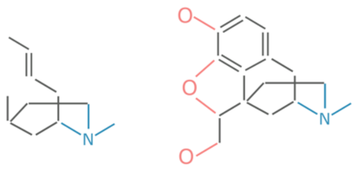

[14] [15]

5 Assembly trees for alternated nucleobases

As mentioned in the main text, the closer the biomolecules are in the assembly tree, the more effort can be saved (and thus more likely for the emergence of life). This is arguably why biomolecules exploited by extant life appear close in the assembly tree indeed else they would be too complex to emerge individually. To test this idea, we built assembly trees for alternated nucleobases and showed their MA's.

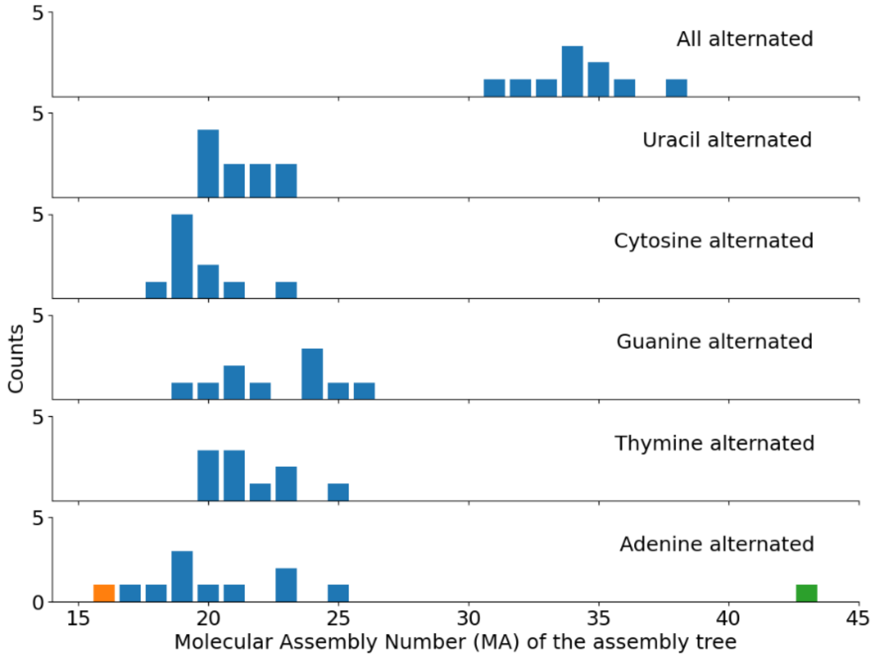

Fig. S4. Molecular assembly number (MA) of the assembly trees of alternated nucleobases. The top figure shows MA's of ten assembly trees where A, T, G, C and U are all alternated. It is a histogram, so the value on y-axis represents how many trees have such MA. In the 2nd, 3rd, 4th, 5th and 6th figure, only one nucleobase is alternated and all other nucleobases are the original. The orange bar at the left end is MA for the tree of real nucleobases; while the green bar at the right end is the assembly index of the longest assembly pathway to construct all of the real nucleobases. The green bar is located at 43 because if for each assembly step only one bond is added, 43 steps are then needed to construct all of the five real nucleobases (which is evidently the longest).

First, we generated ten random molecules that have the same formula as adenine $C_5H_5N_5$ (we call them alternated adenines). We did this by using the commercial software MOLGEN 5.0 (*57*), which enumerates all structures for a given molecular formula, or formula range. MOLGEN commands we used to enumerate molecules took the form:

$$> \text{ mgen C6H6 } - \text{v}$$

which will enumerate all structural isomers with molecular formula C_6H_6, or

$$> \text{ mgen C0} - \text{10S0} - \text{10N0} - \text{10O0} - \text{10H0} - \text{100 } - \text{sum C} + \text{N} + \text{O} + \text{S} = 10$$

which will enumerate all structural isomers with up to 10 atoms of each of C, N, O, and S, and with a total of 10 atoms of C, N, O, and S, and up to 100 H atoms (an arbitrary high figure chosen to represent any number of H atoms).

Then, we built ten assembly trees for each of these alternated adenines and the rest nucleobases G, T, C, U. We show MA's of these ten assembly trees at the bottom of Fig. S4 (as a histogram). We also show MA's of the assembly trees with alternated T, G, C, U, or all nucleobases alternated, to see how the tree compared to other possible ones in the chemical space. We can see that if one nucleobase is alternated, the assembly tree's MA is slightly larger than the real MA which is 16 as mentioned above, because the rest of the nucleobases still have quite a few common structures; while if all nucleobases are alternated, the assembly tree's MA is much larger, because few structures are shared. That is to say, even though it's straightforward to see the fact that MA of an assembly tree is always subadditive (i.e., <=) with the assembly space of the molecules (while, when the molecules share no structural motifs, for example comparing an inorganic ion to DNA, the subadditivity chooses the equal sign), the small MA 16 is not because of this property of subadditivity. Therefore, if nature randomly "selects" nucleobases (with similar size as AGTCU) as the fundamental units of genetic code, MA of their assembly tree should be around 35 (Fig. S4). However, the real MA is 16. These results demonstrate that the extant nucleobases have been selected from a relatively small subset of the chemical space even with the molecular formulas fixed, indicating that biologically relevant molecules are not arbitrary but a consequence of natural evolution for millions of years.

6 Size of assembly space of the exemplified opiates

There are two parameters for the Monte Carlo steps: *Nstep1*, meaning how many fragmenting schemes to try to obtain the fragments distribution, and *Nstep2*, meaning how many possible

assembly pathways to try in order to find the shortest one (more details in SI section 3.4). Now we'll show how the size of the calculated assembly space – the total number of unique duplicated fragments included in all assembly pathways calculated – of the exemplified opiates (referring to section "Opiates" in the main text) changes with *Nstep1* and *Nstep2*, as shown in Fig. S5.

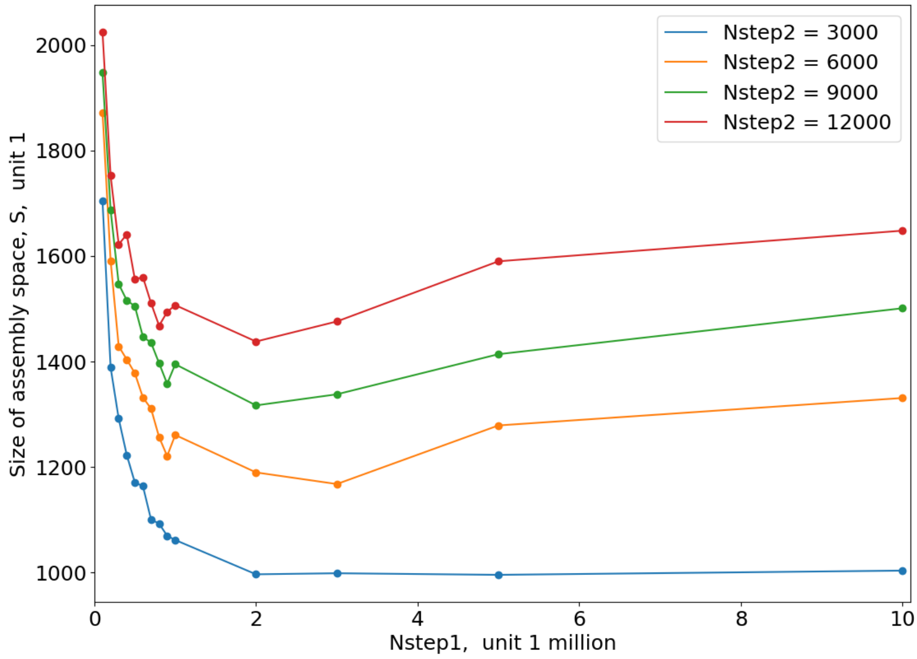

Fig. S5. How the size of the calculated assembly space, i.e., the total number of unique duplicated fragments included in all assembly pathways calculated, of the exemplified opiates in section "Opiates" in the main text changes with *Nstep1* and *Nstep2*.

There is a trend that as *Nstep1* increases, the size of the assembly space (*S*) first decreases sharply and then increase slowly. When *Nstep1* is small (for example, 0.5 million in this case), the fragment distribution would not be able to reflect the "real" distribution (as we use Monte Carlo approach to sample). The distribution is more spread out than it should be, i.e., the probability of drawing a fragment duplicated for a small number of times would be higher than it should be, while the probability of drawing a fragment duplicated for a large number of times would be lower than it should be. Therefore, in this case, the number of unique fragments that

are drawn in all these processes would be larger than that in the case where the fragment distribution can reflect the "real" distribution. In this case, the threshold is around 2 million (as see from the figure above), after which the distribution is almost "real". Now, *S* starts to increase, because the fragments that are duplicated for only very few times (twice, for example) have the chance to be drawn, which is very unlikely before this threshold. The increase of *S* is slow, because as long as this threshold is passed, the calculated fragment distribution is already quite close to the real one.

We also observed that the larger *Nstep2* is, the larger *S* is. This is straightforward, because the more steps we try, the more unique fragments would be drawn. If *Nstep2* goes to infinite, all unique fragments that are duplicated at least once would be drawn, i.e., the size of the assembly space then would be the number of all these unique fragments. But in this case, even when *Nstep2*=3000, the calculated assembly tree's MA (which is 105) is already very small, comparing to the number of bonds (which is 268). Therefore, all the unique fragments that have been drawn (namely, the assembly space obtained) already reflect the contingent information of these compounds in question, which is what we care about.

7 Assembly-pool-based *de novo* molecule generation

7.1 The *Reassembler*

The *Reassembler* is the name of a method used to reconnect the assembly pool fragments, written in Python and based on RDKit library. More specifically, it takes two mol objects describing the fragments, enumerates all possible combinations thereof and returns them as new mol objects. It does so by the means of dummy reaction SMARTS meant to emulate, in reverse, the disconnections made by the Molecular Assembly. The pattern by which connections happen is by fusing the atoms together (see Fig. S6). This way all the bonds are

preserved, as they are considered the actual building blocks in the MA. The additional advantage of this is greater access to skeletal complexity.

Technically, the effect of "atom fusion" is obtained by such reaction SMARTS, where one of the atoms (the "donor") has all its substituents transferred to the other atom (the "acceptor")

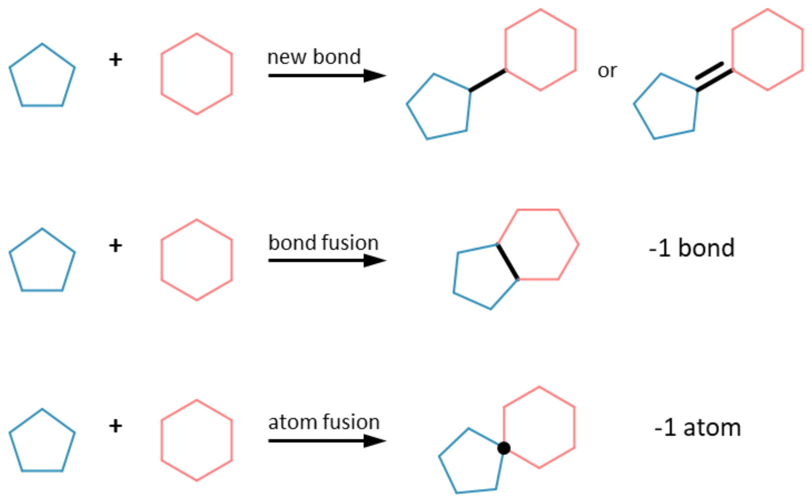

Fig. S6. When connecting fragments, perhaps the most obvious way is to insert new bonds between the atoms (top), however, this requires some arbitrary choices (e.g. bond type) and limits the complexity of products. Fusing the fragments by either bonds (middle) or atoms (bottom) allows to make connections without introducing extra elements. The *Reassembler* and Molecular Assembly follow the last pattern, atom fusion, which preserves all the bonds but reduces the number of atoms. The bond fusion is not allowed as it would result in the loss of bonds.

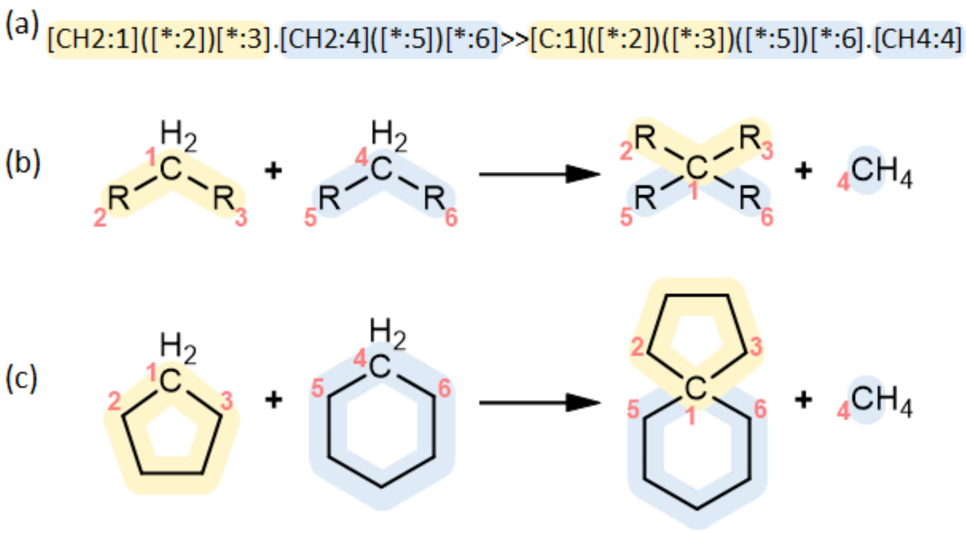

Fig S7. An example of dummy reactions used by the *Reassembler* to reverse-emulate the Molecular Assembly disconnections. It is encoded by the SMARTS string shown in (a) that is interpreted using RDKit for the purpose of predicting possible connections between the two assembly fragments. Even though the *Reassembler* effectively "fuses" the atoms and preserves all the bonds, one of the atoms is actually excluded as shown in (b), while the substituents are transferred to the other atom (i.e., from atom 4 to atom 1, with the exclusion of atom 4). Such a connection pattern allows for great skeletal complexity, whereby if fragments are cyclic as shown in (c), the assembly product is a spiro compound.

and is subsequently excluded from the molecule. Such connections are possible when both atoms belong to the same element and the sum of their non-hydrogen substituents does not exceed the maximum number any of them may have, e.g., two secondary carbons can be connected (see Fig. S7) but a tertiary and a secondary carbon cannot, as it would result in a pentavalent carbon. Every SMARTS covers an individual case and there is total of 540 reactions: 14 single-connection, 512 two-connection and 14 single-internal-connection reactions (the latter called *origami*), which are exemplified in the Fig. S8.

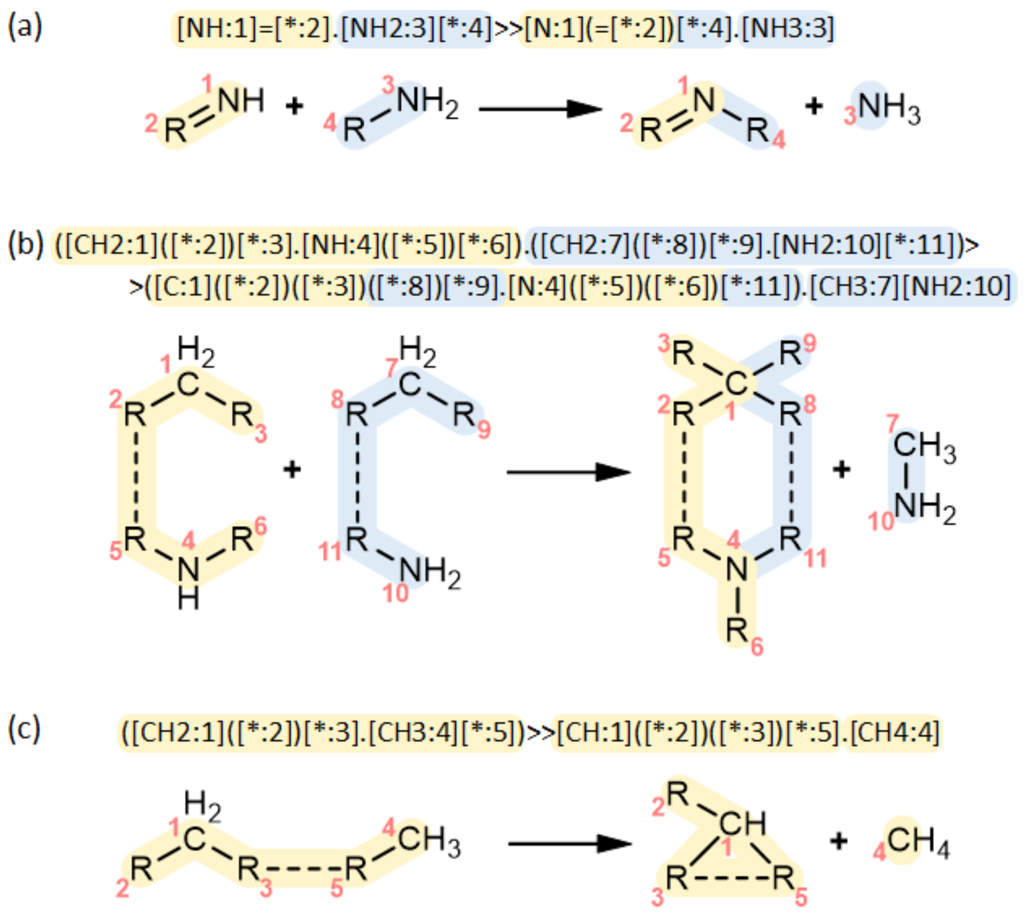

Fig. S8. The *Reassembler* uses three sets of reaction SMARTS. The n=1 set is comprised of intermolecular "reactions" that fuse together one atom from each "substrate", as exemplified in (a). Notice that different hybridization atoms are allowed to be fused as long as the result is chemically valid. The n=2 set consists of intermolecular "reactions" that connect molecules by two independent pairs of atoms, as exemplified in (b). To obtain connections with n>2, the origami set is used to emulate intramolecular reactions, whereas the product of a n=2 reaction undergoes a series of internal "cyclizations", as shown in (c). Pink numbers are used to keep track of atoms, while yellow and blue highlights denote the starting fragment from which the specific bonds atom atoms originate. Only yellow highlight is used in the example (c) to emphasize that origami transformations are always intramolecular.

7.2 Python script to use the *Reassembler* to generate new molecules (*GenMols.py*)

Download zip file *GenMols.zip*. Unzip it and you will see five files:

1. *GenMols.py*, the python module to generate new molecules from an assembly pool, which is the main entry.

2. *Reassembler.py*, the python module that will be automatically imported into GenMols.py. It is explained in details in SI section 7.1.
3. *Pool.txt*, the assembly pool we used, which will be explained later.
4. *RandomPool.txt*, another assembly pool, which will be explained later.
5. A Folder *DataForPaper,* containing the InChI strings and pictures of the "assembled molecules", "random molecules" and "unconstrained molecules" that are mentioned in the paper.

Pool.txt contains InChI strings, separated by a single line which could be any text or blank. All of these InChI strings constitute the assembly pool you will use to generate new molecules. In our example, it is the assembly pool of the six natural opiates (morphine, codeine, noscapine, oripavine, papaverine, and thebaine), which is calculated by our program *TreeMC.exe* (see SI section 4.2). In fact, *Pool.txt* is exactly the file *Tree_bySize.txt* generated by *TreeMC.exe* in the end (just renamed), because *Tree_bySize.txt* is the calculated assembly pool. The new molecules generated from *Pool.txt* are the "Assembled" molecules.

RandomPool.txt is another assembly pool we used, which has the same format as *Pool.txt*. We constructed it manually: (1) the six natural opiates (morphine, codeine, noscapine, oripavine, papaverine, and thebaine) have 76 C-C bonds (whose InChI is "InChI=1S/C2H6/c1-2/h1-2H3") in total, so there are 76 lines of "InChI=1S/C2H6/c1-2/h1-2H3"; they have 31 C=C bonds, so there are 31 lines of "InChI=1S/C2H4/c1-2/h1-2H2"; and so on. By using this file as the assembly pool, the generated molecules will be "random" because it contains almost no information of these six opiates, except the bond composition (note that we could use an assembly pool consisting of arbitrary single bonds to generate random molecules, but in that case, they will be too random, that is, too different comparing to the original six opiates. Then it makes no sense to compare them with the "assembled" molecules. So, here, our "random" molecules still resemble the composition of the original six opiates but contain no structural

/contingent information the original six opiates hold). The new molecules generated from *RandomPool.txt* are the “Random” molecules.

Now we explain how to run the script. In Python, simply run

```
> Import GenMols
> GenMols.run(10)
```

and it will give you 10 new molecules from the assembly pool file *Pool.txt*. It may take a while but eventually will generate one text file *newMols.txt* containing all the InChI strings of the generated molecules, and another folder *MolsFig* containing all the pictures of the generated molecules.

In GenMols.run(), there are a few parameters you can change:

```
> GenMols.run(NmolNeeded, PoolFile = 'Pool.txt', OutputInchiFile = 'newMols.txt', OutputFigPath = 'MolsFig', mwMin = 281, mwMax = 368, DoUMin = 9, DoUMax = 12, oneAtomWeight = 12, mwDelta = 0.1)
```

Here, NmolNeeded (int): the number of molecules needed to be generated. PoolFile (str): path to the assembly pool txt file. OutputInchiFile (str): file name where the output InChI strings will be written into. OutputFigPath (str): path of a folder where the pictures of the newly-generated molecules will be put into. mwMin (int) and mwMax (int): minimum and maximum molecular weight of newly-generated molecules. Here the default values are 281 and 368, which are the minimum and maximum molecular weight of the six natural opiates we used. DoUMin (int) and DoUMax (int): minimum and maximum Degree of Unsaturation (DoU) of newly-generated molecules. Here the default values are 9 and 12, which are the minimum and maximum DoU of the six natural opiates we used. oneAtomWeight (int): an approximate molecular weight lost when an atom is thrown away when two fragments are combined. Here the default value is set to be 12, the molecular weight of a carbon atom, since overlapping

carbon atom is the most frequent event (do not need to change in most cases). mwDelta (float): give a range of the molecular weight that could be relaxed. Here the default value is set to be 0.1 (10%) (do not need to change in most cases).

The function *GenMols.run()* generates new molecules in the following steps:

(1) Randomly choose an arbitrary number of fragments (namely, InChI strings) from the assembly pool, such that the total molecular weight is approximately between mwMin and mwMax. Let's call this set of fragments set S.

(2) Randomly pick two fragments M1 and M2 from S (remove M1 and M2 from S), and apply *Reassembler*(M1, M2, *n*) to combine them, where *n* is the number of atoms M1 and M2 will be overlapped, that is, if $n = 1$, M1 and M2 will be joined in one atom site, while if $n = 2$, M1 and M2 will be joined in two different atom sites. Here *n* can only be 1 or 2, and is randomly determined. Refers to SI section 7.1 for details about how *Reassembler()* works.
If the combination succeeds, put the combined fragment back into S; otherwise (i.e., when *Reassembler()* returns *None*), go back to step (1).

(3) Repeat from step (2) until there is only one fragment left in S. Denote this fragment as molecule X.

(4) Check X's Degree of Unsaturation (DoU). If X's DoU is between DoUMin and DoUMax, go to the next step (5) directly. If it is larger than DoUMax, go back to step (1). If it is smaller than DoUMin, apply the function *Origami()* for *k* times to obtain a new molecule, and denote it as X, instead. The function *Origami()* makes a molecule undergo an internal connection with itself, that is, by applying *Origami()* once, the molecule's DoU is increased by 1 (referring to SI section 7.1 for details how *Origami()* works). *k* is randomly determined such that the new molecule has DoU between DoUMin and DoUMax.

(5) Put X into a function *filterMol()* to check if it is an obviously impossible molecule. If yes, go back to step (1); otherwise, go to the next step (6). More specifically, *filterMol()* filters forbidden structures and structural motifs that are commonly used, represented with SMARTS strings and modified in such a way that every “single bond” is replaced with “any bond” and all atoms are “any atoms” (*57*).

(6) Put X into a function *confilter()* to check if it does not have any valid conformation. If yes, go back to step (1); otherwise, this molecule X is one of such new molecules that will be returned. More specifically, *confilter()* uses the EmbedMolecule() method from RDKit to find a conformation of the molecule (*58*). In the most drastic cases, the molecules connectivity is so impossible that no conformation could be found at all and the method returns -1, which results in the molecule being rejected. Keep in mind, the implemented filters do not reject antiaromatic compounds or long cumulenic structures (e.g., acyclic R-C=C=C=C-R).

(7) Repeat from step (1) to generate more molecules.

(8) After generating enough new molecules, all of their InChI strings will be written into a text file (*newMols.txt* by default), and all of their pictures are put into a folder (*MolsFig* by default). Then the whole program stops.

7.3 Pseudo-opiates

The following chapter provides details and depictions of some of the generated pseudo-opiates. For clarity purposes, the shown double bond (cis-trans) isomerism is arbitrary as the actual assembly products have unspecified stereochemistry. All generated molecules are available from the supplement files, stored in the *GenMols.zip* file (referring to the file GenMols/DataForPaper/Assembled/newMols.txt for the InChI strings of the “Assembled molecules”, the file GenMols/DataForPaper/Random/newMols.txt for the InChI strings of the

"Random molecules", and file GenMols/DataForPaper/Unconstrained/newMols.txt for the InChI strings of the "Unconstrained molecules").

compound

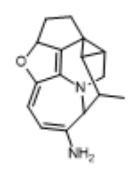

identification
AP1
number 61

properties
QED 0.7153
logP 2.4245

similarity
codeine 0.2291
morphine 0.2366
noscapine 0.1382
oripavine 0.2041
papaverine 0.0588
thebaine 0.1980

compound

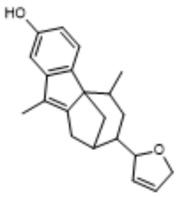

identification
AP4
number 199

properties
QED 0.7773
logP 4.438

similarity
codeine 0.2404
morphine 0.2475
noscapine 0.1527
oripavine 0.2772
papaverine 0.1230
thebaine 0.2453

compound

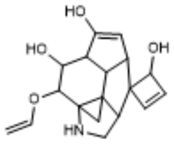

identification
number 94

properties
QED 0.4399
logP 0.4727

similarity
codeine 0.1532
morphine 0.1682
noscapine 0.0714
oripavine 0.1130
papaverine 0.0462
thebaine 0.0917

compound

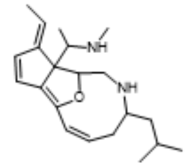

identification
number 256

properties
QED 0.8251
logP 3.7139

similarity
codeine 0.1404
morphine 0.1339
noscapine 0.1176
oripavine 0.1404
papaverine 0.1040
thebaine 0.1466

compound

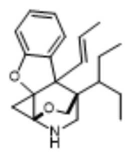

identification
number 95

properties
QED 0.8545
logP 3.7877

similarity
codeine 0.1379
morphine 0.1316
noscapine 0.1407
oripavine 0.1579
papaverine 0.0937
thebaine 0.1638

compound

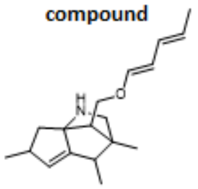

identification
number 275

properties
QED 0.4792
logP 3.6732

similarity
codeine 0.1019
morphine 0.0841
noscapine 0.0846
oripavine 0.1121
papaverine 0.0672
thebaine 0.1193

compound

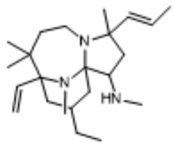

identification
number 161

properties
QED 0.7498
logP 4.4177

similarity
codeine 0.1083
morphine 0.0924
noscapine 0.0915
oripavine 0.1083
papaverine 0.0522
thebaine 0.1240

compound

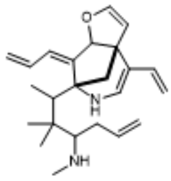

identification
number 347

properties
QED 0.6141
logP 4.6397

similarity
codeine 0.0960
morphine 0.0894
noscapine 0.0816
oripavine 0.1048
papaverine 0.0902
thebaine 0.1111

compound

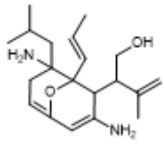

identification
number 175

properties
QED 0.6531
logP 3.0061

similarity
codeine 0.1043
morphine 0.1071
noscapine 0.0797
oripavine 0.1043
papaverine 0.0714
thebaine 0.1017

compound

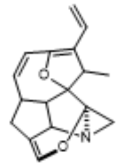

identification
number 481

properties
QED 0.6806
logP 2.3457

similarity
codeine 0.1863
morphine 0.1919
noscapine 0.1172
oripavine 0.1524
papaverine 0.0661
thebaine 0.1481

compound

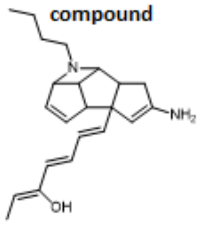

identification
number 188

properties
QED 0.4358
logP 4.0782

similarity
codeine 0.1858
morphine 0.1909
noscapine 0.0833
oripavine 0.1261
papaverine 0.0597
thebaine 0.1230

compound

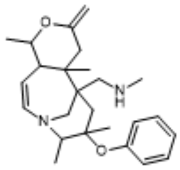

identification
number 533

properties
QED 0.8067
logP 4.5964

similarity
codeine 0.2203
morphine 0.2051
noscapine 0.1608
oripavine 0.1901
papaverine 0.1429
thebaine 0.1951

Fig. S9a. Examples of opiate-like assembly products. Identification numbers correspond to the supplementary files (GenMols/DataForPaper/Assembled/newMols.txt). Names AP1 and AP4 correspond to Fig. 7 in the main text.

compound

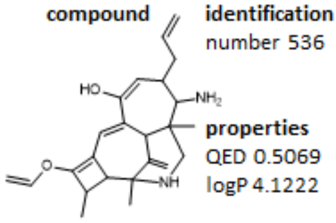

identification
number 536

properties
QED 0.5069
logP 4.1222

similarity
codeine 0.1488
morphine 0.1429
noscapine 0.1027
oripavine 0.1488
papaverine 0.0970
thebaine 0.1360

compound

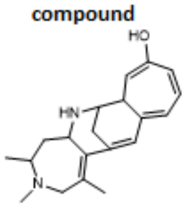

identification
AP6
number 750

properties
QED 0.7208
logP 3.2517

similarity
codeine 0.2190
morphine 0.2255
noscapine 0.1450
oripavine 0.2427
papaverine 0.1057
thebaine 0.2243

compound

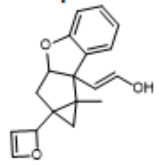

identification
number 569

properties
QED 0.8441
logP 3.4697

similarity
codeine 0.1827
morphine 0.1881
noscapine 0.1508
oripavine 0.1714
papaverine 0.0917
thebaine 0.1560

compound

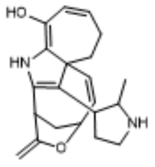

identification
number 783

properties
QED 0.6413
logP 3.4363

similarity
codeine 0.1947
morphine 0.2110
noscapine 0.1135
oripavine 0.1947
papaverine 0.0916
thebaine 0.1695

compound

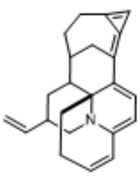

identification
number 576

properties
QED 0.7548
logP 4.2919

similarity
codeine 0.1880
morphine 0.1930
noscapine 0.1180
oripavine 0.1983
papaverine 0.0889
thebaine 0.1833

compound

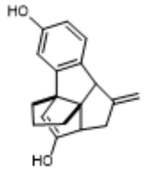

identification
AP3
number 887

properties
QED 0.6984
logP 3.9291

similarity
codeine 0.2121
morphine 0.2447
noscapine 0.1639
oripavine 0.2500
papaverine 0.1130
thebaine 0.2178

compound

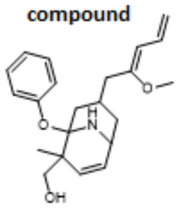

identification
number 629

properties
QED 0.4437
logP 3.8047

similarity
codeine 0.1709
morphine 0.1453
noscapine 0.1277
oripavine 0.1610
papaverine 0.1417
thebaine 0.1667

compound

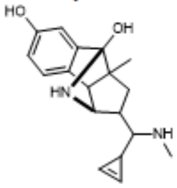

identification
AP2
number 913

properties
QED 0.6372
logP 1.4065

similarity
codeine 0.2157
morphine 0.2347
noscapine 0.1406
oripavine 0.2039
papaverine 0.1282
thebaine 0.1406

compound

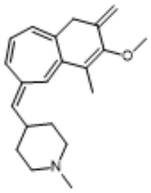

identification
AP5
number 664

properties
QED 0.7413
logP 4.5575

similarity
codeine 0.0207
morphine 0.1682
noscapine 0.1905
oripavine 0.2308
papaverine 0.1333
thebaine 0.2359

compound

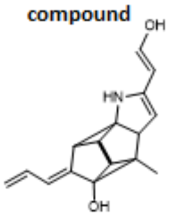

identification
number 985

properties
QED 0.6540
logP 1.467

similarity
codeine 0.0841
morphine 0.0971
noscapine 0.0454
oripavine 0.0642
papaverine 0.0333
thebaine 0.0531

Fig. S9b. Examples of opiate-like assembly products. Identification numbers correspond to the supplementary files (GenMols/DataForPaper/Assembled/newMols.txt). Names AP2, AP3, AP5 and AP6 correspond to Fig. 7 in main text.

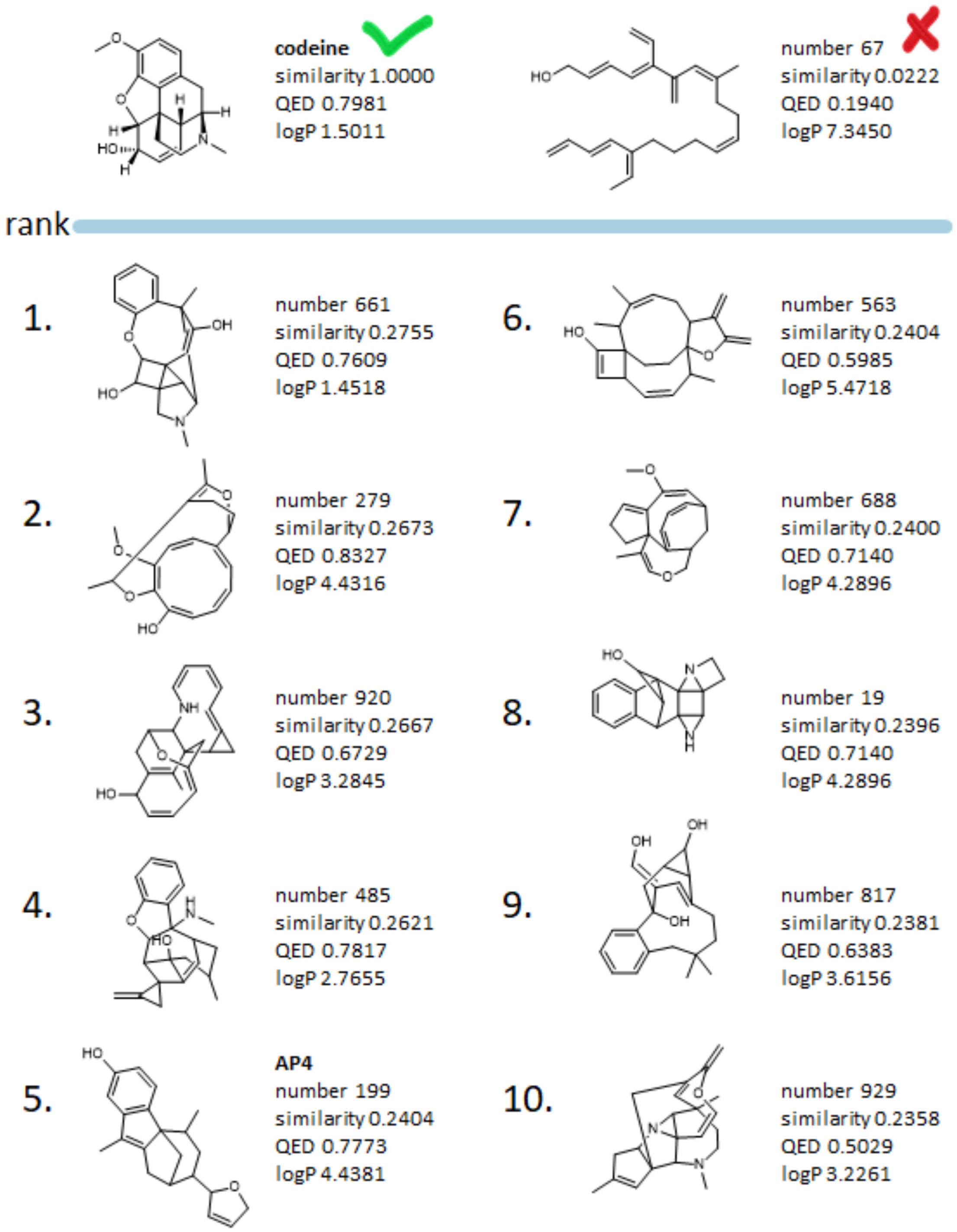

Fig. S10. Ten most codeine-like assembly products ranked according to their Tanimoto scores. For comparison purposes, codeine and the least-similar product are shown on top.

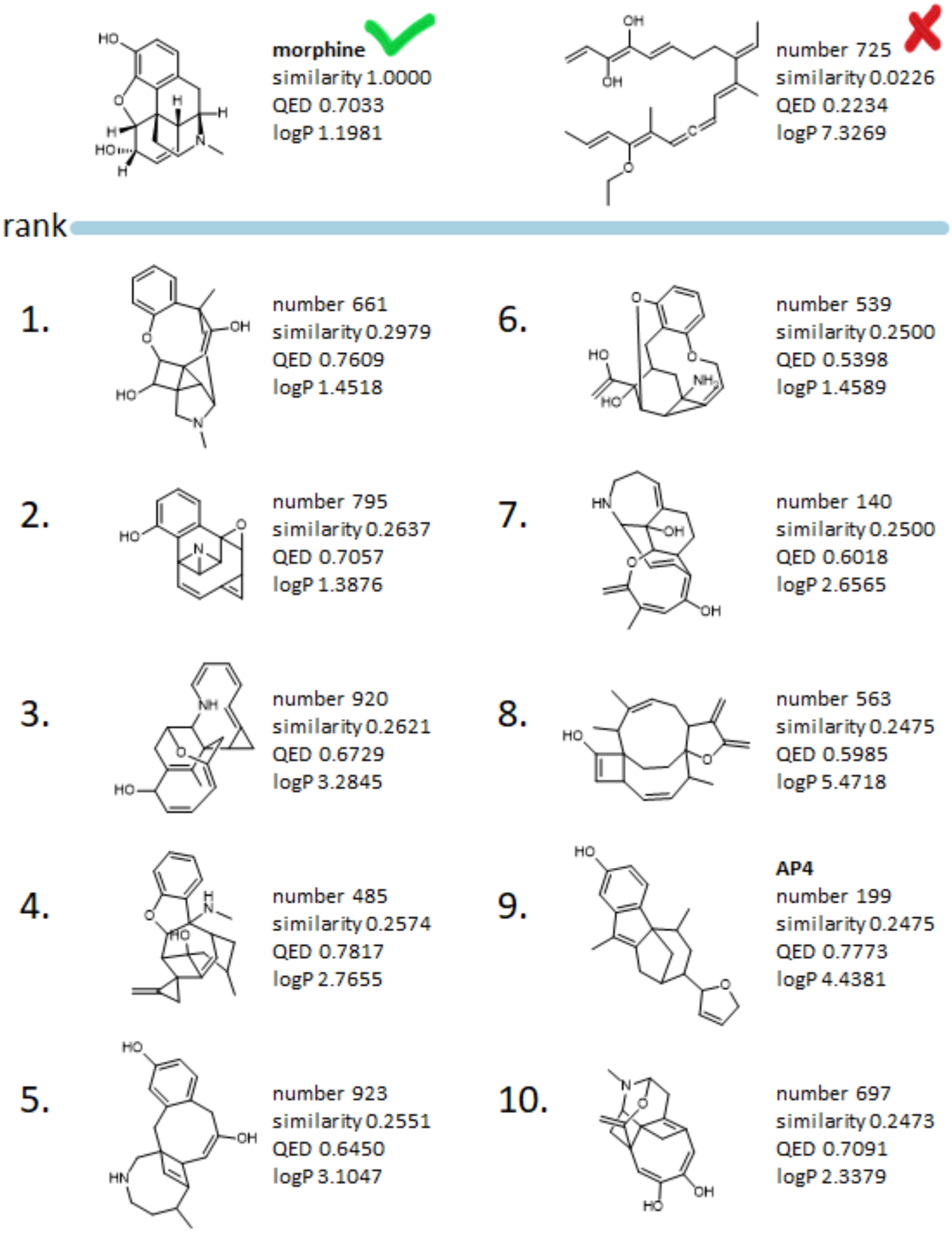

Fig. S11. Ten most morphine-like assembly products ranked according to their Tanimoto scores. For comparison purposes, morphine and the least-similar product are shown on top.

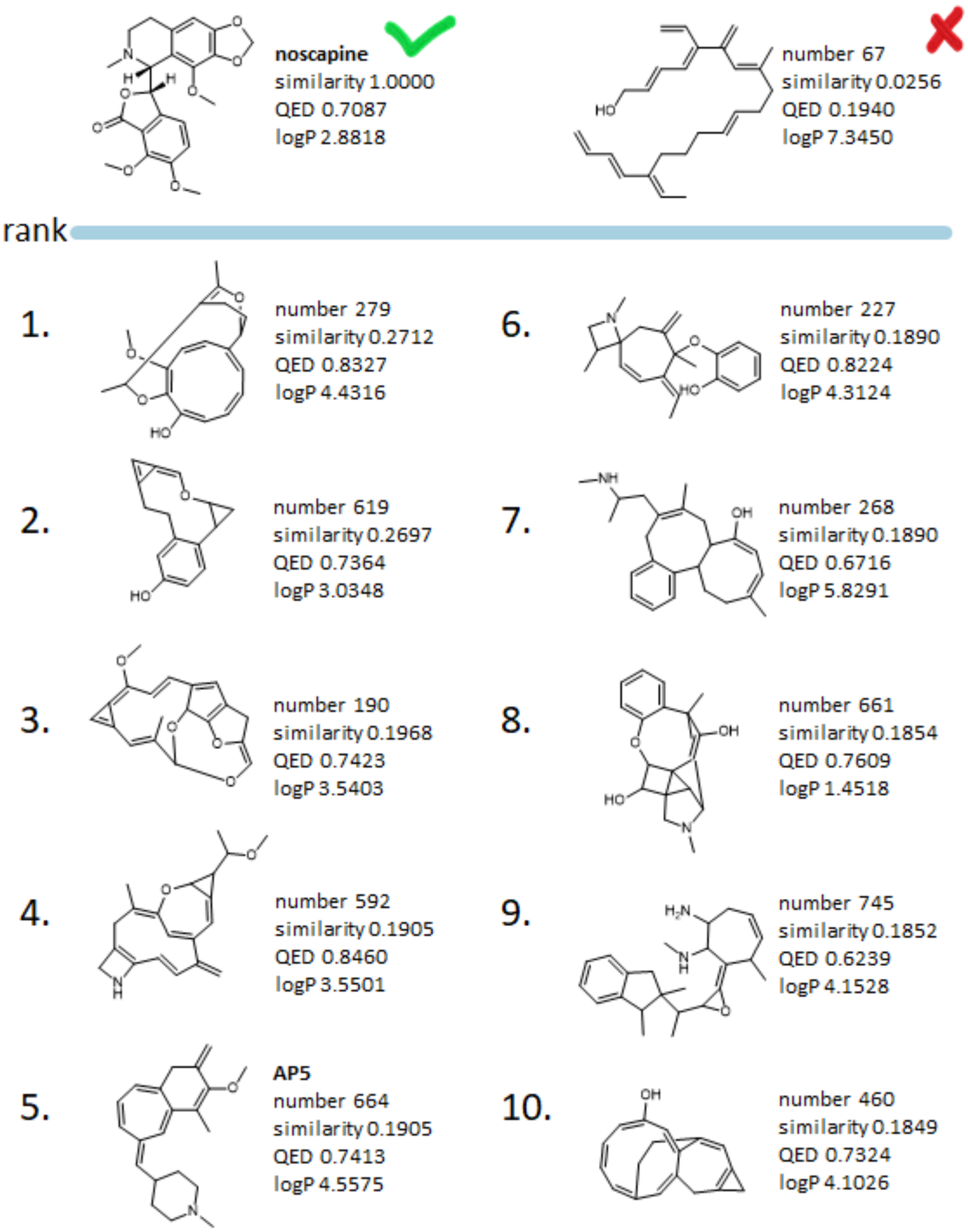

Fig. S12. Ten most noscapine-like assembly products ranked according to their Tanimoto scores. For comparison purposes, noscapine and the least-similar product are shown on top.

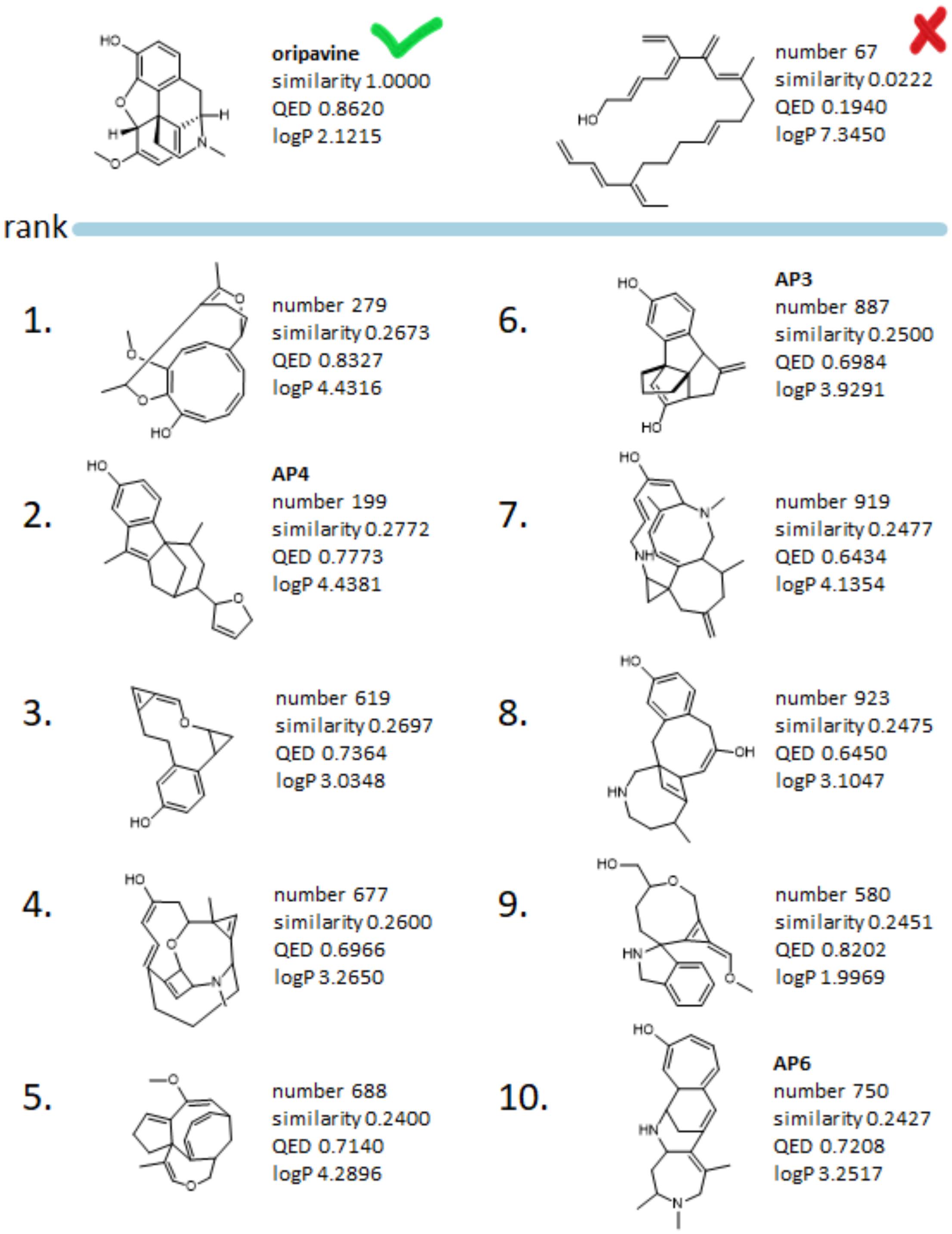

Fig. S13. Ten most oripavine-like assembly products ranked according to their Tanimoto scores. For comparison purposes, oripavine and the least-similar product are shown on top.

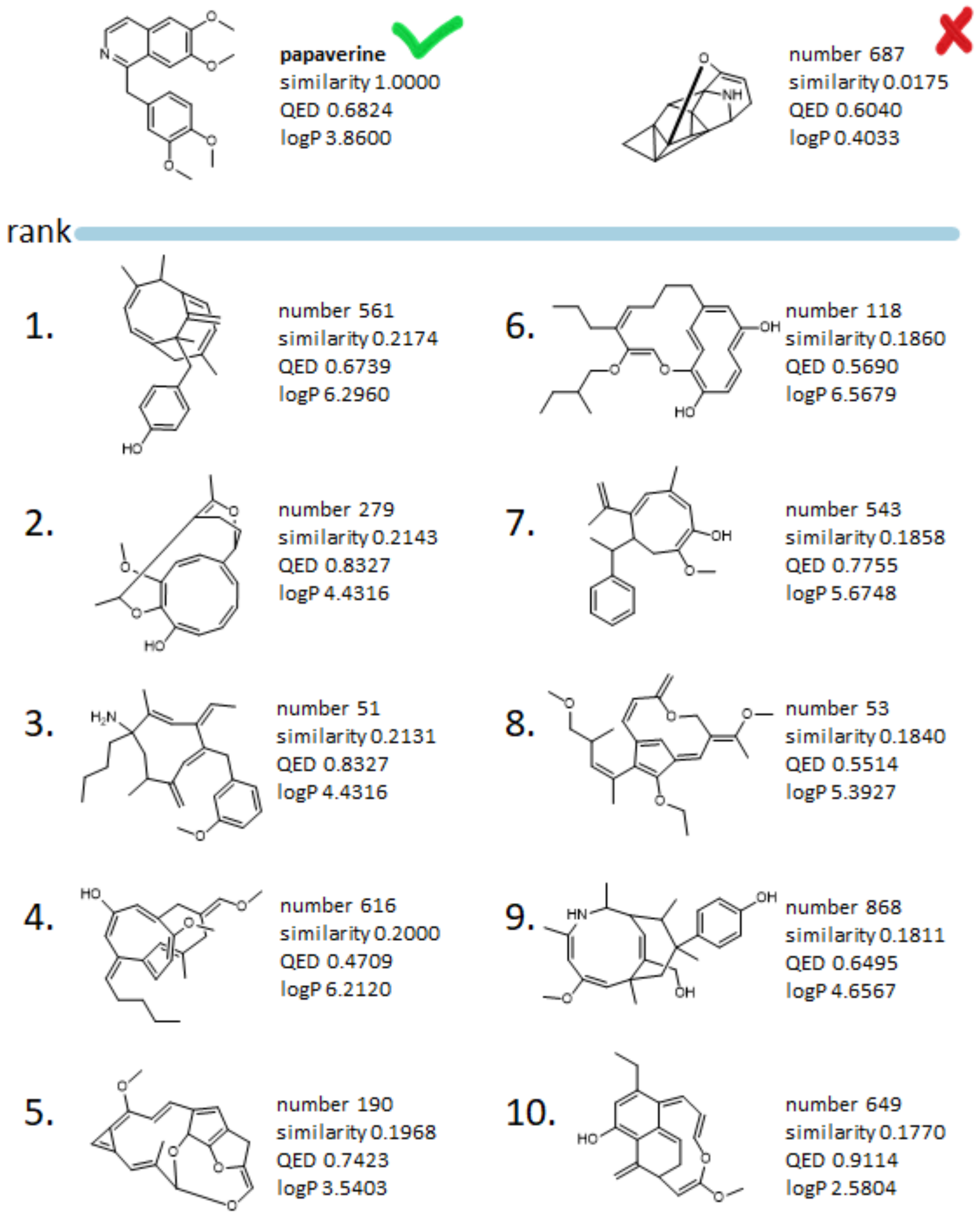

Fig. S14. Ten most papaverine-like assembly products ranked according to their Tanimoto scores. For comparison purposes, papaverine and the least-similar product are shown on top.

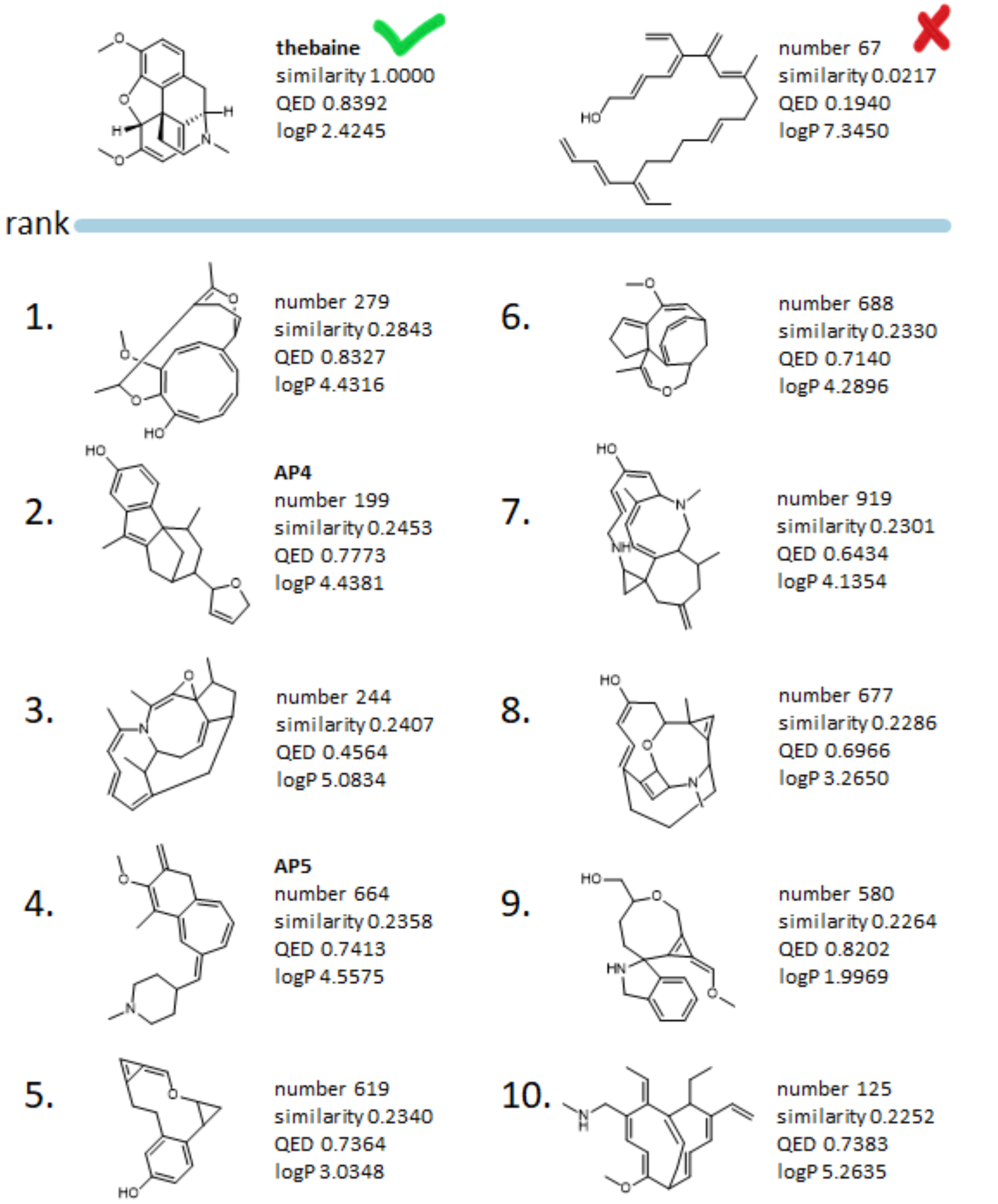

Fig. S15. Ten most thebaine-like assembly products ranked according to their Tanimoto scores. For comparison purposes, thebaine and the least-similar product are shown on top.

compound

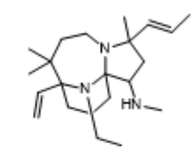

identification
number 161

properties
QED 0.7498
logP 4.4177

similarity
codeine 0.1083
morphine 0.0924
noscapine 0.0915
oripavine 0.1083
papaverine 0.0522
thebaine 0.1240

compound

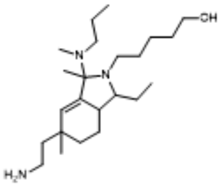

identification
number 438

properties
QED 0.4209
logP 3.9927

similarity
codeine 0.1138
morphine 0.1074
noscapine 0.0816
oripavine 0.1322
papaverine 0.0507
thebaine 0.1290

compound

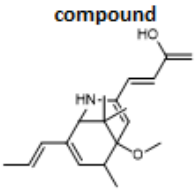

identification
number 165

properties
QED 0.6082
logP 4.0336

similarity
codeine 0.1193
morphine 0.1019
noscapine 0.0909
oripavine 0.1091
papaverine 0.1017
thebaine 0.1062

compound

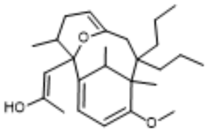

identification
number 547

properties
QED 0.4779
logP 7.2303

similarity
codeine 0.1545
morphine 0.1301
noscapine 0.1233
oripavine 0.2034
papaverine 0.1111
thebaine 0.1983

compound

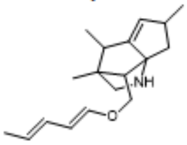

identification
number 275

properties
QED 0.4792
logP 3.6732

similarity
codeine 0.1019
morphine 0.0841
noscapine 0.0846
oripavine 0.1121
papaverine 0.0672
thebaine 0.1193

compound

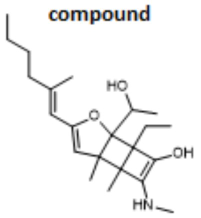

identification
number 548

properties
QED 0.6209
logP 4.5818

similarity
codeine 0.0732
morphine 0.0840
noscapine 0.0621
oripavine 0.0820
papaverine 0.0769
thebaine 0.0714

compound

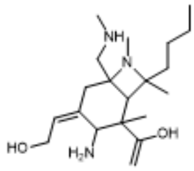

identification
number 382

properties
QED 0.4175
logP 2.1828

similarity
codeine 0.1026
morphine 0.1053
noscapine 0.0863
oripavine 0.0932
papaverine 0.0538
thebaine 0.0909

compound

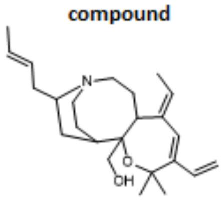

identification
number 561

properties
QED 0.7302
logP 4.6517

similarity
codeine 0.1311
morphine 0.1250
noscapine 0.1034
oripavine 0.1311
papaverine 0.0657
thebaine 0.1280

compound

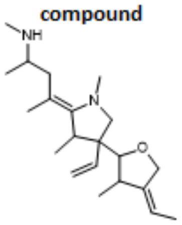

identification
number 403

properties
QED 0.7704
logP 3.9935

similarity
codeine 0.1339
morphine 0.1273
noscapine 0.0956
oripavine 0.1140
papaverine 0.0630
thebaine 0.1111

compound

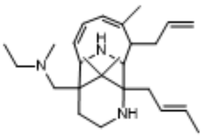

identification
number 616

properties
QED 0.6100
logP 4.6978

similarity
codeine 0.1260
morphine 0.1200
noscapine 0.0855
oripavine 0.1172
papaverine 0.0634
thebaine 0.1231

compound

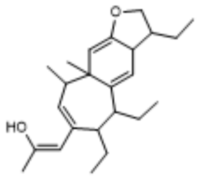

identification
number 435

properties
QED 0.4552
logP 6.5795

similarity
codeine 0.1356
morphine 0.1391
noscapine 0.1064
oripavine 0.1261
papaverine 0.0923
thebaine 0.1230

compound

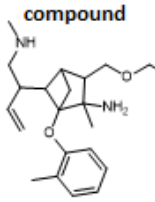

identification
number 657

properties
QED 0.6660
logP 3.1540

similarity
codeine 0.1638
morphine 0.1379
noscapine 0.1377
oripavine 0.1345
papaverine 0.1349
thebaine 0.1597

Fig. S16a. Examples of opiate-like assembly products generated without unsaturation level constraints. The only restrain was molecular mass (between 281 and 368 Da). Identification numbers correspond to the supplementary files (GenMols/DataForPaper/Unconstrained/newMols.txt).

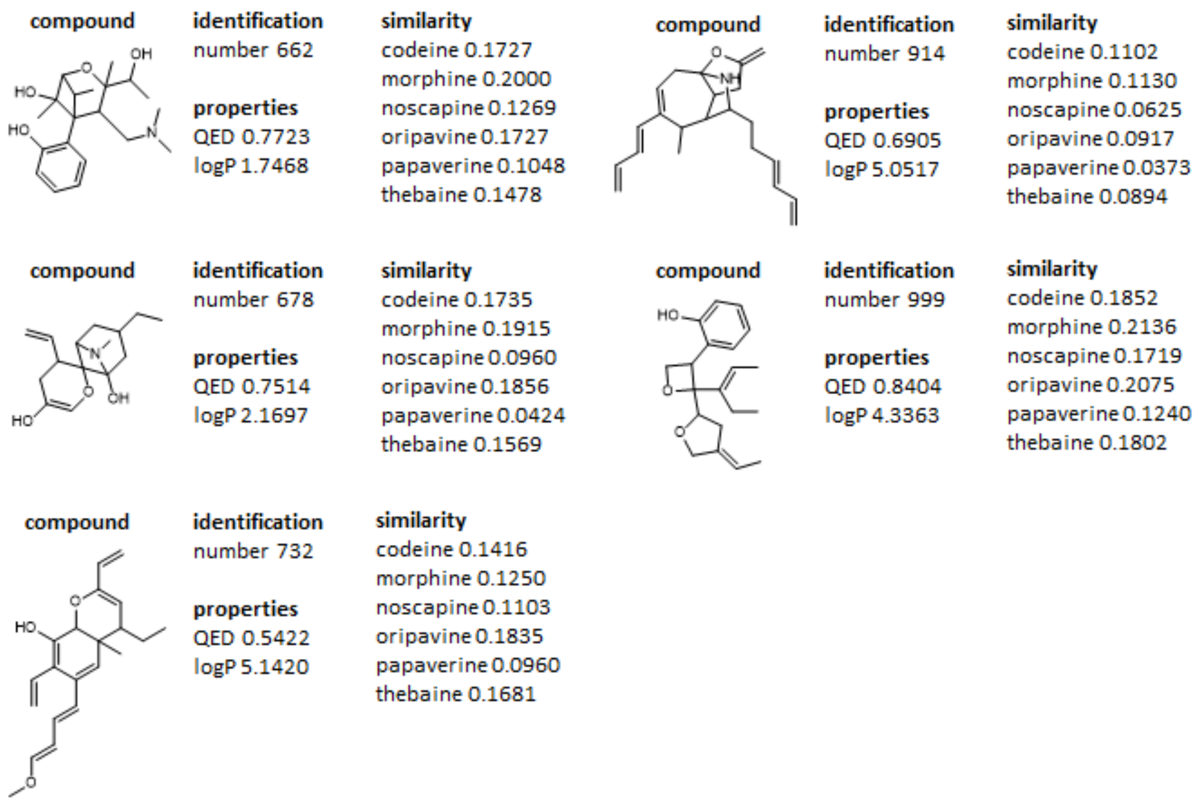

Fig. S16b. Examples of opiate-like assembly products generated without unsaturation level constraints. The only restrain was molecular mass (between 281 and 368 Da). Identification numbers correspond to the supplementary files (GenMols/DataForPaper/Unconstrained/newMols.txt).

8 Comparison with reassembling from arbitrary fragments

In the main text, we have compared our method (denoted as "assembled") with reassembling from only single bonds (denoted as "random"), see Fig. 8 in the main text. Here, we further compare the method of reassembling from arbitrary substructural fragments (denoted as "arbitrary"), i.e., the fragment to be reassembled can be any substructure of the original set of molecules. Any other settings are exactly the same as that in Fig. 8. The results are shown in Fig. S17. We can see that (1) for Tanimoto similarity, the "arbitrary" method performs worse than our proposed "assembled" method (referring to Fig. 8) but better than the "random" method, while (2) for QED measurement, the "arbitrary" method performs worse than our proposed "assembled" method and pretty much the same as the "random" method. To

summarize, our proposed "assembled" method performs better than the "arbitrary" method, as expected.

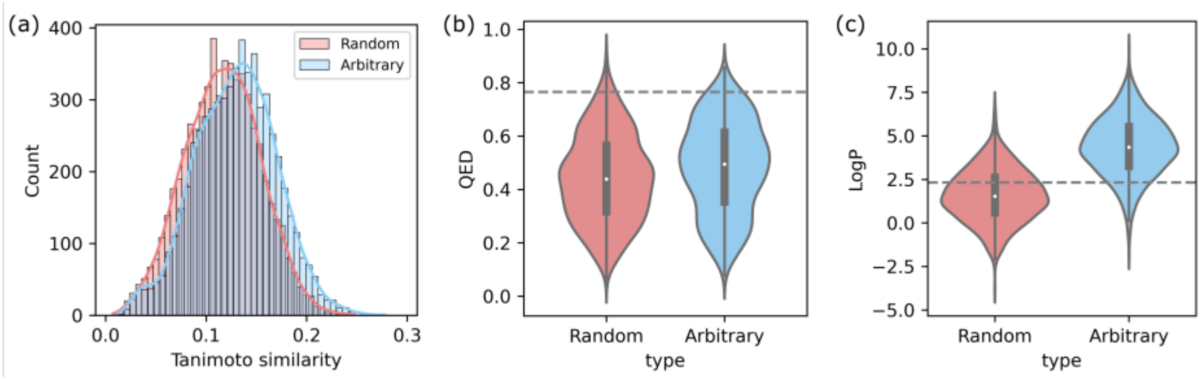

Fig. S17. The comparison of 1000 molecule sets generated from arbitrary substructural fragments of the original set of six opiates (as Fig. 8 in the main text) (blue) and generated from individual bonds (red).

Besides the worse performance, the "arbitrary" method has some other problems. First of all, the number of all possible fragments is much larger, over half a million for this set of 6 opiates for example. When we need to include lots of original molecules in practical drug designs, this would cause a big problem of storing and handling this large amount of data, since, roughly speaking, the number of possible fragments increases exponentially with the number of bonds of molecules. Second, when we were doing the "arbitrary" method *in silico*, it happens very frequently that a new generated molecule is simply a large fragment or probably with an extra single bond attached somewhere (which is very unlikely to happen in the "assembled" method). This is not mysterious because for all possible unique substructures, a large fraction is taken up by large substructures (this is straightforward to see, e.g., for a non-chain molecule, there are many ways to detach two bonds without splitting the molecule into two, and each alternated structure is a valid "substructure"). But these "new" molecules would be no use and we should filter them for good. All in all, the ultimate problem of the "arbitrary" method lies in the fact that the useful information is submerged (practically lost) in the tremendously large set of all possible substructures, while our method is exactly used to extract this useful information.
