# Supplementary material for: Exploring and mapping chemical space with molecular assembly trees
Source: Sci Adv. 2021 Sep 24;7(39):eabj2465. doi: 10.1126/sciadv.abj2465 (PMC8462901; doi:10.1126/sciadv.abj2465)
Supplement: Supplementary file 3 — Data file S1 [file sciadv.abj2465_data_file_s1.zip › code/AssemblyMC.zip!/AssemblyMC/readme.pdf]

Executable program manual (*AssemblyMC.exe*)

There are six files in this folder:

1. *AssemblyMC.exe*, the executable file to calculate the shortest assembly pathways for a single molecule, which can be run on Windows 10 or above.
2. *libinchi.dll*, a dynamic-link library file that is necessary for the exe file to run. Just leave it in the same folder of *AssemblyMC.exe*.
3. *readme.pdf*, the manual of the program.
4. *Adenine.mol*, the mol file (a standard file format to hold the information of a molecule) of the molecule adenine. It is the input of the exe file if you wish to calculate the shortest assembly pathways for adenine. In general, the mol file of a molecule can be freely downloaded from online databases such as ChEMBL, PubChem, etc.
5. *example_Adenine_pathway.txt*, one file of the results, which will be explained later.
6. *example_Adenine_histogram.txt*, one file of the results, which will be explained later.

Keep them in the same folder. Then:

1. Download the mol file of the molecule that you wish to calculate, and put it into the same folder of *AssemblyMC.exe* (here I will just take *Adenine.mol* as an example).
2. Open Windows command prompt (*cmd.exe*), and navigate to this folder.
3. Enter `>AssemblyMC.exe Adenine.mol` and it will then analyze the file *Adenine.mol*, namely, molecule adenine.

   There are two parameters that you can input in order either none (as shown above), one or two of them. The first parameter (*Nstep2*, referring to the paper's SI for details) means how many possible assembly pathways to try in order to find the shortest one (-1 is the default value, meaning never stop); while the second parameter (*Nstep1*, referring to the paper's SI for details) means how many fragmenting schemes to try to obtain the fragments

histogram (-1 is the default value, meaning max(100000, 1% of all possible fragmenting schemes of the molecule)).

The example above has no parameter input. The following example has two parameters input

```
>AssemblyMC.exe Adenine.mol 50000 100000
```

which I will take as an example to show what to expect when it runs and after it finishes. It first calculates the fragments histogram, and shows the process as

```
Please wait...
 .100000 steps to try in step 1...
 >23132 >45035 >67144
```

When the number (e.g., >67144) shown equal to the second parameter, this process to obtain the fragments distribution (Monte Carlo step 1, referring to the paper's SI for details) finishes. And the message below will be displayed in the prompt:

```
 >23132 >45035 >67144 >90217
=============================
====== Step 1 is Done. ======
=============================
```

After that, it will start Monte Carlo step 2 immediately. It will keep displaying the symbol ">" until 10000 pathways have been tried. Then, in the same folder, a file *Adenine_pathway.txt* will be created that is the ultimate result and contains all the information we need, which I will explain how to interpret soon (it will also display some texts in the prompt, which basically repeats what is written in the file *Adenine_pathway.txt*). Note that the program will continue to run until it has tried *Nstep2* (the first parameter you input) number of possible assembly pathways; but each time it has tried 10000 pathways, it will update and overwrite the file *Adenine_pathway.txt*. If the first parameter you input is the default value -1, you may close the program manually when you think it has tried enough possible pathways.

Now I will explain how to interpret the results contained in *Adenine_pathway.txt*. I will take the file *example_Adenine_pathway.txt* as an example to explain due to the fact that this method is Monte Carlo (thus contains randomness) so there might be slight differences at each time it runs. Indeed, *example_Adenine_pathway.txt* is the file *Adenine_pathway.txt* generated for a particular run, and I just renamed it as *example_Adenine_pathway.txt.*

```
Monte Carlo Calculating Pathway Assembly (in bonds). v1.0

The molecule to be analysed is:
InChI=1/C5H5N5/c6-4-3-5(9-1-7-3)10-2-8-4/h1-2H,(H3,6,7,8,9,10)/f/h9H,6H2
    ||  C   #8  -->  8, 10
    ||  C   #0  -->  0, 1, 2
    ||  N   #3  -->  2, 7
    ||  C   #7  -->  7, 9
    ||  C   #1  -->  0, 3, 4
    ||  N   #9  -->  4, 9
    ||  N   #4  -->  3, 8
    ||  N   #5  -->  5, 10
    ||  C   #2  -->  1, 5, 6
    ||  N   #6  -->  6
    ||      0  *1 :  #0 -- #1
    ||      8  *1 :  #4 -- #8
    ||      2  *1 :  #0 -- #3
    ||      10  *2 :  #5 -- #8
    ||      9  *1 :  #9 -- #7
    ||      1  *2 :  #0 -- #2
    ||      7  *2 :  #3 -- #7
    ||      3  *2 :  #1 -- #4
    ||      4  *1 :  #1 -- #9
    ||      5  *1 :  #2 -- #5
    ||      6  *1 :  #2 -- #6

. Total number of atoms: 15
. When hydrogen atoms (H) are excluded:
Number of atoms: 10
Number of bonds: 11

. Parameter Nstep1 = 100000
. Parameter Nstep2 = 50000

Please wait until it generates results...
================================================
============ Printing step 2 results ============
================================================
. 50000 pathways tried.
. Elapsed time: 25.1389 s

Number of pathways: 2
Assembly index is: 7

=================
=== Pathway 1 ===
=================
InChI=1/C2H6N2/c1-4-2-3/h2H,1H3,(H2,3,4)/f/h3H2
InChI=1/C2H5N/c1-3-2/h1H2,2H3

How many times each duplicates:
1 :  (2,7,9)
1 :  (3,8)


=================
=== Pathway 2 ===
=================
InChI=1/C2H6N2/c1-4-2-3/h2H,1H3,(H2,3,4)/f/h3H2
InChI=1/CH4N2/c2-1-3/h1H,(H3,2,3)/f/h2H,3H2

How many times each duplicates:
1 :  (2,7,9)
1 :  (3,4)
```

The block started with "| |" records how the molecule is represented in the program. The lines started with capital letters represent atoms. For example, the first line "C #8 --> 8, 10" means the carbon (C) atom's ID is 8 (denoted by symbol #), and it is attached by bond 8 and 10; the seventh line "N #4 --> 3, 8" means the nitrogen (N) atom's ID is 4, and it is attached by bond 3 and 8. The lines started with integers represent bonds. For example, the first line "0 *1 : #0 -- #1" means this bond's ID is 0, which is a single bond (denoted by symbol *), and it connects atom 0 and atom 1; the fourth line "10 *2 : #5 -- #8" means this bond's ID is 10, which is a double bond, and it connects atom 5 and atom 8. All other information is self-evident.

Now let's look at the lines "Number of pathways: 2" and "Assembly index is: 7". It tells you that the assembly number of this molecule adenine is 7 (namely, the assembly index of the shortest assembly pathways of adenine), and there are two assembly pathways having the same index 7, each of which is displayed in the following, started with "=== Pathway i ===". Take the first pathway as an example, each line started with "InChI=" is an InChI (International Chemical Identifier) of a chemical structure. The first line after "How many times each duplicates:", i.e., "1 : (2, 7, 9)" means that in the bag representation of this pathway (referring to the paper) the count of occurrences should be written as 1 for the first structure listed above "InChI=1/C2H6N2/c1-4-2-3/h2H,1H3,(H2,3,4)/f/h3H2" which is made of bond 2, 7 and 9. The second line "1 : (3, 8)" means that in the bag representation the count of occurrences should be written as 1 for the second structure "InChI=1/C2H5N/c1-3-2/h1H2,2H3" which is made of bond 3 and 8. Therefore, the bag representation of assembly pathway 1 is (InChI=1/C2H6N2/c1-4-2-3/h2H,1H3,(H2,3,4)/f/h3H2, InChI=1/C2H5N/c1-3-2/h1H2,2H3). InChI can be easily transformed to any other format by standard software (e.g., OpenBabel). Here we use OpenBabel to transform the two InChI's into the graph representation of molecules, so this pathway can also be denoted as:

(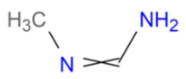 , 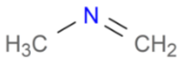)

Note that we should ignore all of the hydrogens as we have ignored them from the beginning (these hydrogens appear in the graphs because we have to use them as some “placeholders” to generate proper InChI’s).

Likewise, the bag representation of assembly pathway 2 is (InChI=1/C2H6N2/c1-4-2-3/h2H,1H3,(H2,3,4)/f/h3H2, InChI=1/CH4N2/c2-1-3/h1H,(H3,2,3)/f/h2H,3H2), or denoted as:

(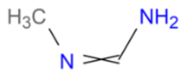 , 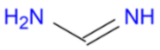)

Lastly, there is another temporary file *Adenine_histogram.txt* also generated, that is automatically used by the program in Monte Carlo step 2, so do not delete it. It displays the fragments histogram obtained in Monte Carlo step 1. It might be useful to the reader, so I will also explain it (I will take *example_Adenine_histogram.txt* as an example). The first part of this file is the same as *example_Adenine_pathway.txt*, while the second half is written as follows. It displays fragments in groups (every five fragments constitute a group) which are sorted by the number of occurrences (namely, y-axis value of the fragments distribution, referring to SI section 4.2 to learn more about this distribution). So, here we see that structure InChI=1/C2H5N/c1-2-3/h2H,1,3H2 appears three times, InChI=1/CH4N2/c2-1-3/h1H,(H3,2,3)/f/h2H,3H2 appears three times, and so on; structure InChI=1/C3H5N/c1-3-4-2/h3H,1-2H2 appears twice, InChI=1/C2H6N2/c1-4-2-3/h2H,1H3,(H2,3,4)/f/h3-4H appears twice, and so on.

```
==================================================
========= Printing fragments histogram ===========
==================================================
Parameter Nstep1 = 100000
Elapsed time: 20.3378 s

InChI=1/C2H5N/c1-2-3/h2H,1,3H2
InChI=1/CH4N2/c2-1-3/h1H,(H3,2,3)/f/h2H,3H2
InChI=1/C3H6N2/c1-5-3-2-4/h2-3H,1,4H2
InChI=1/C2H5N/c1-3-2/h1H2,2H3
InChI=1/C2H6N2/c1-4-2-3/h2H,1H3,(H2,3,4)/f/h3H2
3
3
3
3
3

InChI=1/C3H5N/c1-3-4-2/h3H,1-2H2
InChI=1/C2H6N2/c1-4-2-3/h2H,1H3,(H2,3,4)/f/h3-4H
InChI=1/C3H7N/c1-2-3-4/h2-3H,4H2,1H3
InChI=1/C2H7N/c1-2-3/h2-3H2,1H3
InChI=1/C3H8N2/c1-4-3-5-2/h3H,1-2H3,(H,4,5)/f/h4H
2
2
2
2
2

InChI=1/C2H6N2/c3-1-2-4/h1-2H,3-4H2
InChI=1/C3H6N2/c1-2-5-3-4/h2-3H,1H2,(H2,4,5)/f/h4H2
2
2
```
