# Supplementary material for: Exploring and mapping chemical space with molecular assembly trees
Source: Sci Adv. 2021 Sep 24;7(39):eabj2465. doi: 10.1126/sciadv.abj2465 (PMC8462901; doi:10.1126/sciadv.abj2465)
Supplement: Supplementary file 3 — Data file S1 [file sciadv.abj2465_data_file_s1.zip › code/TreeMC.zip!/TreeMC/readme.pdf]

Executable program manual (*TreeMC.exe*)

In this folder, there are the following files:

1. *TreeMC.exe* (run on Windows 10 or above), the executable file to calculate the molecular assembly tree for a group of molecules.
2. *libinchi.dll*, a dynamic-link library file that is necessary for the exe file to run. Just leave it in the same folder of *TreeMC.exe*.
3. *readme.pdf*, the manual of the program.
4. Mol files: *Adenine.mol*, *Guanine.mol*, *Thymine.mol*, *Cytosine.mol* and *Uracil.mol*, the mol files (a standard file format to hold the information of a molecule) of nucleobases which will be used as the example. They are the required files if you wish to compute the molecular assembly tree of these five nucleobase types. In general, the mol file of a molecule can be freely downloaded from online databases such as ChEMBL, PubChem, etc.
5. *ToDo.txt*, the required user-defined file to feed the exe file, which will be explained in details later.
6. Generated temporary files: *example_Adenine_histWhole.txt*, *example_Adenine _histWhole.txt, example_Adenine_histWhole.txt, example_Adenine_histWhole.txt* and *example_Adenine_histWhole.txt,* the temporary files generated by the program when it is running, which will be explained later.
7. Generated result files: *example_Tree_AllPaths.txt*, *example_Tree_bySize.txt* and *example_Tree_byRepeats.tx,* the files generated at the end that contains all the information we need, which will be explained later.

Now I will show how to run the program, in order to calculate the molecular assembly tree of the five nucleobase types: adenine, guanine, thymine, cytosine and uracil. As described in the paper, it is equivalent to calculate the shortest assembly pathways for these molecules.

1. Put the mol files of these five nucleobases in the folder as *TreeMC.exe*. In general, you could download these mol files from online databases, but here we have already downloaded them for you.
2. Create a plain-text file named *ToDo.txt*, based on the following format strictly (here we have created it for you):

```
Nstep2=30000
Adenine
Nstep1=100000
Thymine
Nstep1=100000
Guanine
Nstep1=100000
Cytosine
Nstep1=100000
Uracil
Nstep1=100000
$
```

   The first line is "Nstep2=XXX" where XXX is the parameter *Nstep2* for the program, meaning how many possible assembly pathways to try in order to find the shortest one (referring to the paper's SI section 4.1 for details). The larger *Nstep2* is, the more accurate the final result will be (but it takes longer time to run). The second line is the name of one mol file: Here it is "Adenine" because the mol file is *Adenine.mol* (note that if the mol file is *abc.mol*, the second line should be "abc"). The third line is "Nstep1=XXX" where XXX is the parameter *Nstep1* for this molecule, meaning how many fragmenting schemes to try to obtain the fragments histogram for this molecule (referring to the paper's SI section 4.1). The larger *Nstep1* is, the more accurate the result will be (but it takes longer time to run). But in any case, we recommend that *Nstep1* should be at least 100000. The 4th and 5th lines are for another molecule (here it is for thymine); the 6th and 7th lines are for another (here it is guanine); and so on. Note that the order of the molecules does not matter, and *Nstep1* can be different for different molecules. Finally, the last line is the symbol "$".

Note that any *Nstep1* could be a special value “0”, for example,

```
Adenine
Nstep1=0
```

which means that the program will use the already-existing histogram file *Adenine_histWhole.txt* instead of calculating it from scratch. If *Nstep1* is not 0, then *Adenine_histWhole.txt* is a temporary file (describing the fragments distribution, see details later) that will be generated when the program is running, and will be used again automatically until the program finishes. If *Nstep1* is set to 0, then the program will not generate /overwrite the file *Adenine_histWhole.txt*, but directly use *Adenine_histWhole.txt* to calculate the tree, which may save a lot of time (in this case, if there is no such file named *Adenine_histWhole.txt* in this folder, the program will report an error and terminate).

3. Simply double click *TreeMC*.exe to run, and wait it to finish, which may take a while.

What happens when the program is running? In the console, it displays the progress as it calculates the fragments distribution, namely, Monte Carlo step 1 (referring to the paper’s SI Fig. S1):

```
Please wait...

 .Adenine:
  100000 steps to try in step 1...
 >16685 >34434 >50908 >68473 >86052


Please wait...

 .Thymine:
  100000 steps to try in step 1...
 >21209 >43578 >66748 >89810


Please wait...

 .Guanine:
  100000 steps to try in step 1...
```

Every time when it obtains the fragments distribution for a molecule, a file named *XXX_histWhole.txt* will be generated or overwritten if already existed, where *XXX* is the name of the corresponding mol file. Just leave them there and they will be automatically reused in

Monte Carlo step 2. When each molecule's fragments distribution have been obtained, it will display a message, and Monte Carlo step 2 will start immediately:

```
=============================================
====== Step 1 is done for all molecules. ======
=============================================

====== Step 2 started. ======
File 1 (Adenine) InChI counts: 787
File 2 (Thymine) InChI counts: 186
File 3 (Guanine) InChI counts: 1432
File 4 (Cytosine) InChI counts: 108
File 5 (Uracil) InChI counts: 102
0:24(24), 1:21(21), 2:26(21), 3:29(21), 4:20(20), 5:23(20), 6:30(20), 7:24(20)
, 8:22(20), 9:23(20), 10:23(20), 11:26(20), 12:20(20), 13:26(20), 14:26(20), 1
5:24(20), 16:28(20), 17:22(20), 18:19(19), 19:20(19), 20:24(19), 21:31(19), 22
:22(19), 23:29(19), 24:27(19), 25:25(19), 26:23(19), 27:24(19), 28:25(19), 29:
26(19), 30:32(19), 31:26(19), 32:24(19), 33:26(19), 34:26(19), 35:28(19), 36:2
7(19), 37:22(19), 38:24(19), 39:22(19), 40:23(19), 41:24(19), 42:26(19), 43:23
(19), 44:24(19), 45:24(19), 46:26(19), 47:20(19), 48:27(19), 49:23(19), 50:24(
19), 51:26(19), 52:26(19), 53:24(19), 54:24(19), 55:21(19), 56:26(19), 57:22(1
9), 58:25(19), 59:22(19), 60:27(19), 61:32(19), 62:24(19), 63:23(19), 64:26(19
), 65:25(19), 66:24(19), 67:19(19), 68:26(19), 69:27(19), 70:25(19), 71:29(19)
```

The message "71: 29 (19)", for example, means that 71 possible pathways have been tried, the assembly index of the previous pathway is 29, and the minimum index till now is 19. This will continue until it checks *Nstep2* number of possible pathways. After that, a message will be displayed (as follows, which means that there are 5 pathways having index 16; 52 pathways having index 17; and so on) and the program finishes (result files will be generated).

```
(16), 29966:24(16), 29967:24(16), 29968:27(16), 29969:28(16), 29970:25(16), 29
971:34(16), 29972:22(16), 29973:25(16), 29974:22(16), 29975:24(16), 29976:22(1
6), 29977:19(16), 29978:28(16), 29979:26(16), 29980:24(16), 29981:19(16), 2998
2:26(16), 29983:21(16), 29984:29(16), 29985:28(16), 29986:22(16), 29987:21(16)
, 29988:29(16), 29989:25(16), 29990:27(16), 29991:22(16), 29992:24(16), 29993:
21(16), 29994:29(16), 29995:22(16), 29996:28(16), 29997:28(16), 29998:27(16),
29999:20(16),
Pathway distribution:
16:5
17:52
18:164
19:430
20:950
21:1738
22:2712
23:3666
24:4341
25:4274
26:3891
27:2928
28:2077
29:1222
30:659
31:335
32:184
33:89
34:32
35:14
36:4
Press any key to continue . . .
```

Now we will explain how to interpret the result files. The file *Tree_AllPaths.txt* is one of the three files generated at the end, which contains all the information we need. Here I will take

example_Tree_AllPaths.txt as an example to explain due to the fact that this method is Monte Carlo (thus contains randomness) so there might be slight differences at each time it runs. Indeed, *example_Tree_AllPaths.txt* is the file *Tree_AllPaths.txt* generated for a particular run, and we just renamed it as *example_Tree_AllPaths.txt*. This is how the file starts with:

```
====================
Index = 16
$
InChI=1/C4H4N2O2/c7-3-1-2-5-4(8)6-3/h1-2H,(H2,5,6,7,8)/f/h5-6H
1, 8
InChI=1/C4H9N3O/c5-2-1-3-7-4(6)8/h1,3H,2,5H2,(H3,6,7,8)/f/h7H,6H2
1, 7
InChI=1/C5H12N6/c6-1-4(10-2-7)5(9)11-3-8/h2-3H,1,6,9H2,(H2,7,10)(H2,8,11)/f/h7-8H2
1, 10
InChI=1/C2H6N2/c1-4-2-3/h2H,1H3,(H2,3,4)/f/h3H2
1, 3
InChI=1/C3H8N2/c4-2-1-3-5/h1-2H,3-5H2
1, 4

$
InChI=1/C5H8N2O2/c1-2-3-6-5(9)7-4-8/h2-4H,1H3,(H2,6,7,8,9)/f/h6-7H
1, 8
InChI=1/C6H13N5/c1-9-3-5(10-2)6(8)11-4-7/h4,9H,2-3,8H2,1H3,(H2,7,11)/f/h7H2
1, 10
InChI=1/C4H9N3O/c5-2-1-3-7-4(6)8/h1,3H,2,5H2,(H3,6,7,8)/f/h7H,6H2
1, 7
InChI=1/C2H6N2/c1-4-2-3/h2H,1H3,(H2,3,4)/f/h3H2
1, 3
InChI=1/C3H8N2/c4-2-1-3-5/h1-2H,3-5H2
1, 4

$
InChI=1/C5H8N2O2/c1-6-3-2-5(9)7-4-8/h2-4,6H,1H3,(H,7,8,9)/f/h7H
1, 8
InChI=1/C5H12N6/c6-1-4(10-2-7)5(9)11-3-8/h2-3H,1,6,9H2,(H2,7,10)(H2,8,11)/f/h7-8H2
1, 10
InChI=1/C4H9N3O/c5-2-1-3-7-4(6)8/h1,3H,2,5H2,(H3,6,7,8)/f/h7H,6H2
1, 7
InChI=1/C3H8N2/c4-2-1-3-5/h1-2H,3-5H2
1, 4
InChI=1/C2H6N2/c1-4-2-3/h2H,1H3,(H2,3,4)/f/h3H2
1, 3
```

The assembly pathways having assembly index 16 are the shortest, so they are shown first. There are 5 pathways having index 16, so they are shown one by one here (it is a long text file, and it shows only 3 pathways here. After index 16, it shows all pathways having index 17, and then 18, and so on). Each pathway stars with the symbol "$". Each pathway is made of chemical structures, represented by these InChI (International Chemical Identifier) strings. The two numbers below each InChI means the counts and the number of bonds of this structure respectively. We see that the first pathway shown above has five InChI's, corresponding to the following five chemical structures respectively (we used one of the standard software OpenBabel to transform the InChI's into graph representations of molecules):

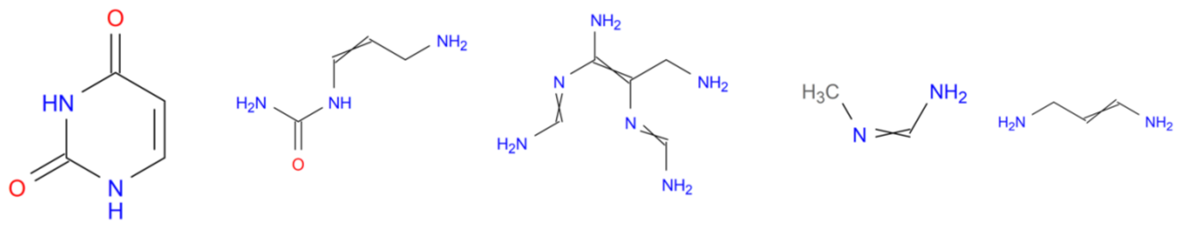

We can also confirm that the first structure has 8 bonds, the second has 7 bonds, and so on. Note that we should ignore all of the hydrogens as we have ignored them from the beginning (these hydrogens appear in the graphs because we have to use them as some "placeholders" to generate proper InChI's). Therefore, the first pathway can be written as the bag represented (as we know, the count "1" has been omitted):

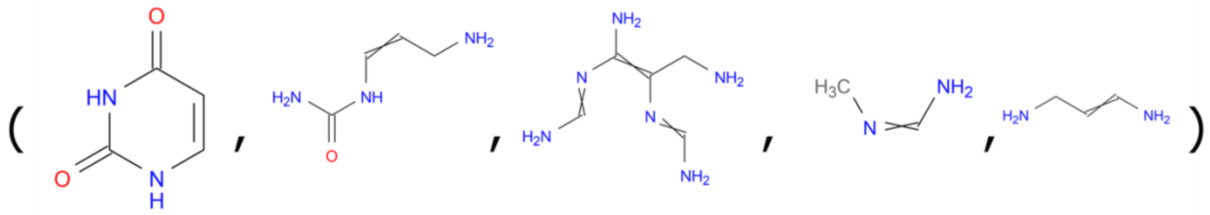

Equivalently, it is the molecular assembly tree of adenine, guanine, thymine, cytosine and uracil (written in bag representation). We can then manually draw the molecular assembly tree of them (see details in the paper's SI):

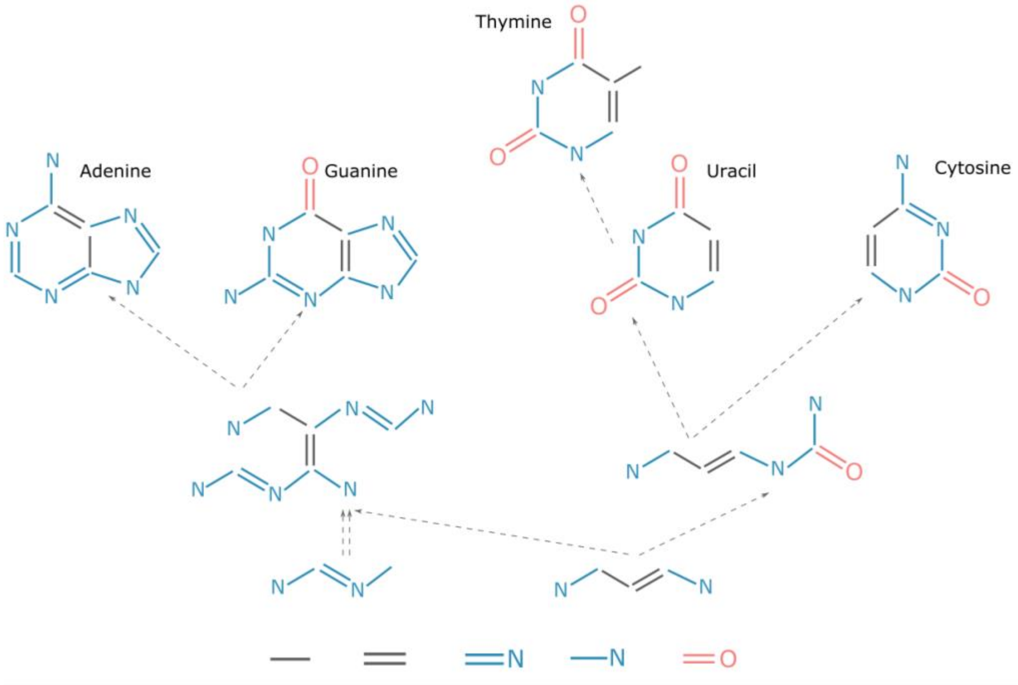

Another file generated at the end is *Tree_bySize.txt* (here we take *example_Tree_bySize.txt* as the example). The information is written as InChI, two numbers, InChI, two numbers, InChI, two numbers, … The first number below an InChI means how many assembly pathways in the file *Tree_AllPaths.txt* contain this chemical structure; and the second number is the number of bonds of this structure. The structures are sorted by the number of bonds (namely the second number). The last file generated at the end is *Tree_byRepeats.txt* (here we take *example_Tree_byRepeats.txt* as the example). It is almost the same as the file *Tree_bySize.txt*, but the structures are sorted by the first number. Some readers may be interested in these information, that's why we generated them.

Finally, we will explain the temporary files (namely, the histogram files, e.g., *Adenine_histWhole.txt*) generated when the program is running. We will take *example_Adenine_histWhole.txt* as an example. The block started with "| |" records how the molecule is represented in the program. The lines started with capital letters represent atoms. For example, the first line "C #8 --> 8, 10" means the carbon (C) atom's ID is 8 (denoted by symbol #), and it is attached by bond 8 and 10; the seventh line "N #4 --> 3, 8" means the nitrogen (N) atom's ID is 4, and it is attached by bond 3 and 8. The lines started with integers represent bonds. For example, the first line "0 *1 : #0 -- #1" means this bond's ID is 0, which is a single bond (denoted by symbol *), and it connects atom 0 and atom 1; the fourth line "10 *2 : #5 -- #8" means this bond's ID is 10, which is a double bond, and it connects atom 5 and atom 8. The information below the symbol "$" is displayed as:

```
$
InChI=1/CH4N2/c2-1-3/h1H,(H3,2,3)/f/h2H,3H2
3:
(3,4,)  (7,9,)  (8,10,)

InChI=1/C2H6N2/c1-4-2-3/h2H,1H3,(H2,3,4)/f/h3H2
3:
(2,7,9,)  (3,4,8,)  (5,8,10,)

InChI=1/C2H5N/c1-2-3/h2H,1,3H2
3:
(1,2,)  (1,5,)  (1,6,)

InChI=1/C3H6N2/c1-5-3-2-4/h2-3H,1,4H2
3:
(1,2,5,10,)  (1,2,5,7,)  (1,2,6,7,)

InChI=1/C2H5N/c1-3-2/h1H2,2H3
3:
(2,7,)  (3,8,)  (5,10,)

InChI=1/C3H7N3/c4-1-2-6-3-5/h1-3H,4H2,(H2,5,6)/f/h5H2
3:
(1,2,5,7,9,)  (1,2,5,8,10,)  (1,2,6,7,9,)
```

Each InChI is a fragment of this molecule, namely, a chemical structure. The number below means how many of this identical structure are contained in this molecule. For example, adenine contains 3 identical structures "InChI=1/CH4N2/c2-1-3/h1H,(H3,2,3)/f/h2H,3H2". Each of the three brackets below shows which bonds constitute this structure. These temporary files are generated because we may only need to calculate the fragments distribution once. For example, if next time we want to calculate the molecular assembly tree of adenine and other molecules, we can put this already-calculated histogram file there, and set *Nstep1* for adenine to 0, the program will then directly take this file rather than calculate it from scratch.
